# 5-HT orchestrates histone serotonylation and citrullination to drive neutrophil extracellular traps and liver metastasis

**DOI:** 10.1172/JCI183544

**Published:** 2025-02-04

**Authors:** Kaiyuan Liu, Yingchao Zhang, Genyu Du, Xinyu Chen, Lingling Xiao, Luyao Jiang, Na Jing, Penghui Xu, Chaoxian Zhao, Yiyun Liu, Huifang Zhao, Yujiao Sun, Jinming Wang, Chaping Cheng, Deng Wang, Jiahua Pan, Wei Xue, Pengcheng Zhang, Zhi-Gang Zhang, Wei-Qiang Gao, Shu-Heng Jiang, Kai Zhang, Helen He Zhu

**Affiliations:** 1 State Key Laboratory of Systems Medicine for Cancer, Renji-Med-X Stem Cell Research Center, Department of Urology, Renji Hospital, Shanghai Cancer Institute, Shanghai Jiao Tong University School of Medicine, Shanghai, China.; 2 Department of Emergency Medicine, Shanghai Seventh People’s Hospital, Seventh People’s Hospital of Shanghai University of Traditional Chinese Medicine, Shanghai, China.; 3 Med-X Research Institute, School of Biomedical Engineering, Shanghai Jiao Tong University, Shanghai, China.; 4 School of Biomedical Engineering, Shanghai Tech University, Shanghai, China.

**Keywords:** Cell biology, Oncology, Prostate cancer

## Abstract

Serotonin (5-HT) is a neurotransmitter that has been linked to tumorigenesis. Whether and how 5-HT modulates cells in the microenvironment to regulate tumor metastasis is largely unknown. Here, we demonstrate that 5-HT was secreted by neuroendocrine prostate cancer (NEPC) cells to communicate with neutrophils and to induce the formation of neutrophil extracellular traps (NETs) in the liver, which in turn facilitated the recruitment of disseminated cancer cells and promoted liver metastasis. 5-HT induced histone serotonylation (H3Q5ser) and orchestrated histone citrullination (H3cit) in neutrophils to trigger chromatin decondensation and facilitate the formation of NETs. Interestingly, we uncovered in this process a reciprocally reinforcing effect between H3Q5ser and H3cit and a crosstalk between the respective writers enzyme transglutaminase 2 (TGM2) and peptidylarginine deiminase 4 (PAD4). Genetic ablation or pharmacological targeting of TGM2, or inhibition of the 5-HT transporter (SERT) with the FDA-approved antidepressant drug fluoxetine reduced H3Q5ser and H3cit modifications, suppressed NET formation, and effectively inhibited NEPC, small-cell lung cancer, and thyroid medullary cancer liver metastasis. Collectively, the 5-HT–triggered production of NETs highlights a targetable neurotransmitter/immune axis that drives liver metastasis of NE cancers.

## Abstract 2

5-HT secreted by neuroendocrine prostate cancer (NEPC) promotes the formation of neutrophil extracellular traps (NETs) via orchestrating histone citrullination and serotonylation to drive liver metastasis.

## Graphical abstract

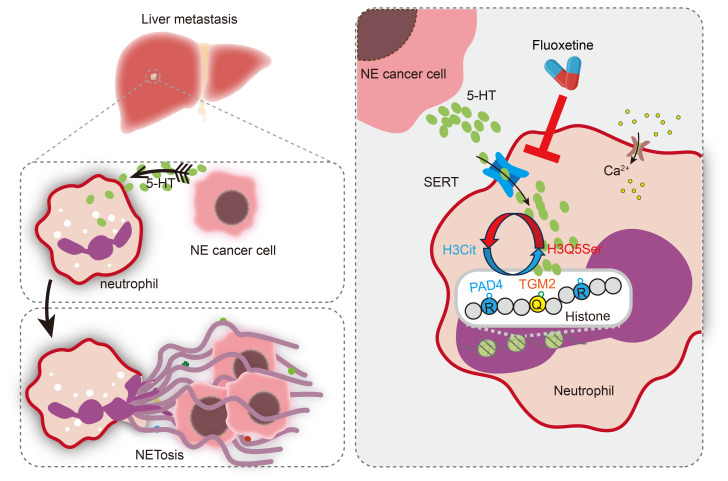

## Introduction

Apart from the classical role of neurotransmitters in neuronal activity, these neurotransmitters have long been implicated in the pathological process of different tumor types (1). Serotonin (5-HT) is a neurotransmitter that is positively involved in cancer development by promoting cancer cell proliferation and invasion (2–4). The precise manner in which 5-HT modulates the tumor microenvironment to regulate metastasis remains largely unknown. Recently, it has been reported that 5-HT can be taken up into cells via the serotonin transporter (SERT) and be covalently added to the glutamine residue of target proteins such as small guanosine triphosphate hydrolases (GTPases) and glyceraldehyde-3-phosphate dehydrogenase (GAPDH) by the enzyme transglutaminase 2 (TGM2) to regulate platelet granule release and CD8^+^ T cell activity, respectively (5–7). Whether 5-HT plays a role in innate immune cells from the tumor microenvironment to regulate cancer progression remains to be elucidated.

Neutrophils are the most abundant cell type in innate immunity. In response to pathogen invasion, neutrophil formed neutrophil extracellular traps (NETs), are web-like structures composed of decondensed chromatin and antimicrobial proteins to kill pathogens (8). Intriguingly, recent studies in cancers suggested that NETs act to promote metastasis by attracting circulating tumor cells or activating dormant cancer cells. Neutrophil chromatin decondensation is a required process for NET formation (9–11). Serotonylation in the fifth glutamine residue of histone H3 (H3Q5ser) has been recently found to participate in chromatin dynamics regulation to activate a permissive transcriptional state (6). Therefore, we hypothesized that 5-HT and H3Q5ser may play a role in the formation of NETs. Crosstalk between different histone modifications has been recently demonstrated to be essential in the intricate regulation of chromatin dynamics (12). Histone citrullination (H3cit) catalyzed by PAD4 is reported to be critical for chromatin decondensation in NETs (11). Whether H3cit interacts with other histone modifications such as H3Q5ser in the process of NET formation is an interesting but unexplored question.

Metastasis is the major cause of death in prostate cancer (PCa) (13, 14). Accumulating evidence shows that the liver, a severely understudied target organ in PCa, is frequently colonized by advanced PCa, especially by the most lethal neuroendocrine prostate cancer (NEPC) variant with a rate of more than 45% (15, 16). These patients with liver metastases had the worst median overall survival (17). We have previously generated a murine NEPC orthotopic implantation model with a high penetrance of liver metastasis (18). Accumulation of neutrophils in the liver of this NEPC tumor–bearing mouse model was interestingly found at the early stage of liver metastasis (18). NEPC displays features of NE cells including the production of neuroactive peptides and neurotransmitters such as 5-HT (2, 19, 20). We asked whether disseminated NEPC communicates with neutrophils in the liver via 5-HT to shape a metastasis-favorable microenvironment.

Here, we observed that 5-HT induced TGM2-mediated H3Q5ser and enhanced PAD4-catalyzed H3cit to drive the formation of NETs. NEPC-derived 5-HT promoted the prostate-to-liver metastasis by crosstalk with hepatic neutrophils.

## Results

### Neutrophil infiltration and NET formation at the early stage promote NEPC liver metastasis.

**Figure 1:**
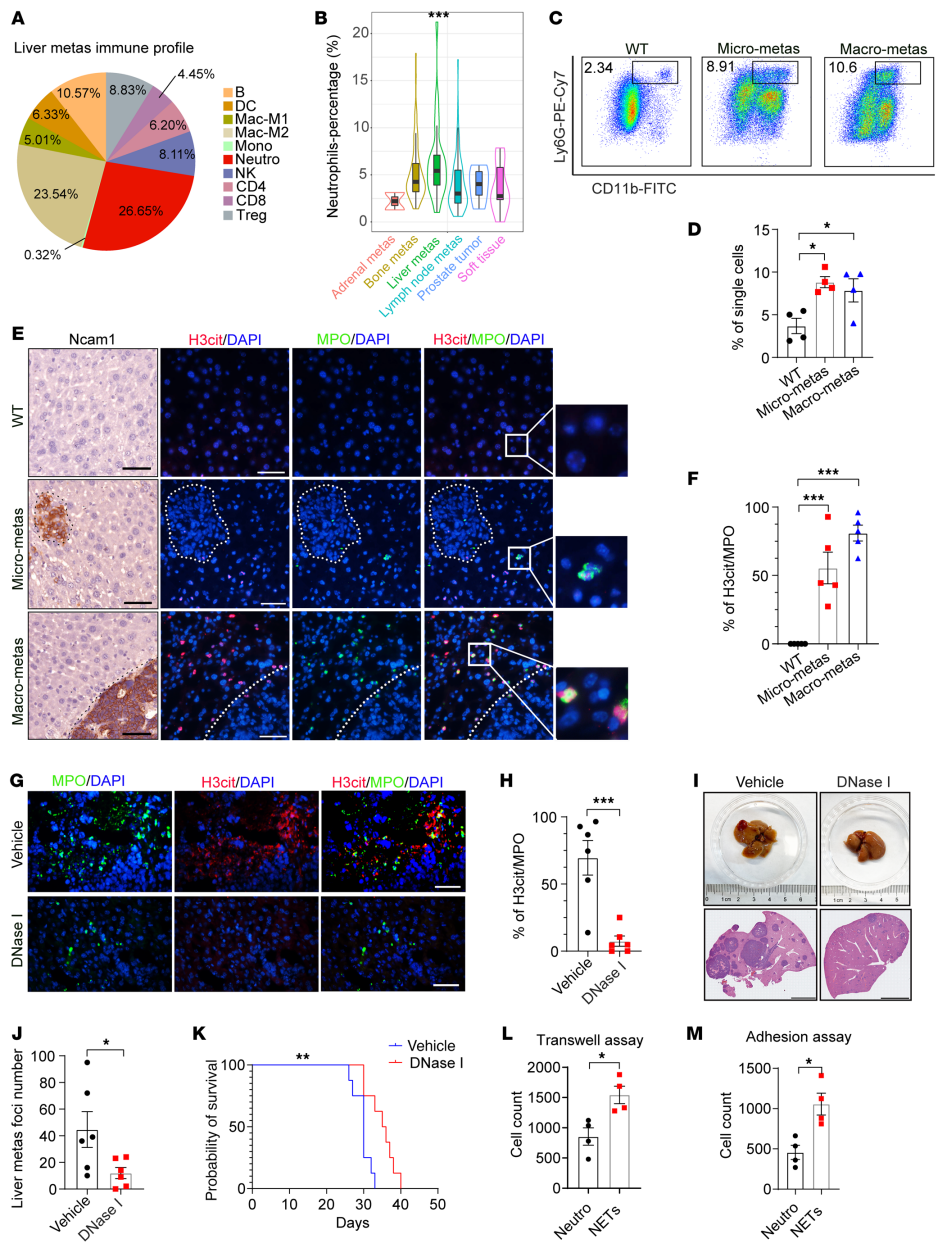
Neutrophil infiltration and NETs are detected in NEPC liver metastasis. (**A**) Immune cell profiles of liver metastases (metas) based on the SU2C PCa dataset (*n* = 26). Neutro, neutrophils; Mac-M1, M1 macrophages; Mac-M2, M2 macrophages; Mono, monocytes. (**B**) Neutrophils are the most enriched immune cell component in liver metastases based on the SU2C dataset (adrenal metastases, *n* = 2; bone metastases, *n* = 82; liver metastases, *n* = 26; lymph node metastases, *n* = 79; prostate tumor, *n* = 5; soft tissue, *n* = 14). (**C** and **D**) Flow cytometric plots (**C**) and quantification (**D**) of CD11b^+^Ly6G^+^ neutrophils in liver (*n* = 4 mice). (**E** and **F**) IHC and IF staining (**E**) and quantification (**F**) of NETs in liver (*n* = 5 mice). Scale bars: 50 μm; 25μm (inserts); original magnification, ×9 (inserts). (**G** and **H**) IF staining (**G**) and quantification (**H**) of NETs (H3cit^+^) in MPO^+^ neutrophils in livers from vehicle- and DNase-I–treated NEPC tumor–bearing mice (*n* = 6 mice). Scale bar: 50 μm. (**I** and **J**) H&E-stained images (**I**) of livers from vehicle- and DNase-I–treated NEPC tumor–bearing mice (scale bars: 4 mm) and quantification (**J**) of liver metastases following vehicle or DNase-I treatment of mice inoculated by i.v. injection of *Rb1^Δ/Δ^* *Trp53^Δ/Δ^* organoids (*n* = 6 mice). (**K**) Survival curves for vehicle- and DNase-I–treated NEPC tumor–bearing mice (*n* = 8 mice). **P < 0.01, by log-rank test. (**L**) Quantification of migrated *Rb1^Δ/Δ^* *Trp53^Δ/Δ^* PCa cells in Boyden chambers recruited by murine primary neutrophils or NETs (*n* = 4 biological replicates). (**M**) Quantification of *Rb1^Δ/Δ^* *Trp53^Δ/Δ^* PCa cells adhesion in murine primary neutrophils or NETs (*n* = 4 biological replicates). *P < 0.05, **P < 0.01, and ***P < 0.001, by 2-tailed Student’s *t* test (**H**, **J**, **L**, and **M**), log-rank test (**K**), 1-way ANOVA followed by Tukey’s test (**D** and **F**), and 1-way ANOVA followed by Dunnett’s test (**B**). Data are shown as the mean ± SEM.

To determine how immune microenvironment regulates metastasis in the liver, we analyzed the immune cell profile in NEPC liver metastasis via the multi-omics–based computational tool Multi-omics Immuno-Oncology Biological Research (IOBR), on a previously reported Stand Up to Cancer (SU2C) dataset (21–23). As shown in Figure 1A, neutrophils were the most abundant immune cells in liver metastases of patients with PCa. We then compared the neutrophil enrichment among different PCa metastatic sites using the SU2C dataset and found a predominant neutrophil infiltration in liver metastases compared with metastatic lesions in other distant sites (Figure 1B). We previously developed an NEPC liver metastasis mouse model, in which the *PbCre*^+^ *Rb1^fl/fl^ Trp53^fl/fl^* (referred to hereafter as *Rb1^Δ/Δ^ Trp53^Δ/Δ^*) tumor organoid was inoculated into the prostate of WT C57BL/6 mice (18). Consistently, flow cytometric analysis of this model revealed that Cd11b^+^Ly6G^+^ neutrophils accumulated in livers with micrometastases of less than 200 μm in diameter, suggesting an increase in neutrophils at the early stage of liver metastasis (Figures 1, C and D).

The production of NETs is an important function of neutrophils. We performed IHC staining against a well-established NE differentiation marker, neural cell adhesion molecule 1 (Ncam1), and conducted immunofluorescence (IF) costaining against myeloperoxidase (MPO), a neutrophil marker, and H3cit, a NETs marker, on serial sections of normal WT liver and liver metastases from the *Rb1^Δ/Δ^* *Trp53^Δ/Δ^* organoid–implanted mice (Figure 1E). Quantification of H3cit and MPO double-positive cell frequency showed significantly increased NET formation from micro to macro liver metastases in *Rb1^Δ/Δ^* *Trp53^Δ/Δ^* NEPC tumor–bearing mice in contrast to normal liver samples from WT mice (Figure 1F).

Next, we determined the formation of NETs in both primary prostate tumors and liver metastases in patients with NEPC. As shown in Supplemental Figure 1, A and B, CD66^+^ neutrophil–derived NETs (H3cit^+^) were much more enriched in liver metastases compared with primary sites. To determine whether hepatic NETs contributed to NEPC metastasis, we injected DNase I to digest NETs in *Rb1^Δ/Δ^* *Trp53^Δ/Δ^* NEPC tumor–bearing mice. Treatment of DNase I degraded NET formation (Figure 1, G and H) and significantly reduced the number of liver metastatic foci (Figure 1, I and J). Notably, DNase I treatment significantly prolonged the survival of *Rb1^Δ/Δ^* *Trp53^Δ/Δ^* NEPC tumor–bearing mice compared with vehicle control mice (Figure 1K). Next, we investigated the effect of NETs on the attraction and retention of NEPC cells. To do this, the bone marrow–derived neutrophils (BMDNs) were isolated from WT C57BL/6 mice, and stimulated with the NETs inducer phorbol 12-myristate 12-acetate (PMA). Compared with the vehicle control, NETs formed from PMA-stimulated murine BMDNs significantly enhanced the migration (Figure 1L) and adhesion (Figure 1M) of *Rb1^Δ/Δ^* *Trp53^Δ/Δ^* NEPC cells. Collectively, these data suggest that recruitment of neutrophils and NET formation promote the liver metastasis in NEPC.

### 5-HT secreted from NEPC potentiates NET formation and NEPC liver metastasis.

5-HT is a well-characterized neurotransmitter, produced by brain serotonergic neurons, enterochromaffin cells and NE tumors (24). Several lines of evidence have shown that NE cancer cells, such as NEPC cells, produce high levels of 5-HT (20, 24). In support of this, we analyzed the RNA-Seq data from the Beltran’s prostate cancer dataset (25) and found that the 5-HT biosynthesis enzyme tryptophan hydroxylase 1 (TPH1) was markedly upregulated in NEPC compared with other PCa subtypes (Supplemental Figure 1C; supplemental material available online with this article; https://doi.org/10.1172/JCI183544DS1). These findings led us to explore whether NEPC-secreted 5-HT can affect NET formation and NEPC liver metastasis.

**Figure 2:**
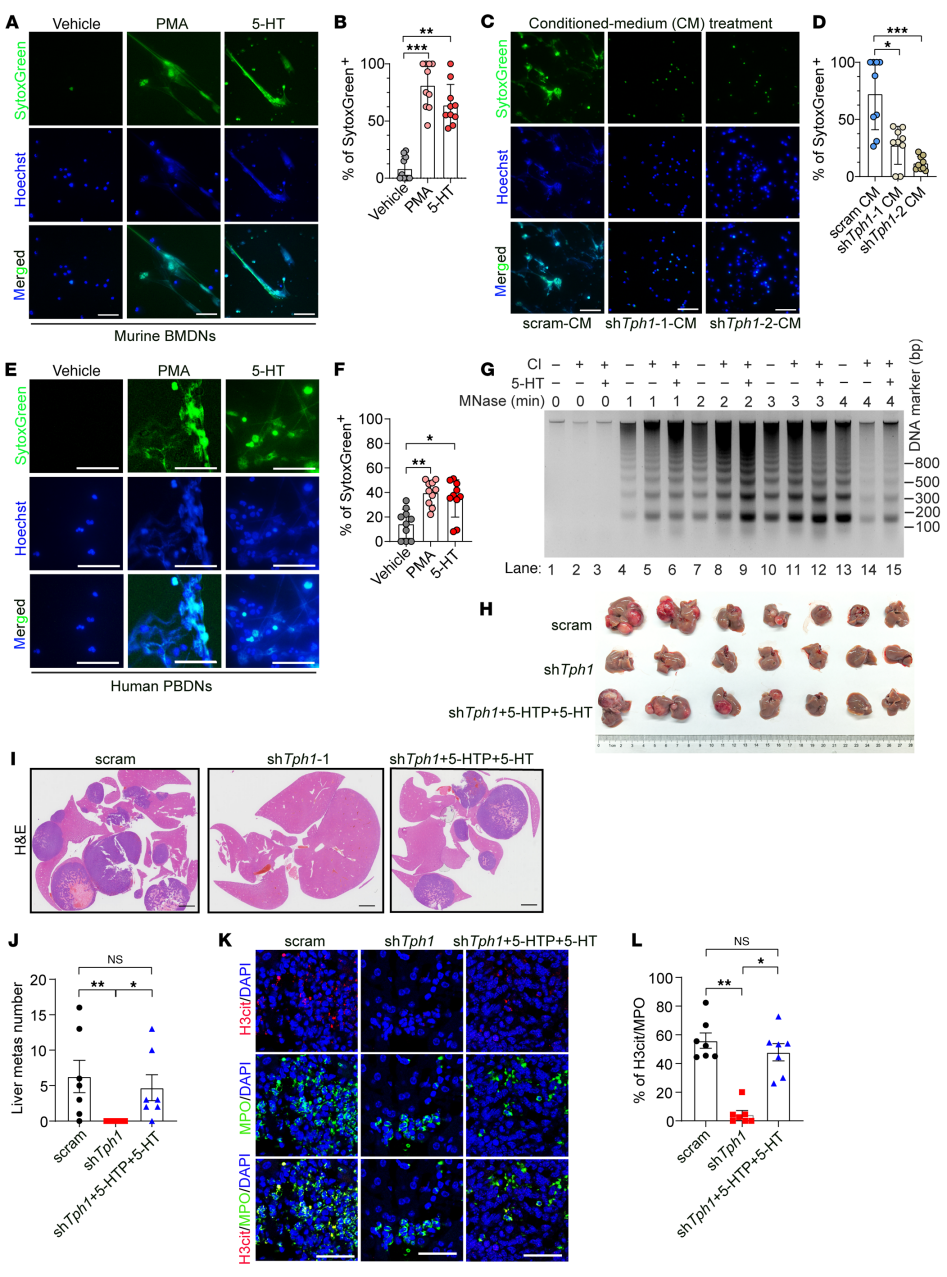
NEPC-derived 5-HT potentiates the formation of NETs and liver metastasis. (**A** and **B**) IF staining images (**A**) and quantification results (**B**) showing NET formation in murine BMDNs in response to 5-HT or vehicle control treatment (*n* = 10 biological replicates). PMA-induced NET formation and served as a positive control. Scale bars: 50 μm. (**C** and **D**) IF staining images (**C**) and quantification results (**D**) showing NET formation in murine BMDNs upon the treatment of CM collected from scrambled- and sh*Tph1*-infected *Rb1^Δ/Δ^* *Trp53^Δ/Δ^* organoids (*n* = 9 biological replicates). Scale bars: 50 μm. (**E** and **F**) IF images (**E**) and quantification (**F**) of the NET-forming capacity of human PBDNs upon 5-HT or vehicle control treatment (*n* = 10 biological replicates). Scale bars: 50 μm. (**G**) MNase digestion assay showing that addition of 5-HT induced decondensed NET chromatin in HL-60 granulocytes compared with calcium ionophore treatment. (**H**–**J**) In vivo experiments demonstrating a reduction of liver metastatic burden (**H**) in sh*Tph1*-infected versus scrambled shRNA–treated mice, and a regained liver metastatic burden in 5-HTP–treated, sh*Tph1*–infected *Rb1^Δ/Δ^* *Trp53^Δ/Δ^* organoid–implanted mice via i.v. injection (*n* = 7 mice, each group). H&E staining (**I**) and quantification data (**J**) validated the significant decrease in liver metastatic foci in *Tph1*-KD *Rb1^Δ/Δ^* *Trp53^Δ/Δ^* organoid–inoculated mice and regained liver metastatic foci numbers in 5-HTP–treated mice. Scale bars: 2 mm. (**K** and **L**) IF images of liver sections (**K**) and quantification results (**L**) revealing a significant decline in NET formation in *Tph1*-KD *Rb1^Δ/Δ^* *Trp53^Δ/Δ^* organoid–inoculated mice (*n* = 7 mice, each group). *P < 0.05, **P < 0.01, and ***P < 0.001, by Kruskal-Wallis test followed by Dunnett’s test (**B**, **D**, **F**, and **L**) and 1-way ANOVA followed by Tukey’s test (**J**). Data are presented as the mean ± SEM.

To this end, we treated murine BMDNs with 5-HT and used a potent inducer of NETs, PMA, as a positive control (26). The cell-impermeant dye SytoxGreen, which can bind to DNA released by neutrophils, was stained and imaged to serve as a measurement of NETs. We found that the number of NETs formed by 5-HT–stimulated neutrophils was similar to that induced by PMA (Figure 2, A and B). We next collected the conditioned medium (CM) from scrambled shRNA (scram-CM) and sh*Tph1*-infected (sh*Tph1*-CM) *Rb1^Δ/Δ^* *Trp53^Δ/Δ^* NEPC organoids in which 5-HT production was blocked to stimulate murine BMDNs, respectively, (Supplemental Figure 1, D and E). In contrast to scram-CM, sh*Tph1*-CM incurred less production of NETs, as evidenced by the reduced percentages of SytoxGreen^+^ cells (Figure 2, C and D) and H3cit^+^ neutrophils (Supplemental Figure 1, F and G). To confirm whether these results were applicable to the human context, we purified human peripheral blood–derived neutrophils (PBDNs) and observed increased NET formation of human PBDNs in response to 5-HT stimulation (Figure 2, E and F, and Supplemental Figure 1, H and I). NET-DNAs have a decondensed structure compared with intact chromatin DNAs, making them more susceptible to digestion into smaller fragments by micrococcal nuclease (MNase) (11). HL-60 cells can be induced to differentiate into granulocytes by *N*,*N*-dimethylformamide (DMF). Upon calcium ionophore treatment, HL-60–derived granulocytes release long stretches of decondensed chromatin and form NETs (11). We therefore conducted MNase digestion assay on HL-60 granulocytes and found that addition of 5-HT accelerated the process of MNase-mediated DNA digestion compared with the calcium ionophore treatment alone (Figure 2G). These data indicate that 5-HT derived from NEPC can induce the formation of NETs.

For in vivo experiments, the scrambled- and sh*Tph1*-transfected *Rb1^Δ/Δ^* *Trp53^Δ/Δ^* NEPC organoids were i.v. inoculated into WT C57BL/6 mice. A significantly reduced liver metastatic burden was observed in the mice that received the *Tph1-*knockdown (*Tph1*-KD) organoids (Figures 2, H–J). On the other hand, the attenuated liver metastasis induced by *Tph1*-KD NEPC organoids was reversed upon 5-HT reconstitution (Figure 2, H–J), suggesting the crucial role of 5-HT in promoting NEPC liver metastasis. Co-IF staining for H3cit and MPO revealed that *Tph1* KD in NEPC organoids led to suppressed hepatic NET formation in nearby areas of metastatic cancer cells compared with scrambled shRNA–transfected counterparts (Figure 2, K and L). Interestingly, the attenuated NET formation (H3cit^+^MPO^+^ signal) induced by *Tph1*-KD organoids was restored by 5-HT reconstitution in vivo (Figure 2, K and L).

To test whether 5-HT is also important for liver metastasis in other NE cancer types, we conducted in vivo experiments using human small cell lung cancer (SCLC) NCI-H82 cells (Supplemental Figure 2, A–E) and human medullary thyroid cancer TT cells (Supplemental Figure 2, F–J). Consistent with the data from the NEPC liver metastasis model, *TPH1* KD in either NCI-H82 or TT cells resulted in significantly suppressed liver metastasis (Supplemental Figure 2, A–C and F–H) and decreased NET formation (Supplemental Figure 2, D, E, I, and J) in both NCI-H82 and TT xenograft models. Collectively, *Tph1*-mediated 5-HT synthesis played an important role in promoting the liver metastasis of pleiotropic NE malignancies, including NEPC, SCLC, and medullary thyroid cancer by facilitating NET formation.

### TGM2-mediated H3Q5ser promotes NET formation.

**Figure 3:**
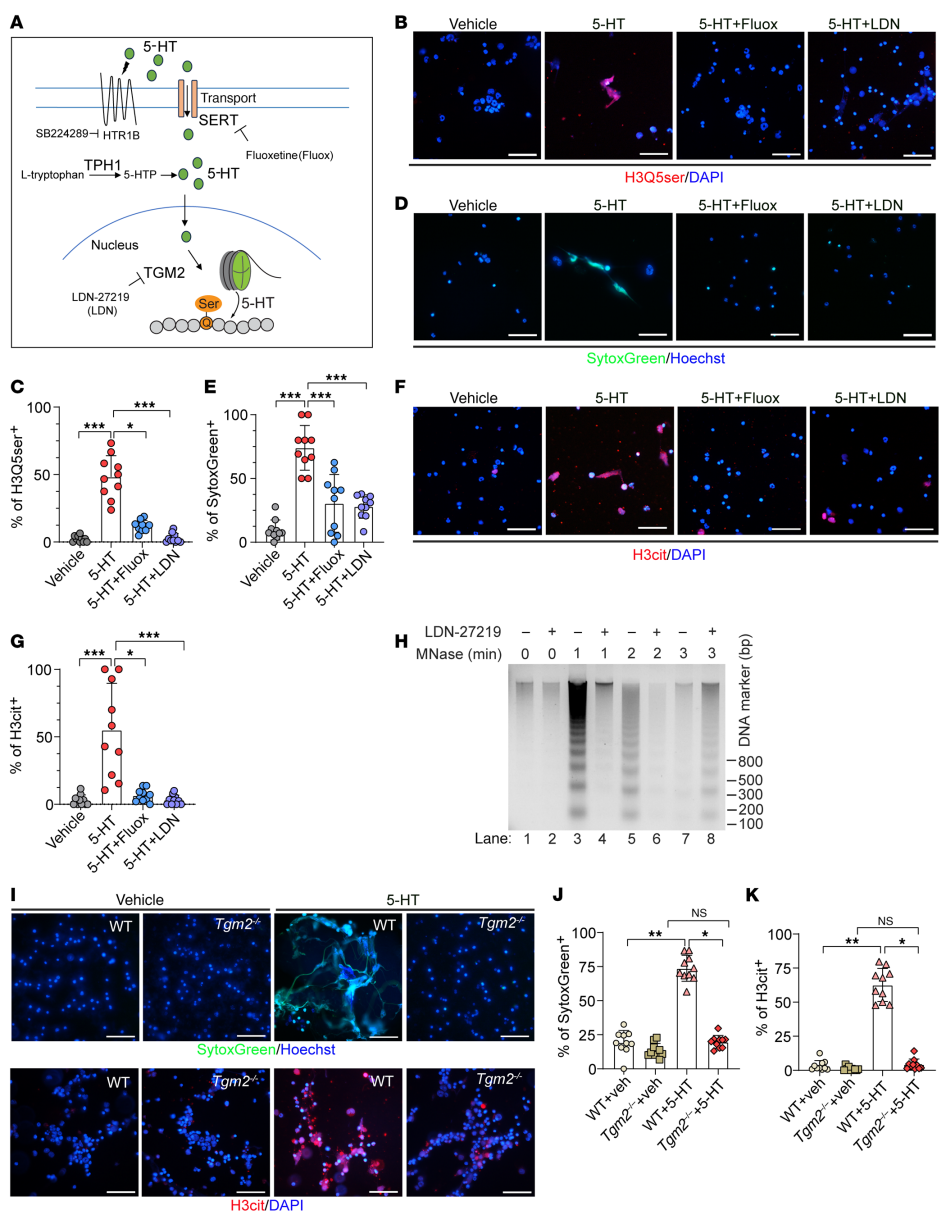
5-HT induces histone serotonylation in neutrophils during NET formation. (**A**) Schematic showing 5-HT–activated 5-HT/HTR signaling, SERT-mediated 5-HT intake, TPH1-catalyzed 5-HT-biosynthesis, and TGM2-catalyzed H3Q5ser. (**B** and **C**) IF staining images (**B**) and quantification results (**C**) demonstrate that addition of 5-HT promoted H3Q5ser modification in murine BMDNs and that the SERT inhibitor Fluox and the TGM2 inhibitor LDN compromised 5-HT–induced H3Q5ser (*n* = 10 biological replicates). Scale bars: 50 μm. (**D**–**G**) IF staining results (**D** and **F**) and quantification data (**E** and **G**) revealed that Fluox and LDN abrogated 5-HT–induced NET formation, as reflected by SytoxGreen (**D** and **E**) and H3cit (**F** and **G**) signals (*n* = 10 biological replicates per experiment). Scale bars: 50 μm. (**H**) The MNase digestion assay showed that LDN treatment of HL-60 granulocytes led to delayed chromatin decondensation compared with vehicle control upon stimulation with the calcium ionophore. (**I**–**K**) *Tgm2^–/–^* mouse–derived BMDNs showed significantly reduced NET formation upon 5-HT stimulation, as exemplified by reduced SytoxGreen (**I** and **J**) and H3cit (**I** and **K**) signals compared with WT mouse–derived BMDNs (*n* = 10 biological replicates per experiment). Scale bars: 50 μm. *P < 0.05, **P < 0.01, and ***P < 0.001, by Kruskal-Wallis test followed by Dunnett’s test (**C**, **G**, **J**, and **K**) and 1-way ANOVA followed by Tukey’s test (**E**). Data are presented as the mean ± SEM.

5-HT could either activate 5-HT receptors (HTRs) or be transported into the cell through the SERT (Figure 3A). When transported into the nucleus, 5-HT can covalently bind to histone catalyzed by TGM2, leading to histone H3Q5ser modification (Figure 3A). This modification has recently been shown to increase chromatin accessibility (27). Therefore, we asked whether H3Q5ser modulates chromatin status in neutrophils and participates in the formation of NETs. To this end, murine BMDNs were induced for NETs production by 5-HT or PMA as a positive control. IF staining for H3Q5ser, SytoxGreen, and H3cit was done to evaluate H3Q5ser levels and NET formation. The results showed that 5-HT treatment led to an increase in positive H3Q5ser signals with a concomitant incremental increase in NET formation and H3cit levels in murine BMDNs (Figure 3, B–G) and human PBDNs (Supplemental Figure 3A). Next, the SERT inhibitor fluoxetine (Fluox) and the TGM2 inhibitor LDN-27219 (LDN) were used to block either 5-HT intake or H3Q5ser modification in neutrophils (Figure 3A). IF staining data suggested that 5-HT–promoted H3Q5ser modification in murine BMDNs was inhibited by either Fluox or LDN (Figure 3, B and C). In line with the decrease in H3Q5ser levels, we also detected an attenuation of NET formation, as exemplified by reductions in SytoxGreen (Figure 3, D and E) and H3cit (Figure 3, F and G) signals in response to either Fluox or LDN treatment. We then tested whether TGM2 facilitates NET-DNA decondensation. The MNase Digestion Assay revealed that suppression of TGM2 resulted in a MNase hyposensitivity profile, which was indicative of a less open chromatin architecture (Figure 3H). Next, we isolated murine BMDNs from *Tgm2*-KO (*Tgm2^–/–^*) mice. Immunoblotting data revealed a concomitant decrease in H3Q5ser and H3cit modifications upon the genetic ablation of *Tgm2* (Supplemental Figure 3B). Compared with WT murine BMDNs, IF staining images showed that 5-HT–induced NET formation was significantly suppressed in *Tgm2^–/–^* murine BMDNs (Figure 3, I–K). These data suggest a critical role of TGM2 in chromatin decondensation and NET formation.

In addition to the 5-HT/SERT signaling, we found that 5-HT could also bind and activate its corresponding receptor, HTR, to initiate downstream signaling cascades (Figure 3A). We therefore asked whether 5-HT/HTR signaling is also involved in H3Q5ser modification and/or NET formation as well. According to previous literature, HTR1B has been reported to be highly expressed in neutrophils (28). We also reanalyzed Beltran’s clinical prostate cancer bulk RNA-Seq data on NEPC liver metastasis (25) and found that the HTR1B was the most highly expressed HTR at the mRNA level in NEPC liver metastatic biospecimens (Supplemental Figure 4A). We therefore blocked HTR1B activity in murine neutrophils using the HTR1B inhibitor SB224289 (Figure 3A) and found that 5-HT–induced H3Q5ser modification (H3Q5ser^+^ signal) and NET formation (SytoxGreen^+^ and H3cit^+^ signals) were not significantly affected upon HTR1B inhibition (Supplemental Figure 4, B–E).

**Figure 4:**
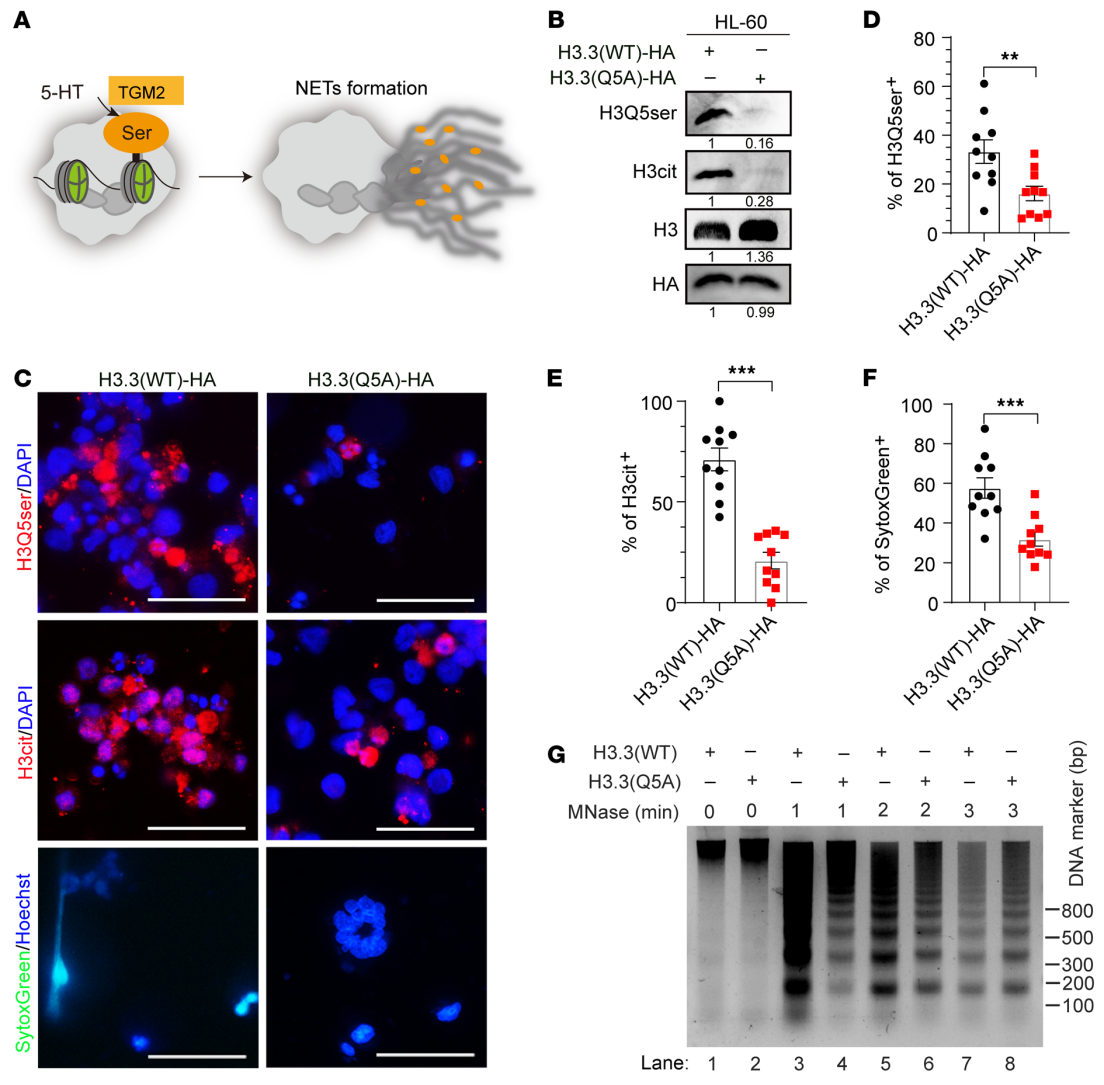
H3Q5ser modification promotes the formation of NETs. (**A**) Schematic diagram showing that 5-HT-mediated histone serotonylation promotes a decondensed chromatin state and facilitates the formation of NETs. (**B**) Immunoblot results show reduced levels of H3Q5ser and H3Cit modifications in H3.3(Q5A) mutant–transfected versus H3.3(WT)-transfected HL-60 granulocytes. (**C**–**F**) IF staining images (**C**) and quantification data (**D**–**F**) showing reductions in H3Q5ser (**D**) and NET formation in H3.3(Q5A) mutant–transfected HL-60 granulocytes, as reflected by decreased H3cit (**E**) and SytoxGreen (**F**) signals (*n* = 10 biological replicates per experiment). Scale bars: 50 μm (**C**). (**G**) MNase assay data revealed that chromatin in H3.3(Q5A)-transfected HL-60 granulocytes was less accessible than that in H3.3(WT)-transfected cells in response to calcium ionophore induction. **P < 0.01 and ***P < 0.001, by 2-tailed Student’s *t* test (**D**–**F**). Data are presented as the mean ± SEM.

To further corroborate the essential role of H3Q5ser on NET formation (Figure 4A), we induced overexpression of either HA-tagged histone H3.3(WT) or the H3.3(Q5A) mutant in HL-60 granulocytes (Figure 4B). As shown in Figure 4B, the Q5A mutation of H3.3 blocked the formation of H3Q5ser. Meanwhile, the H3cit deposition was markedly attenuated when H3Q5ser was inactivated. IF results further showed that the formation of NETs was significantly suppressed when the H3Q5ser modification was inhibited in H3.3(Q5A)-expressing HL-60 cells in comparison with H3.3(WT)-transfected counterparts (Figure 4, C–F). Furthermore, the MNase assay results demonstrated a less accessible chromatin structure in H3.3(Q5A)-transfected HL-60 cells compared with that in H3.3(WT)-transfected cells (Figure 4G). Altogether, these results showed that TGM2-mediated H3Q5ser facilitated NET formation.

### Genetic deletion or small-molecule inhibition of TGM2 suppresses NEPC liver metastasis.

**Figure 5:**
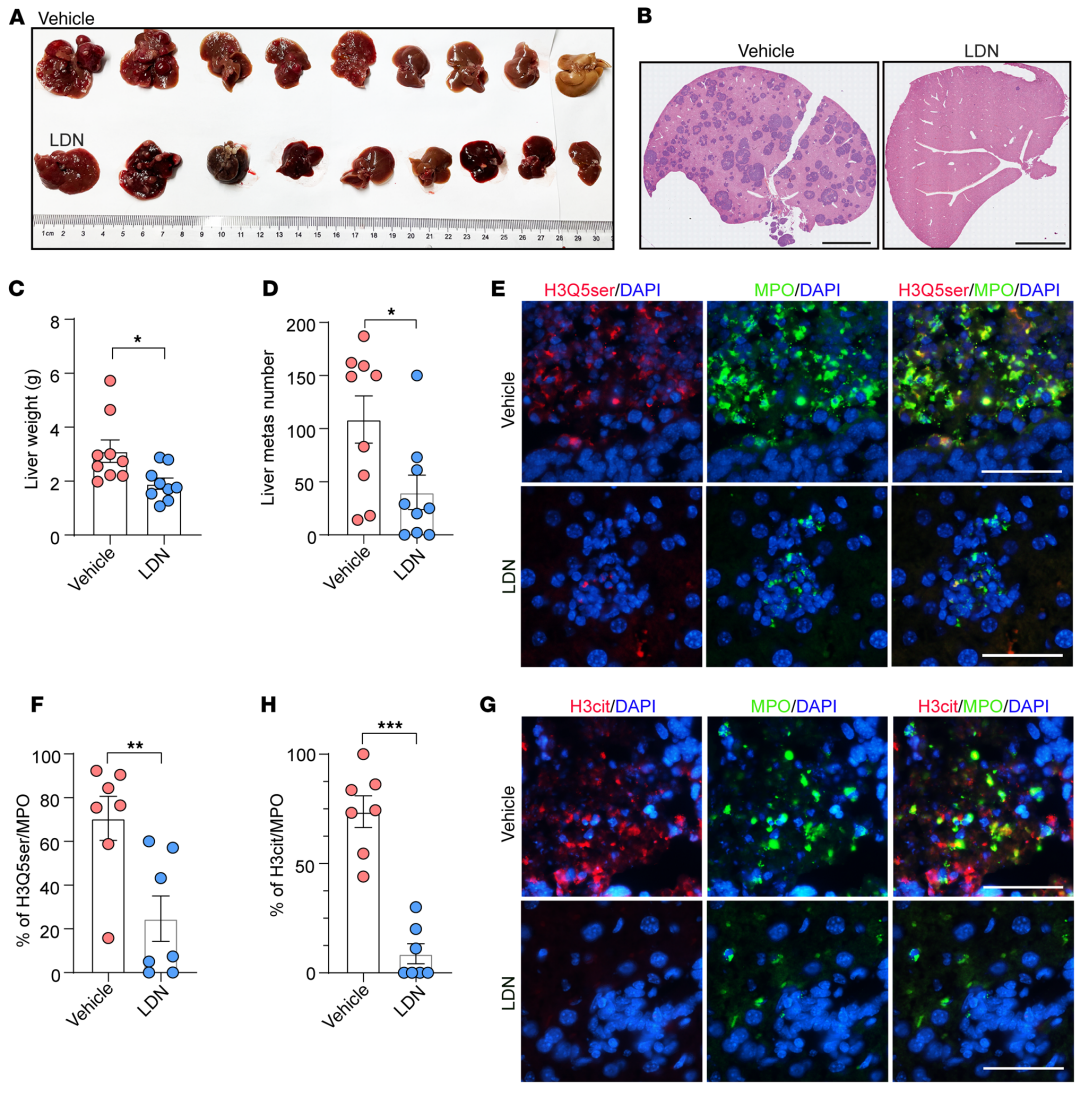
TGM2 inhibition abrogates NET formation and liver metastasis in NEPC mouse models. (**A**) The TGM2 inhibitor LDN suppressed the liver metastatic burden in mice inoculated by i.v. injection with *Rb1^Δ/Δ^* *Trp53^Δ/Δ^* NEPC organoids (*n* = 9 mice, each group). (**B**) H&E staining showing reduced liver metastatic foci in response to treatment with LDN. Scale bars: 4 mm. (**C** and **D**) Quantification of liver weights (**C**) and liver metastatic foci numbers (**D**) in *Rb1^Δ/Δ^* *Trp53^Δ/Δ^* organoid–inoculated mice upon treatment with vehicle or LDN (*n* = 9 mice, each group). (**E** and **F**) IF images (**E**) and quantification results (**F**) showing decreased histone serotonylation (H3Q5ser^+^) in hepatic neutrophils (MPO^+^) in *Rb1^Δ/Δ^* *Trp53^Δ/Δ^*–inoculated mice upon treatment with vehicle or LDN (*n* = 7 biological replicates). Scale bars: 50 μm. (**G** and **H**) IF images (**G**) and quantification (**H**) of neutrophil-derived (MPO^+^-derived) NETs (H3cit) in *Rb1^Δ/Δ^* *Trp53^Δ/Δ^*–inoculated mice in response to vehicle or LDN treatment (*n* = 7 biological replicates). Scale bars: 50 μm. *P < 0.05, **P < 0.01, and ***P < 0.001, by Mann-Whitney *U* test. Data are presented as the mean ± SEM.

We subsequently investigated the effect of TGM2 inhibition on NEPC liver metastasis. Syngeneic C57BL/6 mice were injected i.v. with *Rb1^Δ/Δ^* *Trp53^Δ/Δ^* NEPC organoids. The TGM2 inhibitor LDN (Figure 3A) was applied on post-inoculation day 4. We noticed a significant reduction in liver metastasis in LDN-treated mice (Figure 5, A–D). IF staining of liver sections from vehicle- and LDN-treated NEPC tumor–bearing mice revealed a significant decrease in H3Q5ser modifications in hepatic neutrophils upon LDN treatment (Figure 5, E and F). In accordance with repressed H3Q5ser levels, H3cit modification in hepatic neutrophils was simultaneously suppressed (Figure 5, G and H). To validate the role of TGM2 in driving liver metastasis in other NE cancer types, we treated the SCLC NCI-H82 cell–inoculated nude mice with the TGM2 inhibitor LDN. As shown in Supplemental Figure 5, A–C, we observed a significant attenuation of liver metastatic lesions in LDN-treated SCLC tumor–bearing mice compared with their vehicle-treated counterparts. Co-IF staining for H3Q5ser/MPO and H3cit/MPO revealed a significant reduction of H3Q5ser levels (Supplemental Figure 5, D and E) and NET production (Supplemental Figure 5, F and G) in liver sections upon LDN treatment in vivo.

On the other hand, we used WT and *Tgm2^–/–^* mice as xenograft recipients for *Rb1*^Δ/Δ^ *Trp53*^Δ/Δ^ NEPC organoids. We observed significant reductions in the number of liver metastasis foci (Supplemental Figure 6, A and B), NET formation (Supplemental Figure 6, C and D), and H3Q5ser deposition (Supplemental Figure 6, E and F) in the *Tgm2*-KO recipient mice. Therefore, our data demonstrate a crucial role of TGM2 in NEPC metastasis in the liver.

### TGM2 collaborates with PAD4 to orchestrate histone serotonylation and citrullination.

**Figure 6:**
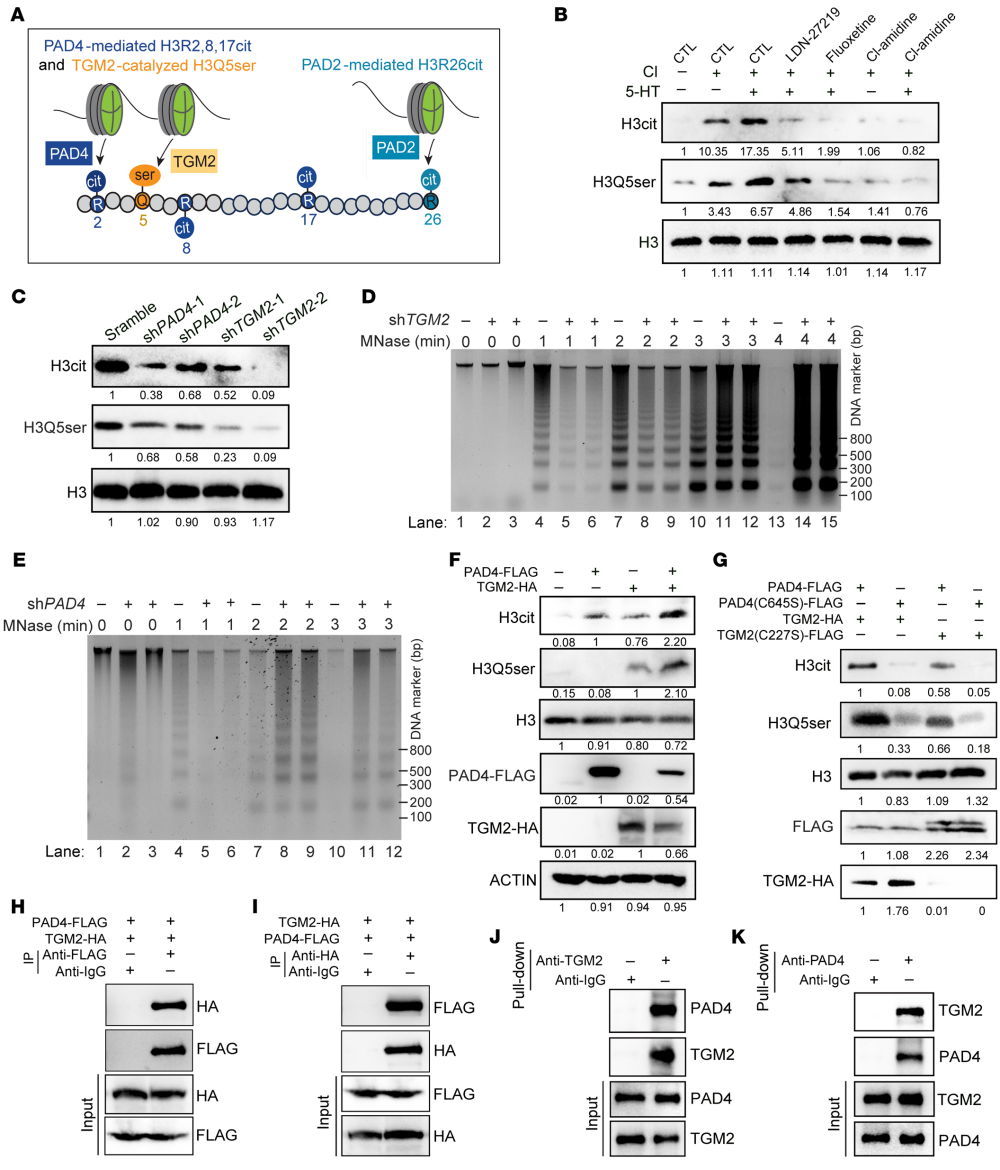
TGM2 collaborates with PAD4 to coordinate histone serotonylation and citrullination. (**A**) Schematic diagram showing proximal locations of TGM2-catalyzed H3Q5ser and PAD4-mediated H3R2,8,17cit and PAD2-promoted H3R26cit on histone H3. (**B**) Immunoblot assays revealed increased H3cit and H3Q5ser levels in HL-60–derived granulocytes upon 5-HT stimulation. (**C**) Immunoblot data revealed reductions in H3cit and H3Q5ser modifications in either sh*PAD4* or sh*TGM2*-HL-60 cells compared with scrambled shRNA–transfected cells. (**D** and **E**) The MNase assay data demonstrated that KD of either *TGM2* (**D**) or *PAD4* (**E**) in HL60 cells attenuated calcium ionophore–induced chromatin decondensation. (**F**) Dual expression of HA-tagged TGM2 and Flag-tagged PAD4 in HEK-293T cells showing elevated levels of both H3cit and H3Q5ser modifications. (**G**) Expression of the enzyme-dead mutant of TGM2-C277S and PAD4-C645S in HEK-293T cells abrogates their synergistic effect in enhancing H3cit and H3Q5ser depositions. (**H** and **I**) The co-IP assay showed an exogenous interaction between TGM2 and PAD4 by IP Flag-tagged PAD4 (**H**) and HA-tagged TGM2 (**I**) in HEK-293T cells. (**J** and **K**) Pulldown assay demonstrates the protein-protein association between TGM2 and PAD4 in vitro.

It is very interesting that H3Q5ser and H3cit showed the same trend of change during the formation of NETs in response to treatment with the H3Q5ser inhibitor or upon introduction of histone 3 mutations (Figure 3, B–G, Figure 4, B–F, and Figure 5, E–H). These findings, together with the approximate locations of these 2 modifications on the histone 3 backbone (Figure 6A), led us to explore the potential crosstalk between H3Q5ser and H3cit during the formation of NETs.

Next, we assessed the effect of inhibition or KD of the respective catalytic enzymes on these 2 histone modifications by immunoblotting. Consistent with the IF staining data shown in Figure 2, A, B, E, and F, addition of 5-HT markedly promoted the deposition of H3Q5ser and H3cit in HL-60 granulocytes (Figure 6B). Strikingly, inhibition of TGM2 by LDN led to a marked downregulation of both H3Q5ser and H3cit in HL-60 granulocytes (Figure 6B). Addition of Fluox resulted in a simultaneous inhibition of H3Q5ser and H3cit (Figure 6B). In contrast, treatment with thePAD4 inhibitor Cl-amidine also caused prominent decreases in H3cit and H3Q5ser levels (Figure 6B). To validate this finding, we knocked down PAD4 or TGM2 in HL-60 granulocytes and found that downregulation of PAD4 or TGM2 resulted in prominent reductions of both modifications (Figure 6C). Moreover, the MNase digestion assay showed that either TGM2 (Figure 6D) or PAD4 (Figure 6E) KD suppressed the decondensed chromatin state that is required for NET formation. Therefore, these data suggested a mutually enhanced effect between TGM2-catalyzed H3Q5ser and PAD4-mediated H3cit.

Furthermore, we used an alternative human cell line, HEK-293T, which barely express TGM2 and PAD4, as a clean system to conduct exogenous expression of HA-tagged TGM2 and FLAG-tagged PAD4 (6, 29). As shown in Figure 6F, coexpression of PAD4 and TGM2 in HEK-293T cells led to a synergistic effect in promoting the deposition of both H3cit and H3Q5ser. In addition, we introduced the enzyme-dead mutant of TGM2(C277S) or PAD4(C645S) into HEK-293T cells. We detected a reduction in H3cit when TGM2 was inactively mutated (Figure 6G). Meanwhile, we found that PAD4 inactivated mutation led to a H3Q5ser suppression (Figure 6G). Those data, together with the immunostaining results shown in Figures 2 and 3, indicated that H3Q5ser and H3cit are closely linked histone modifications.

To decipher the underlying mechanism of H3Q5ser and H3cit crosstalk, we first tested whether an interaction exists between their respective enzymes PAD4 and TGM2. We performed IPs of exogenous PAD4 and TGM2 in HEK-293T cells. As shown in Figure 6, H and I, we detected a physical association of PAD4 and TGM2 by co-IP. Protein interaction between endogenous PAD4 and TGM2 was also detected in HL-60 cells (Supplemental Figure 7, A and B). To further decipher the physical association between PAD4 and TGM2, we subcloned truncated mutations of these 2 proteins on the basis of their well-characterized protein domains (Supplemental Figure 7, C and D). The co-IP results revealed that the first immunoglobulin-like domain (D1) of PAD4 and the third carboxy-terminal barrel domain (D3) of TGM2 were required for their physical interaction (Supplemental Figure 7, E and F). Moreover, we noticed that addition of 5-HT further enhanced the binding between PAD4 and TGM2 (Supplemental Figure 7, G and H). Additionally, we performed a pull-down assay using purified PAD4 and TGM2 proteins and demonstrated a direct physical binding between these 2 enzymes (Figure 6, J and K). Together, these data revealed a functional interplay between PAD4 and TGM2 that acted to orchestrate histone serotonylation and citrullination.

### H3Q5ser and H3cit are mutually permissive modifications.

**Figure 7:**
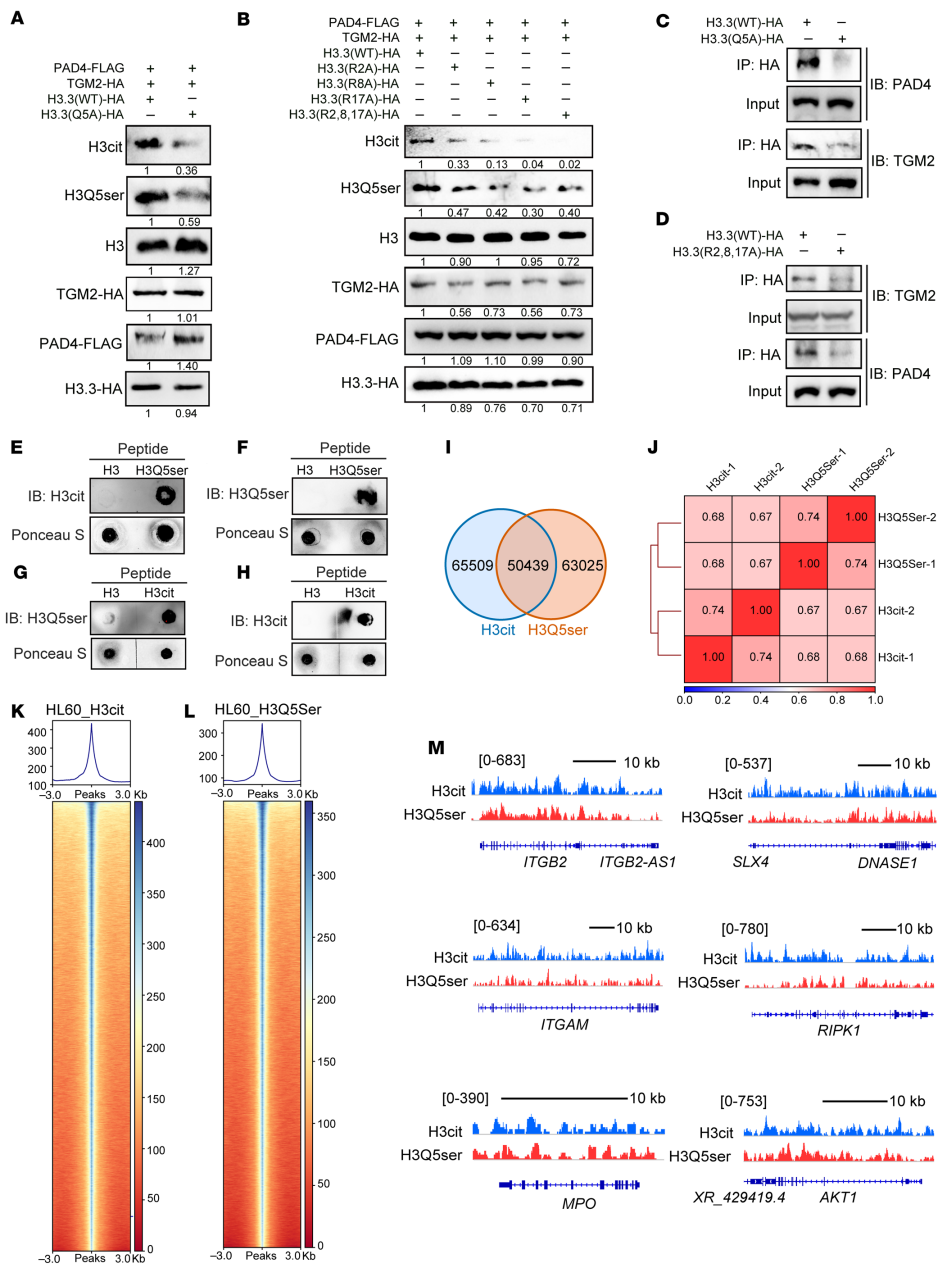
H3Q5ser and H3cit are mutually enhanced and share chromatin occupancy on a genome-wide scale. (**A**) Immunoblotting assay demonstrates that the H3.3(Q5A) mutant leads to deficient H3Q5ser modification and a reduced H3cit level. (**B**) Immunoblotting data show that either H3.3(R2A), H3.3(R8A), H3.3(R17A) mutants alone or in combination H3.3(R2,8,17A) result in deficient H3cit modification and a repressed H3Q5ser level. (**C** and **D**) Mutant H3.3(Q5A) (**C**) or H3.3(R2,8,17A) (**D**) attenuates the recruitment of each other’s epigenetic writer, as exemplified by deficient binding of either PAD4 (**C**) or TGM2 (**D**) to histone H3. (**E** and **F**) In vitro catalytic assay showing that H3Q5ser-modified peptides enhanced PAD4-mediated H3cit compared with the unmodified H3 peptide control. (**G** and **H**) In vitro catalytic assay revealing that H3cit-modified peptides promoted TGM2-mediated H3Q5ser compared with the unmodified H3 peptide control. (**I**) Venn diagrams show the overlapped H3cit and H3Q5ser peaks in HL-60 granulocytes from CUT&Tag sequencing data (2 independent experiments). (**J**) Heatmap showing the genome-wide Spearman’s correlation between the H3Q5ser and H3cit modifications (2 independent experiments). (**K** and **L**) Heatmaps of H3cit (**K**) and H3Q5ser (**L**) peaks are shown on a genome-wide scale in HL-60 granulocytes (2 independent experiments). (**M**) CUT&Tag profiles of co-occupied NET-related genes including *ITGB2*, *DNASE1*, *ITGAM*, *RIPK1*, *MPO*, and *AKT1* of H3cit and H3Q5ser modifications in HL-60 granulocytes (2 independent experiments).

To directly examine the putative role of H3Q5ser on H3cit modification, we transduced either H3.3(WT) or H3.3(Q5A) into HEK-293T cells. As expected, the mutation of the fifth glutamine residue of H3.3 to the alanine residue prevented serotonylation on H3 (Figure 6A and Figure 7A). This downregulation of histone serotonylation led to a reduction of H3cit levels (Figure 7A). In addition, we generated the R2A, R8A, R17A, and R2, 8, 17A combinatorial mutations of H3 to eliminate citrullination at these sites (Figure 6A). As shown in Figure 7B, transfection of these mutations decreased the H3cit mark in concordance with a reduction of H3Q5ser deposition. We investigated whether the H3.3(Q5A) or H3.3(R2,8,17A) mutant, which blocks H3Q5ser or H3cit modification, affects the recruitment of each other’s epigenetic writer. Interestingly, we found that the H3.3(Q5A) mutation impeded the binding of both PAD4 and TGM2 to H3 (Figure 7C). Similarly, we detected a deficient recruitment of both PAD4 and TGM2 to histone when the H3.3(R2,8,17A) mutant was introduced (Figure 7D). These data suggest that H3cit and H3Q5ser are mutually permissive histone modifications.

A recent study reported that PAD2, another PAD family enzyme, promotes PCa progression and castration resistance via histone H3 citrullination at the 26th arginine (R26) residue (H3R26cit) (30) (Figure 6A). We asked whether PAD2-mediated H3R26cit affects the modulation of H3Q5ser and the formation of NETs. A PAD2 inhibitor, AFM-30a (AFM), was used to treat murine BMDNs in the presence of 5-HT. PAD2 inhibition did not significantly affect 5-HT–induced NET production (Supplemental Figure 8, A and B). To further assess the putative role of H3R26cit in H3Q5ser modification, we transfected HEK-293T cells with H3.3(WT) or H3.3(R26A) constructs. As shown in Supplemental Figure 8C, the mutation of H3.3(R26A) did not affect H3Q5ser modification. Moreover, co-IP results revealed that PAD2 was not physically associated with TGM2 (Supplemental Figure 8, D and E). These results suggest that PAD2-catalyzed H3R26cit is not involved in H3Q5ser modification or NET formation.

To directly analyze the effect of H3Q5ser modification on the addition of H3cit mark and vice versa, we performed in vitro histone serotonylation and citrullination assays. The i vitro catalytic reaction using purified PAD4 on H3 and H3Q5ser peptides showed that serotonylation on H3Q5 substantially enhanced the PAD4-mediated H3cit deposition compared with unmodified H3 (Figure 7, E and F). Concordantly, citrullination on H3 also strengthened the TGM2-mediated H3Q5ser signal compared with unmodified H3 (Figure 7, G and H). These results further support a mutual promoting effect between the formation of H3cit and H3Q5ser modifications.

To determine the relationship between H3Q5ser and H3cit chromatin occupancy, we conducted CUT&Tag experiments and compared the anti-H3cit and anti-H3Q5ser antibody immunoprecipitated DNA sequences. As shown in Figure 7, I–L, genome-wide localization analysis of HL-60 cells revealed a substantial overlap between H3Q5ser-occupied and H3cit-marked genomic regions. Notably, expression of NET formation–related genes, including *ITGB2*, *DNASE1*, *ITGAM*, *RIPK1*, *MPO*, and *AKT1*, was detected in the H3Q5ser and H3cit co-occupied gene locus (Figure 7M). Together, our results suggest that TGM2-catalyzed H3Q5ser and PAD4-mediated H3cit are closely linked and share a substantial overlap of regulatory genomic regions on a genome-wide scale.

### Targeting SERT by Fluox blocks liver metastasis of NE cancers.

**Figure 8:**
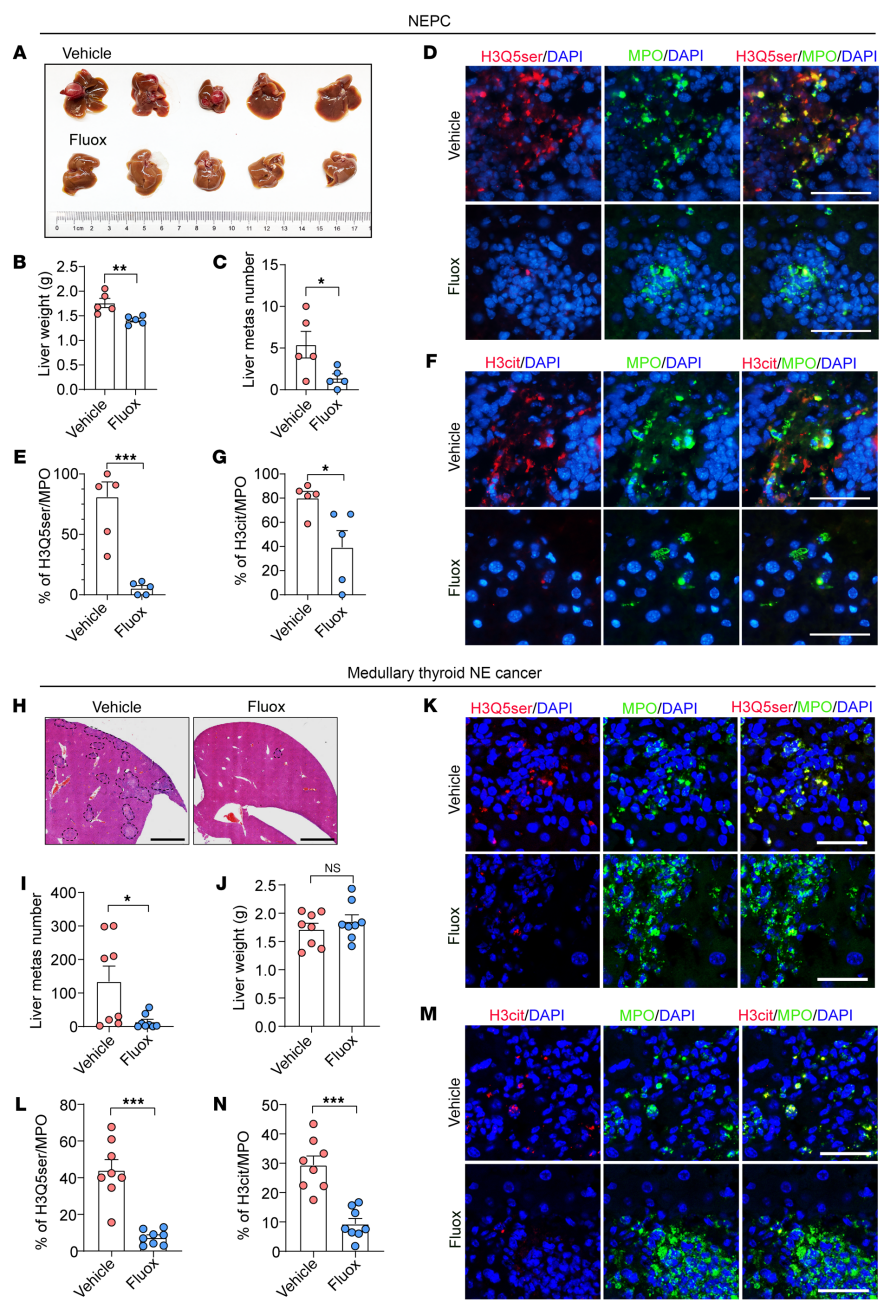
The SERT inhibitor Fluox represses NET formation and liver metastasis in NE cancers. (**A**–**C**) The SERT inhibitor Fluox suppressed liver metastasis (**A**) in mice i.v. inoculated with *Rb1^Δ/Δ^* *Trp53^Δ/Δ^*, as exemplified by decreased liver weights (**B**) and number of metastasis foci (**C**) compared with vehicle-treated mice (*n* = 5 mice per group). (**D** and **E**) IF staining images (**D**) and quantification results (**E**) showing decreased histone serotonylation (H3Q5ser^+^) in hepatic neutrophils (MPO^+^) in *Rb1^Δ/Δ^* *Trp53^Δ/Δ^*–inoculated mice upon treatment with Fluox as compared with vehicle treatment (*n* = 5 mice per group). Scale bars: 1.5 mm. (**F** and **G**) IF images (**F**) and quantification results (**G**) of neutrophil-derived (MPO^+^-derived) NETs (H3Cit) in *Rb1^Δ/Δ^* *Trp53^Δ/Δ^*–inoculated mice in response to vehicle or Fluox treatment (*n* = 5 mice per group). Scale bars: 50 μm. (**H**–**J**) H&E-stained images (**H**) and quantification results showing decreased metastasis foci numbers (**I**) in i.v. TT cell–inoculated mice following treatment with Fluox compared with their vehicle-treated counterparts (*n* = 8 mice per group). Scale bars: 1 mm. (**K** and **L**) IF staining images (**K**) and quantification results (**L**) showing decreased H3Q5ser levels in MPO+ neutrophils in the liver sections of TT cell-inoculated mice upon treatment with Fluox versus treatment with vehicle (*n* = 8 mice per group). Scale bars: 50 μm. (**M** and **N**) IF images (**M**) and quantification (**N**) of NETs in TT-inoculated mice in response to vehicle or Fluox treatment (*n* = 8 mice per group). Scale bars: 50 μm. *P < 0.05, **P < 0.01, and ***P < 0.001, by 2-tailed Student’s *t* test (**B**, **C**, **E**, **J**, **L**, and **N**) and Mann-Whitney *U* test (**G** and **I**). Data are presented as the mean ± SEM.

To investigate the clinical application of our findings, we applied the FDA-approved SERT inhibitor Fluox to *Rb1*^Δ/Δ^ *Trp53*^Δ/Δ^ NEPC organoid–injected C57BL/6 mice. As shown in Figure 8, A–C, treatment with Fluox effectively inhibited the liver metastasis burden (Figure 8A). Mice that received Fluox therapy showed significant reductions in liver weight (Figure 8B) and number of metastases (Figure 8C). Both H3Q5ser and H3cit staining intensities were decreased in Fluox-treated liver metastatic lesions, suggesting that inhibition of 5-HT uptake suppressed H3Q5ser (Figure 8, D and E). MPO- and H3cit-costained NETs were significantly less apparent in the liver metastasis sections from the Fluox-treated group (Figure 8, F and G). To further explore whether these findings identified in NEPC could also be applied to other NE cancers, we performed additional experiments using SCLC NCI-H82 cells (Supplemental Figure 9) and medullary thyroid cancer TT cells (Figure 8, H–N). In line with the results for the NEPC mouse model, Fluox treatment significantly repressed NCI-H82 (Supplemental Figure 9, A–C) and TT (Figure 8, H–J) liver metastasis in vivo. IF images showed concomitant staining reductions of both H3Q5ser and H3cit in MPO^+^ neutrophils in metastasis–containing liver sections from SCLC (Supplemental Figure 9, D–G) and medullary thyroid cancer (Figure 8, K–N) models. These data indicate a potential therapeutic value of the FDA-approved SERT inhibitor Fluox in blocking NET formation and preventing liver metastasis of pleiotropic NE cancers.

## Discussion

Here, we demonstrate that 5-HT, derived from NE cancer cells, promoted liver metastasis through crosstalk with neutrophils. 5-HT was taken up by accumulating neutrophils in the liver via SERT and induced TGM2-mediated histone serotonylation. In concert with PAD4-catalyzed histone citrullination, histone serotonylation led to a decondensed chromatin status in neutrophils, triggering the formation of NETs, a process that is also called NETosis. NETs then recruited and trapped cancer cells, leading to cancer metastasis. Therapeutically, inhibition of 5-HT intake via the SERT inhibitor Fluox could effectively abrogate liver metastasis in preclinical mouse models. Therefore, in addition to the well-known role of 5-HT in the nervous system, our findings show that the neurotransmitter 5-HT acted as a positive regulator in facilitating a prometastatic microenvironment in NE cancers.

We elucidate the mechanism by which epigenetic modification of histone serotonylation is required to promote NET formation in a 5-HT–dependent manner. Serotonylation has been implicated in different substrate proteins with biological functions. Our recent work reported that serotonylation of GAPDH promotes CD8^+^ T cell activation (7). In the current study, we uncovered a function of 5-HT–induced and TGM2-catalyzed histone serotonylation in neutrophils, a subtype of innate immune cells. This conclusion is supported by multiple lines of evidence including TGM2 or SERT inhibition, TGM2 mutation or KO, and H3 (Q5A) mutation. We included 3 NE cancer models — NEPC, SCLC, and thyroid medullary cancer — in this study. Of note, CNS cancers such as glioblastoma or nerve-infiltrating cancers also produce high levels of 5-HT (31, 32). Whether a similar mechanism of 5-HT–induced histone serotonylation in neutrophils and NET formation is also involved in the progression of these malignancies would be interesting to explore.

We identified a reciprocally reinforcing effect between the histone modifications of H3Q5ser and H3cit and a previously unreported crosstalk between their respective writers of TGM2 and PAD4. Coordinated histone modifications have been demonstrated to be essential for regulating chromatin accessibility and transcriptional activity (12). For example, it has been shown that Nsd1-mediated H3K36me2 colocalizes with PRC2-mediated H3K27me2 and that histone methyltransferase Nsd1 modulates the polycomb-repressive complex 2 (PRC2) function to demarcate H3K27me2 and H3K27me3 for transcriptional regulation (33, 34). In the current study, we reveal another collaboration between H3Q5ser and H3cit based on biochemical experiments including TGM2 and PAD4 KD or an enzymatically dead mutation; a mutation with H3Q5ser (H3Q5A) or H3cit (H3R2,8,17A) deficiency; as well as IP, pulldown, CUT&Tag-Seq, and in vitro histone serotonylation and citrullination assays using multiple cell lines in both murine and human settings. Furthermore, using *Tgm2*-KO mice and orthotopically and i.v. inoculated preclinical models of NEPC, SCLC, and thyroid medullary cancer, our results imply that the crosstalk between H3Q5ser and H3cit was of functional importance in NET formation and cancer metastasis. Given that H3Q5ser plays a vital role in neuronal differentiation and olfactory sensory processing and that PAD4-expressing neurons release citrullinated proteins in Alzheimer’s disease, the crosstalk between H3Q5ser and H3cit may also be relevant to nervous system functions and awaits future study (6, 35, 36).

In summary, we found that histone serotonylation and histone citrullination are 2 closely linked types of histone modifications in neutrophils that are orchestrated to regulate chromatin decondensation, drive the formation of NETs, and promote liver metastasis. 5-HT–driven NET production highlights a targetable neurotransmitter/immune axis in driving liver metastasis of NE cancers. We found that targeting the key enzyme of the 5-HT biosynthesis enzyme TPH1, the serotonylation enzyme TGM2, or the 5-HT transporter SERT effectively inhibited NET formation and liver metastasis in NEPC. Among them, the SERT inhibitor Fluox is an FDA-approved antidepressant drug with good biosafety and bioavailability (2), suggesting a promising translational value in drug repurposing. Interestingly, a recent study showed that Fluox inhibits NEPC growth by suppressing the cell-autonomous role of 5-HT in cancer cells (37). Different from this related work, our study as mainly focused on NEPC liver metastasis and demonstrated that Fluox blocked H3Q5ser and NET formation by interfering with the effects of 5-HT on neutrophils. Therefore, Fluox acted as a potential drug in harnessing cancer cell growth and distant metastasis of advanced prostate cancer via different mechanisms.

Aside from NEPC, a high incidence of liver metastasis is commonly seen in pleiotropic NE malignancies (32, 38, 39). In addition to the 3 types of NE cancer models — including NEPC, SCLC, and medullary thyroid cancer — tested in this study, the molecular mechanism and therapeutic strategy we propose is informative for a wide range of NE malignancies with such a propensity for liver metastasis.

## Methods

### Sex as a biological variable.

Our study of prostate cancer involved the use of male mice, as it is a male-exclusive disease. For experiments on SCLC and medullary thyroid cancer, male mice were chosen because of their lower phenotypic variability. The relevance of these findings to female mice is unknown.

### Mice.

All animals used in this study were housed under humidity- and temperature-controlled conditions with a regular light-and-dark cycle in the specific pathogen–free facilities at Renji Hospital. Transgenic mice with prostate-specific dual depletion of *Rb1* and *Trp53* (*Probasin-Cre*^+^ *Rb1^fl/fl^* *Trp53^fl/fl^* [*Rb1*^Δ/Δ^ *Trp53*^Δ/Δ^] mice) were obtained from The Jackson Laboratory. The conventional *Tgm2*-KO (*Tgm2^–/–^*) mice were commercially available from Cyagen Biosciences. The 6- to 8-week-old C57BL/6 mice and athymic nu/nu nude mice were purchased from Shanghai SLAC Laboratory Animal Company.

### Cell lines.

The human granulocyte cell line HL-60, the human SCLC cell line NCI-H82, the medullary thyroid carcinoma cell line TT, and HEK-293T cells were purchased from the Cell Bank of the Chinese Academy of Sciences. *Rb1*^Δ/Δ^ *Trp53*^Δ/Δ^ NEPC murine prostate cancer organoids were established and cultured as previously reported (18). All cells used in this study were validated by short tandem repeat (STR) analyses. The cells were maintained in incubators at 37°C with 5% CO_2_. HL-60 cells were cultured in IMDM (Gibco, Thermo Fisher Scientific) with 20% FBS. NCI-H82 cells were cultured in RPMI-1640 (Gibco, Thermo Fisher Scientific) with 10% FBS. TT and HEK-293T cells were cultured in DMEM (Gibco, Thermo Fisher Scientific) with 10% FBS.

### Immune analysis.

In detail, the SU2C prostate cancer dataset was downloaded, and the gene expression matrix of liver metastases was obtained. Next, we used the quanTIseq function in Multi-omics Immuno-Oncology Biological Research (IOBR) R package (https://github.com/IOBR/IOBR, version: 4.3.2) to analyze the infiltration of immune cells in liver metastatic biospecimens from 6 patients (*n* = 6). The enrichment of 10 immune cell subsets in liver metastasis was generated by the quanTIseq function. Subsequently, the ggplot function in the ggplot2 (3.5.1) package was used to generate the pie chart (Figure 1A) revealing fractions of the 10 subsets of infiltrating immune cells, including B cells, DCs, M1 macrophages, M2 macrophages, monocytes, neutrophils, NK cells, CD4^+^ and CD8^+^ T cells, and Tregs. In addition, neutrophil frequencies in adrenal, bone, liver, lymph nodes, and other visceral metastatic sites were analyzed using the quanTIseq function to generate the violin plot (vioplot R package 0.5.0) in Figure 1B.

### Tumor xenograft experiments.

The WT C57BL/6 mice (6–8 weeks old) were i.v. injected with 2,000 *Rb1^Δ/Δ^* *Trp53^Δ/Δ^* organoid spheres. For DNase I treatment, mice were i.p. injected with DNase I at the dosage of 50 μg per mouse every other day, starting from day 4 after inoculation, until the mice were sacrificed on day 30. For LDN-27219 treatment, mice were i.p. injected daily with 15 mg/kg LDN-27219 from day 4 after inoculation, and daily administration was continued until day 30. For the Fluox treatment, 10 mg/kg Fluox was administered via oral gavage every other day from day 4 after inoculation until the mice were sacrificed on day 30. Six-week-old nude male mice were i.v. injected with 5 × 10^5^ human TT cells or 5 × 10^5^ human NCI-H82 cells. The nude mice were i.p. injected with 15 mg/kg LDN-27219 every other day, starting from day 4 after inoculation until day 30 when the tumor-bearing mice were sacrificed. Fluox (10 mg/kg per mouse) was administrated to the *Rb1^Δ/Δ^* *Trp53^Δ/Δ^* organoid–inoculated mice via oral gavage from day 4 to day 30 after inoculation. Fluox was dissolved in the drinking water at a concentration of 20 mg/L.

### In vitro NET production assay.

To assess NET formation, coverslips were put into 24-well plates in advance, and neutrophils were seeded in RPMI-1640 or CM at 2.5 × 10^5^ cells per well. 5-HT (100 μM), TGM2 inhibitor (LDN-27219, 10 μM), SERT inhibitor (Fluox, 10 μM), PAD4 inhibitor (Cl-amidine, 100 μM), or CM was added to stimulate neutrophils for 12–16 hours. SytoxGreen (Beyotime, C1070M) and Hoechst (Invitrogen, Thermo Fisher Scientific, H3570) stainings were performed immediately after these stimulated neutrophils were collected. Anti-H3cit, anti-H3Q5ser, and anti-MPO immunostainings were performed after cells were fixed with 4% paraformaldehyde (PFA). Images were captured using the Confocal Microscopy Cell Observer (Zeiss). H3cit^+^, H3Q5ser^+^, and MPO^+^ cells were quantified using ImageJ software (NIH).

### Statistics.

Data were statistically analyzed using GraphPad Prism 8.4.3 (GraphPad Software). Data are presented as the mean ± SEM. Kaplan-Meier analysis was performed to analyze animal survival, and a log-rank test was used for comparisons among groups. The correlation between 2 modification levels was analyzed via Spearman’s correlation test. A 2-tailed Student’s *t* test or Mann-Whitney *U* test was used to determine significance in 2-group experiments. One-way ANOVAs or Kruskal-Wallis tests were used to determine the significance in multiple-group experiments. A *P* value of less than 0.05 was considered statistically significant.

### Study approval.

All animal experiments were approved by the IACUC of Renji Hospital, Shanghai Jiao Tong University School of Medicine. The collection and utilization of patient clinical samples including primary prostate tumors and liver metastases were approved by the ethics committee of Renji Hospital, Shanghai Jiao Tong University School of Medicine. Informed consent was obtained from all patients and donors.

### Data availability.

The CUT&Tag data in this study have been deposited in the database of the China National Center for Bioinformation (accession number HRA007336). All antibodies and reagents used in this study are presented in Supplemental Table 1. The RT-PCR primer sequences are available in Supplemental Table 2. The shRNA sequences used in this study are shown in Supplemental Table 3.Values for all data points are presented in the Supporting Data Values file. Other methods were available in the Supplemental Methods.

## Author contributions

KYL and YCZ performed most of the experiments and contributed equally to this work. GYD, XYC, and CXZ conducted bioinformatics analyses and CUT&Tag experiments. LLX and NJ assisted with data collection and biochemical assays. PHX, HFZ, JMW, CPC, and DW helped with cell culturing and plasmid construction. YYL, LYJ, and YJS assisted with mouse breeding. WX and JHP provided clinical samples. WQG, ZGZ, SHJ, and PCZ provide important discussions and technical help and assisted with manuscript preparation. HHZ, KZ, and KYL wrote the manuscript. HHZ conceived this study. HHZ and KZ acquired funding and supervised this study.

## Supplementary Material

Supplemental data

Unedited blot and gel images

Supporting data values

## Version 1 02/04/2025

In-Press Preview

## Version 2 04/15/2025

Electronic publication
