# Supplementary material for: 5-HT orchestrates histone serotonylation and citrullination to drive neutrophil extracellular traps and liver metastasis
Source: J Clin Invest. 2025 Feb 4;135(8):e183544. doi: 10.1172/JCI183544 (PMC11996869; doi:10.1172/JCI183544)
Supplement: Supplemental data [file jci-135-183544-s275.pdf]

Supplemental Figures and figure legends

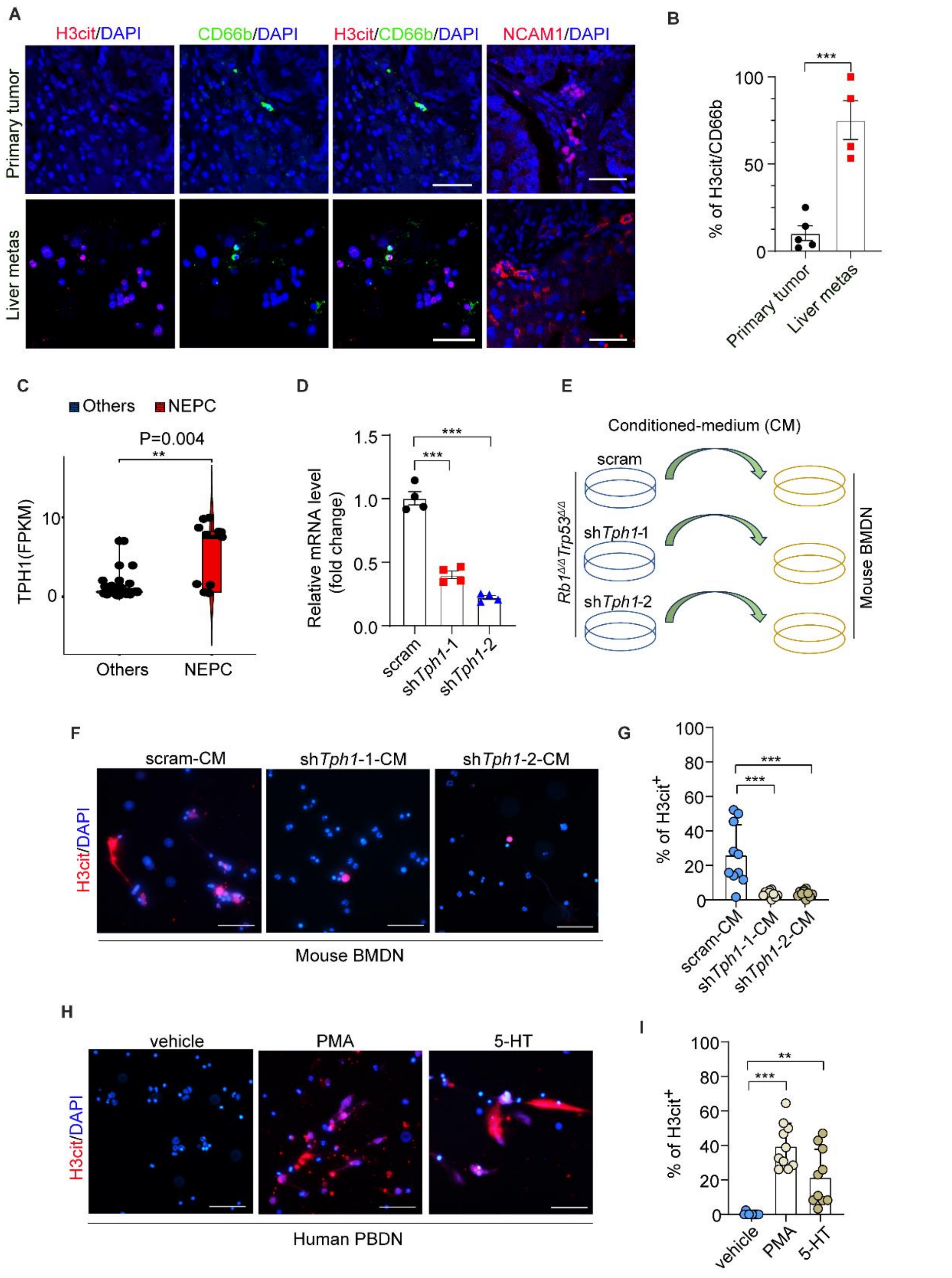

Supplemental Figure 1. TPH1-mediated 5-HT biosynthesis is required for NETs formation.

(A-B) IF images **(A)** and quantification **(B)** of NETs formation (H3cit$^+$) in CD66b$^+$ neutrophils in human primary NEPC (n = 5 patients) and liver metastases (n = 4 patients)

samples, showing that NETs were enriched in liver metastasis of NEPC patients. IF staining against NCAM1 was conducted to mark neuroendocrine tumor cells. Scale bars = 50 μm. Data in **(B)** were presented as mean ± SEM, and the two-tailed student's *t*-test was applied for statistics. ***P < 0.001.

(C) Analysis on the Beltran PCa dataset (1) demonstrates a significantly higher expression level of TPH1 in NEPC patients than that in other PCa subtypes (NEPC, n = 13; Others, n = 36). Data were presented as mean ± SEM, and the two-tailed student's *t*-test was applied for statistics. **P < 0.01.

(D) RT-qPCR confirms the knockdown efficiency of sh*Tph1* in *Rb1*$^{\Delta/\Delta}$*Trp53*$^{\Delta/\Delta}$ organoids. Data were presented as mean ± SEM, and the One-way ANOVA test was applied for statistics (n = 4 biological replicates). ***P < 0.001.

(E) A schematic chart showing the collection of conditional media (CM) from scramble and sh*Tph1*-infected *Rb1*$^{\Delta/\Delta}$*Trp53*$^{\Delta/\Delta}$ organoids and treatment on murine bone-marrow-derived neutrophils (BMDNs) with these CMs.

(F-G) IF images **(F)** and quantification results **(G)** on H3cit^{+} murine BMDNs upon CMs from scramble and sh*Tph1*-infected *Rb1*$^{\Delta/\Delta}$*Trp53*$^{\Delta/\Delta}$ organoids. Scale bars = 50 μm. Data were presented as mean ± SEM, and the One-way ANOVA followed by Dunnett's tests were applied for statistics (n = 10 biological replicates). ***P < 0.001.

(H-I) IF images **(H)** and quantification data **(I)** on H3cit^{+} human peripheral blood-derived neutrophils (PBDNs) upon 5-HT and vehicle control (n = 10 biological replicates). Scale bars = 50 μm. Data were mean ± SEM, **P < 0.01 and ***P < 0.001 were assessed using a Kruskal-Wallis test followed by a Dunnett's test in **I**.

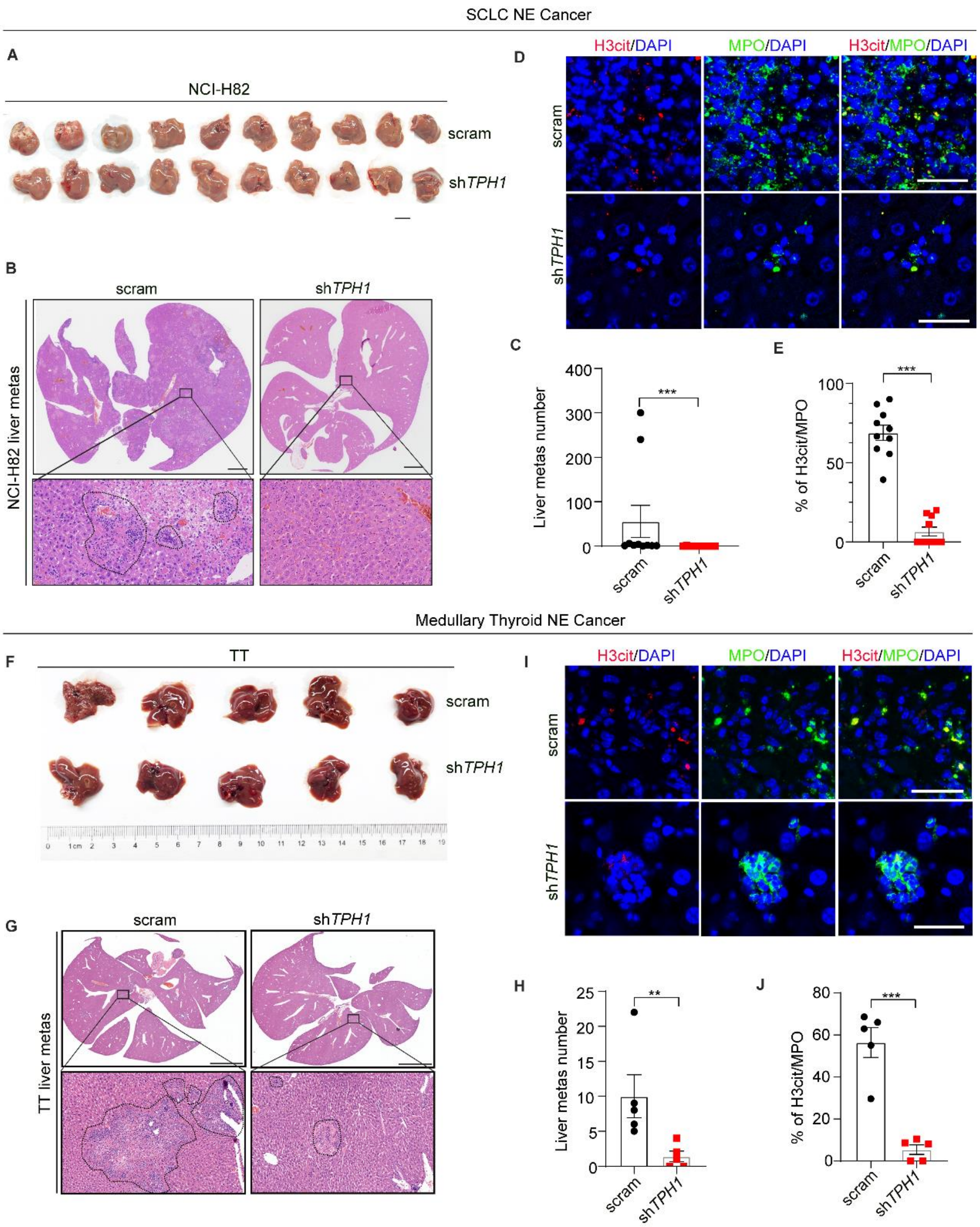

Supplemental Figure 2. Knockdown TPH1 attenuates liver metastasis and NETs formation in SCLC and medullary thyroid cancers.

(A-C) In vivo experiments demonstrating a reduction of liver metastatic burden **(A)** in sh*TPH1* infected NCI-H82 cells-implanted mice as compared to scramble control shRNA via intravenous injection (n = 10 mice, each group). H&E staining **(B)** and

quantification data **(C)** validate significantly decreased liver metastatic foci numbers in *TPH1*-knockdown NCI-H82-inoculated mice. Scale bar in **(A)** = 1 cm. In H&E staining images, scale bars = 2 mm.

(D-E) IF images on liver sections **(D)** and quantification results **(E)** revealing a significant decline in NETs formation in *TPH1*-knockdown NCI-H82 cells-inoculated mice (n = 10 mice, each group).

(F-H) In vivo experiments showing reduced liver metastatic foci **(F)** in sh*TPH1* infected TT medullary thyroid cells-implanted mice as compared to scramble control shRNA via intravenous injection (n = 5 mice, each group). H&E staining images **(G)** and quantification data **(H)** showing a significant reduction in liver metastasis foci number in sh*TPH1*-TT cells-inoculated nude mice. In H&E staining images, scale bars = 4 mm.

(I-J) IF images on liver sections **(I)** and quantification data **(J)** revealing a significant decline in NETs formation in *TPH1*-knockdown TT cells-inoculated mice (n = 5 mice, each group).

For **(C)**, **(E)**, and **(H)**, data were mean ± SEM and were assessed using Mann-Whitney tests. Data in **(J)** were mean ± SEM, and statistics were assessed using the two-tailed student's *t*-test. **P < 0.01, ***P < 0.001.

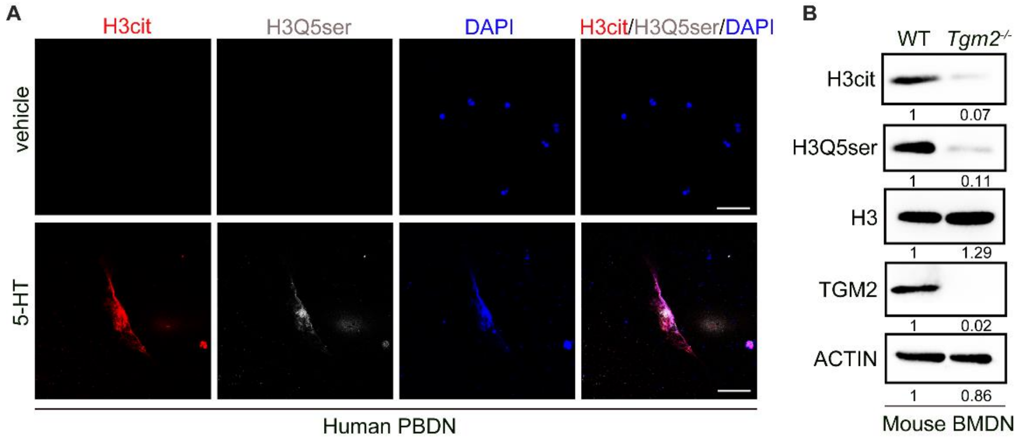

Supplemental Figure 3. 5-HT/TGM2 signaling promotes H3cit modification and NETs formation.

(A) IF images showing concomitantly upregulated H3cit and H3Q5ser signals in human PBDNs upon 5-HT addition as compared to vehicle control.

(B) Immunoblotting assays demonstrate decreased H3cit and H3Q5ser levels in *Tgm2*$^{-/-}$ mice-derived BMDNs in contrast to WT mice-derived counterparts.

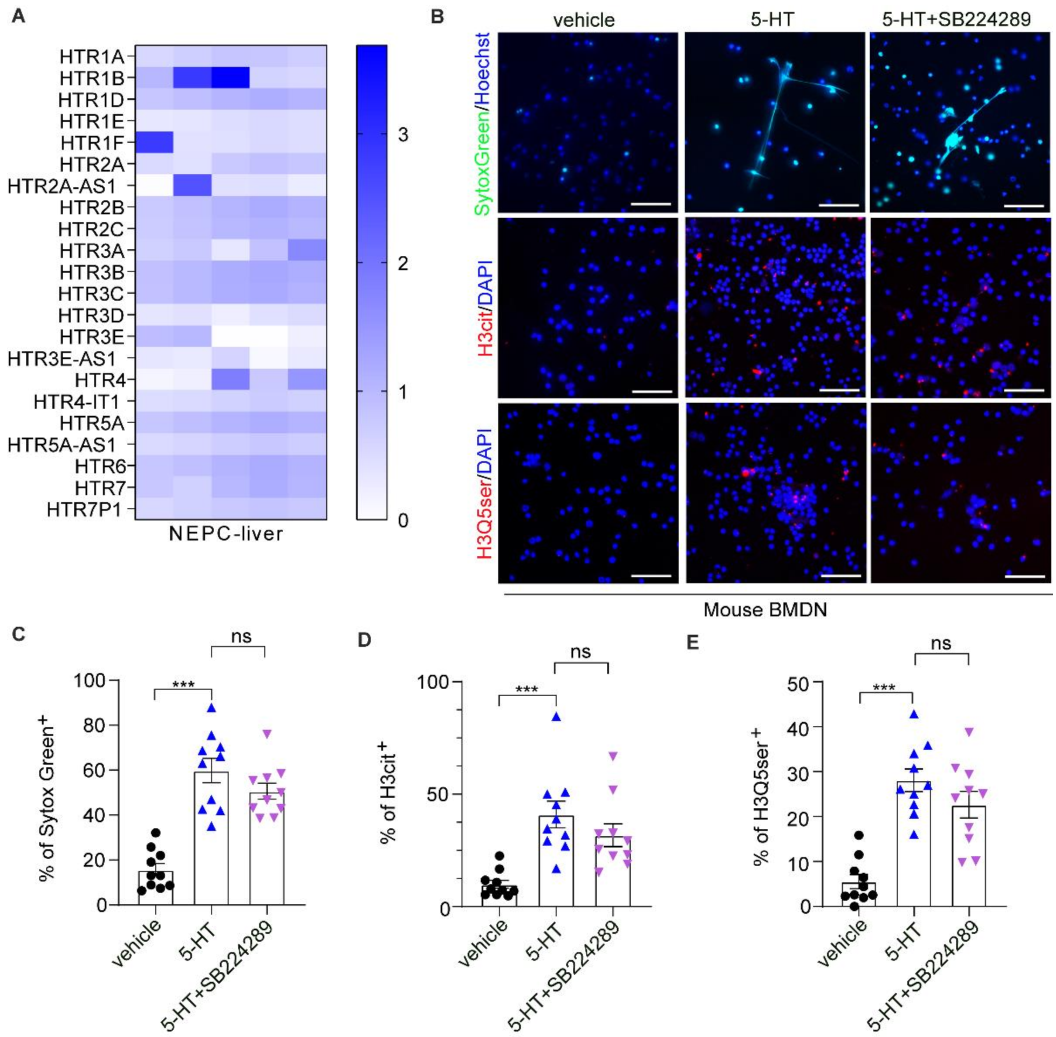

Supplemental Figure 4. HTR1b inhibition does not affect NETs formation and H3Q5ser deposition.

(A) HTR expression in NEPC liver metastasis in Beltran PCa dataset (1).

(B-E) IF images **(B)** and quantification data **(C-E)** reveal that HTR1b inhibitor SB-224289 does not affect 5-HT-induced NETs formation, as exemplified by SytoxGreen signal **(C)** and H3cit **(D)** and H3Q5ser **(E)** modifications (n = 10 biological replicates per experiment). Scale bars = 50 μm. For **(C)** and **(E)**, data were mean ± SEM and were assessed using the One-way ANOVA followed by the Tukey's test. Data in **(D)** were mean ± SEM, and statistics were assessed using a Kruskal-Wallis test followed by Dunnett's test. ***P < 0.001. ns = no significance.

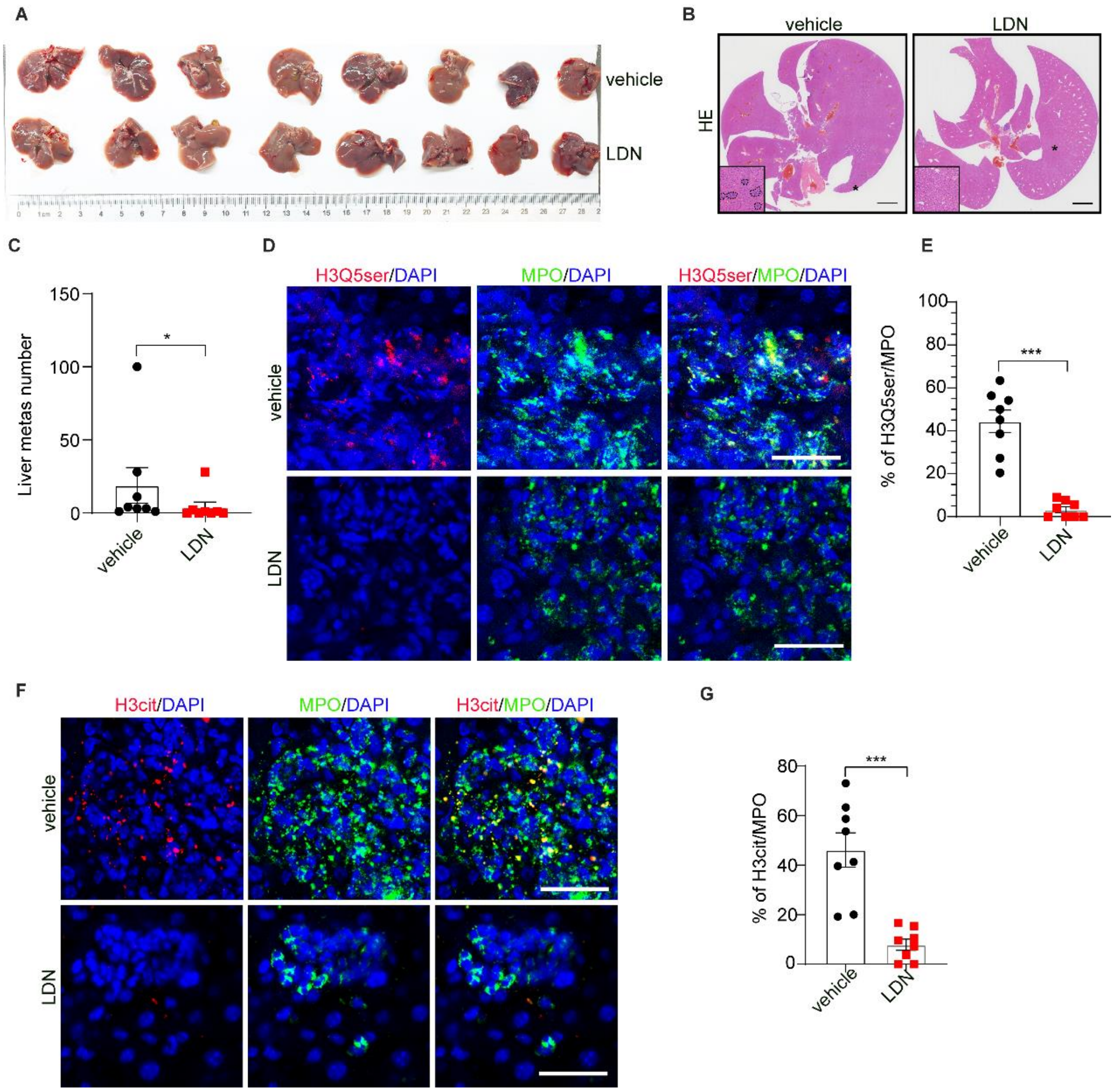

Supplemental Figure 5. TGM2 inhibition attenuates NETs formation and liver metastasis in NCI-H82 SCLC xenograft mice.

(A-C) In vivo experiments demonstrating a reduction of liver metastatic burden **(A)** in TGM2 inhibitor LDN-27219 (LDN)-treated NCI-H82 SCLC-implanted mice as compared to vehicle via intravenous injection (n = 8 mice, each group). H&E staining **(B)** and quantification data **(C)** validate significantly decreased liver metastatic foci numbers in LDN-treated NCI-H82-inoculated mice. In H&E staining images, scale bars = 2 mm.

(D-E) IF images **(D)** and quantification results **(E)** on liver sections showing decreased

H3Q5ser deposition after LDN treatment in NCI-H82 cells-inoculated nude mice (n = 8 mice). Scale bars = 50 μm.

(F-G) IF images **(F)** and quantification data **(G)** on liver sections showing a reduced NETs formation in neutrophils upon LDN treatment in NCI-H82 cells-inoculated nude mice (n = 8 mice).

Data in **(C)** and **(E)** were presented as mean ± SEM, *P < 0.05 and ***P < 0.001 were assessed using Mann-Whitney tests. Data in **(G)** are presented as mean ± SEM, ***P < 0.001 was assessed using the two-tailed student's *t*-tests.

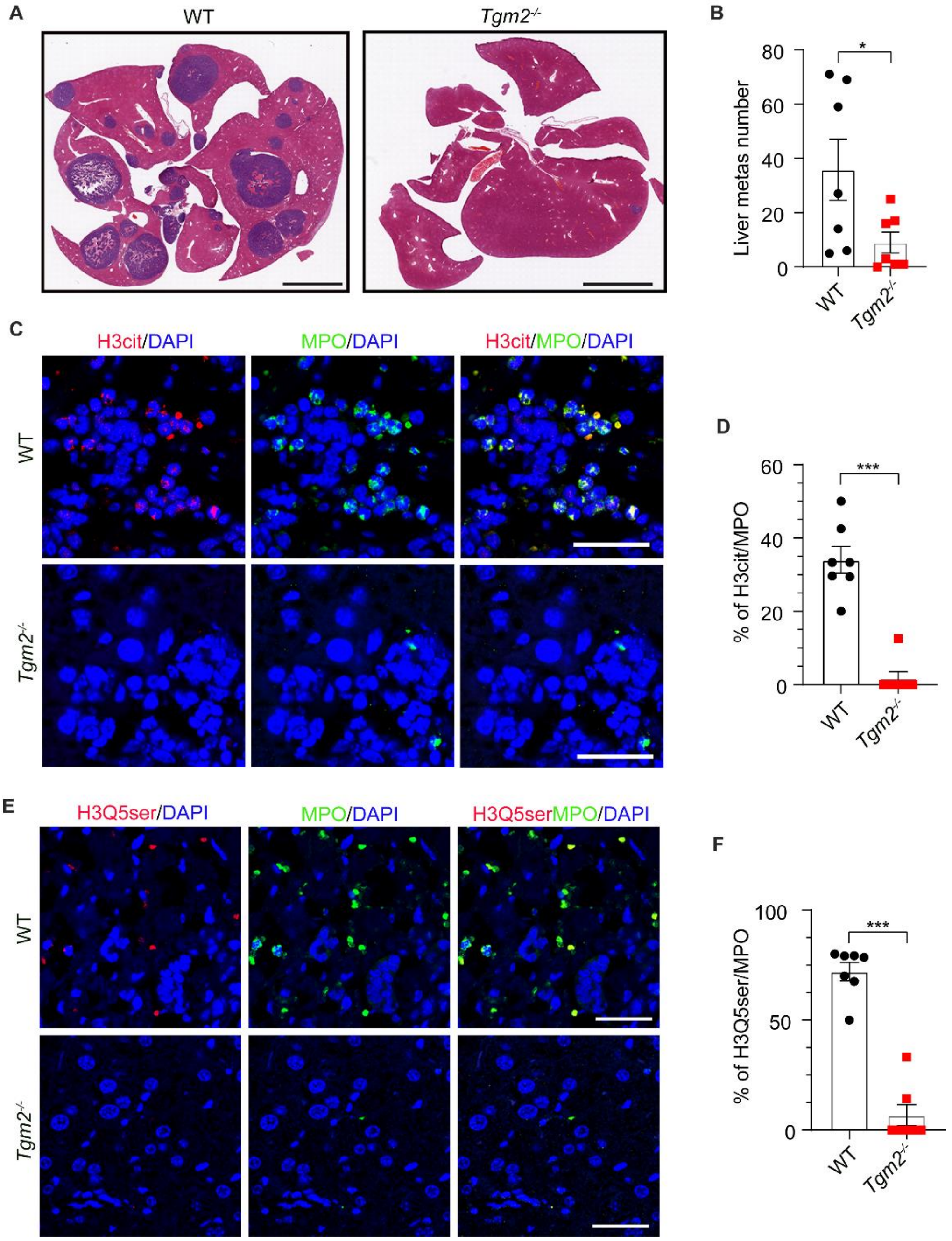

Supplemental Figure 6. The *Tgm*-KO recipient mice show decreased liver metastatic burden upon *Rb1*$^{\Delta/\Delta}$*Trp53*$^{\Delta/\Delta}$ NEPC organoid inoculation.

(A-B) H&E staining images **(A)** and quantification results **(B)** demonstrating reduced liver metastatic lesions in *Tgm2*$^{-/-}$ recipient tumor-bearing mice in contrast to WT tumor -bearing counterparts. The *Rb1*$^{\Delta/\Delta}$*Trp53*$^{\Delta/\Delta}$-organoids were intravenously inoculated into *Tgm2*$^{-/-}$ and WT recipients (n = 7 mice). In H&E staining images, scale bars = 5

mm. Data are shown as mean ± SEM, *P < 0.05 was assessed using the two-tailed student's *t*-test.

(C-D) IF staining images **(C)** and quantification data (**D**) showing reductions in neutrophil (MPO^+)-derived NETs formation in the liver of *Tgm2*$^{-/-}$ recipient tumor-bearing mice, as reflected by decreased MPO and H3cit signals (n = 7 mice). Scale bars in **(C)** = 50 μm.

(E-F) IF images **(E)** and related quantification results (**F**) showing decreases in H3Q5ser modifications in MPO^+ neutrophils in the liver of *Tgm2*$^{-/-}$ recipient tumor-bearing mice, as reflected by decreased MPO and H3Q5ser signals (n = 7 mice). Scale bars in **(E)** = 50 μm.

For statistics in **(D) and (F)**, data are presented as mean ± SEM, ***P < 0.001 were assessed using Mann-Whitney tests.

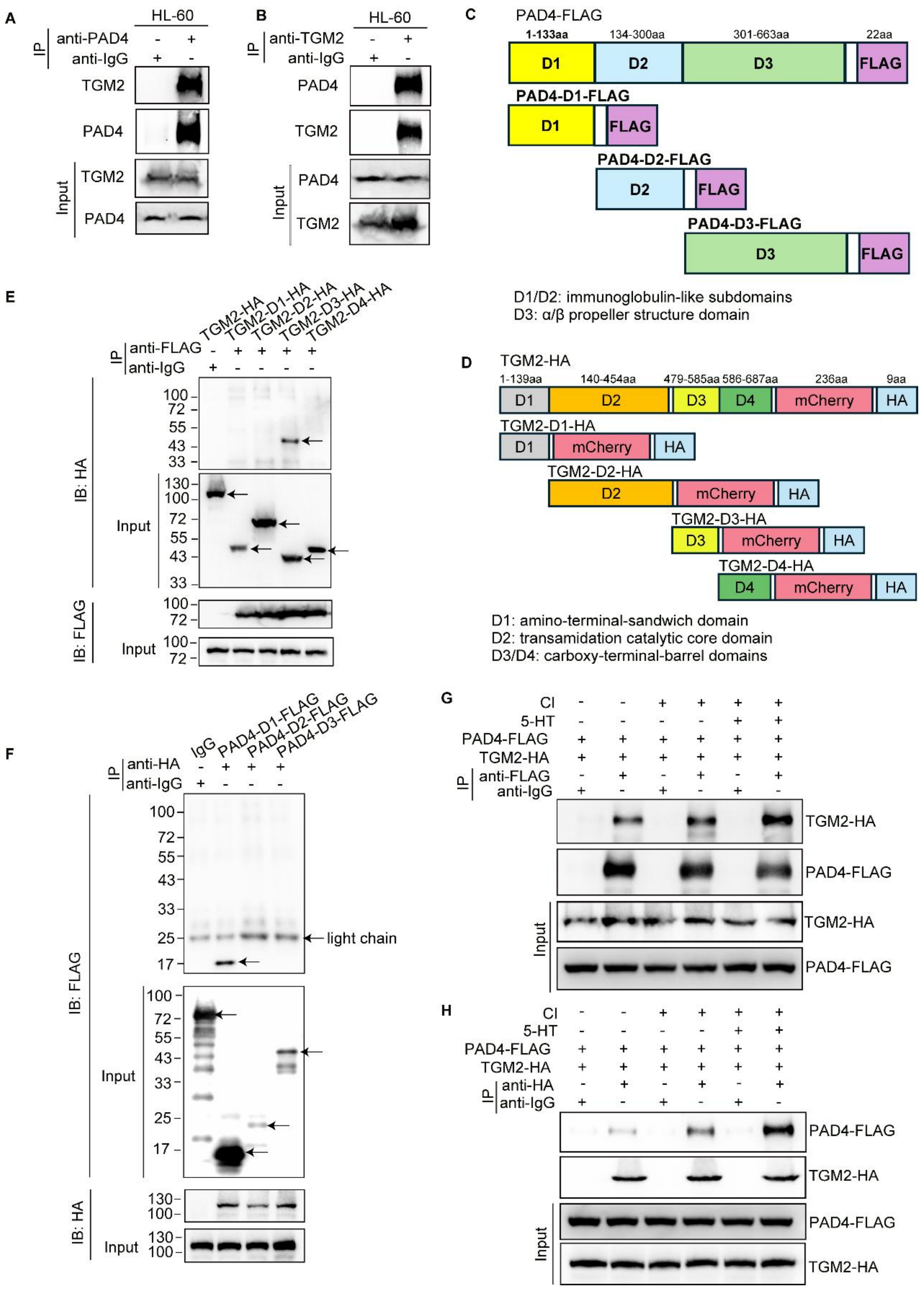

Supplemental Figure 7. TGM2 binds with PAD4 and 5-HT enhances their physical association.

(A-B) Immunoprecipitation (IP) assay shows the endogenous interaction between

PAD4 and TGM2 via IP endogenous PAD4 using anti-PAD4 antibody **(A)** or IP endogenous TGM2 using anti-TGM2 antibody **(B)** in HL-60 granulocytes.

(C-D) Diagram of truncated mutations of Flag-tagged PAD4 **(C)** and HA-tagged TGM2 **(D)** based on their well-characterized protein domains.

(E-F) Co-IP results showing that the binding of PAD4 and TGM2 is required for PAD4's first immunoglobulin-like domain (D1) and TGM2's third carboxy-terminal-barrel domain (D3).

(G-H) Exogenous addition of 5-HT (200 μM, treated for 4 h) further enhances the physical interaction between TGM2 and PAD4 in HEK-293T cells.

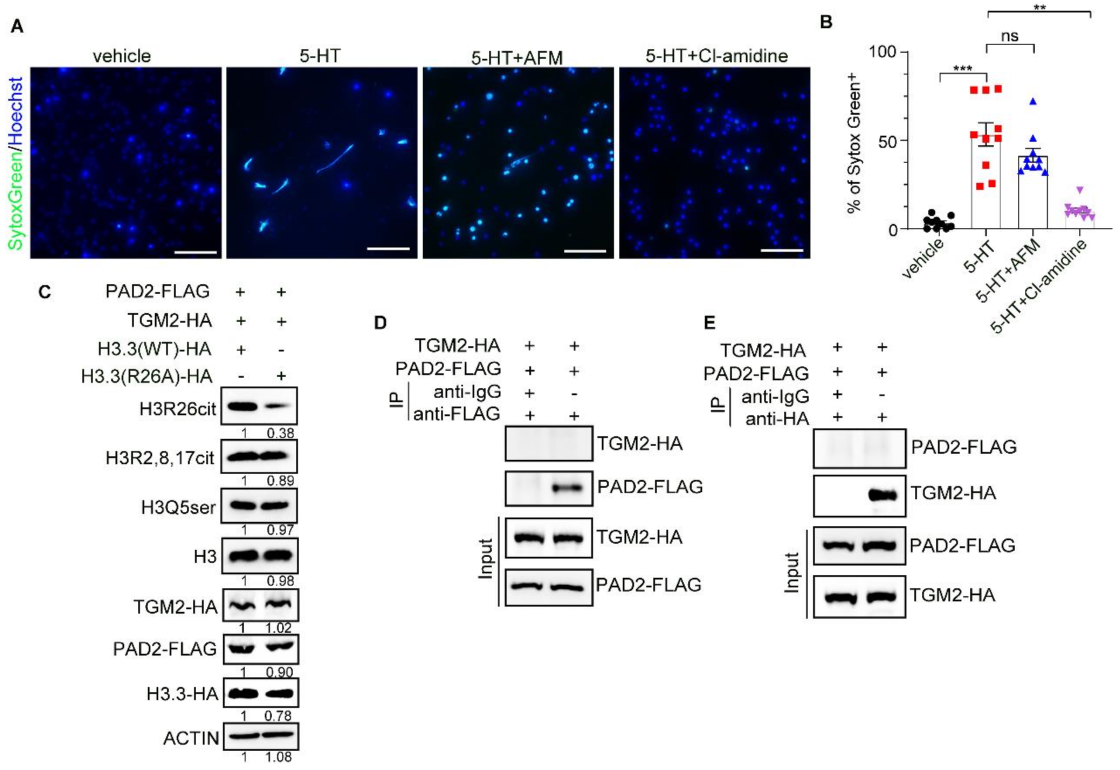

Supplemental Figure 8. PAD2 inhibition does not suppress NETs production or histone serotonylation.

(A-B) IF staining images **(A)** and quantification data **(B)** reveal that PAD2 inhibitor AFM-30a (AFM) does not affect 5-HT-induced NETs formation, as revealed by SytoxGreen signal **(A)** (n = 10 biological replicates). PAD4 inhibitor Cl-amidine was used as a positive control. Scale bars = 50 μm. Data in **(B)** were shown as mean ± SEM. **P < 0.01, ***P < 0.001 and ns = no significance were assessed using a Kruskal-Wallis test followed by Dunnett's test.

(C) Immunoblotting assay demonstrates that the H3.3(R26A) mutation leads to abrogated H3R26cit, but did not affect H3Q5ser and H3R2,8,17cit depositions.

(D-E) Co-IP assay shows that PAD2 did not associate with TGM2 either by IP Flag-tagged PAD2 **(D)** or HA-tagged TGM2 **(E)** in HEK-293T cells.

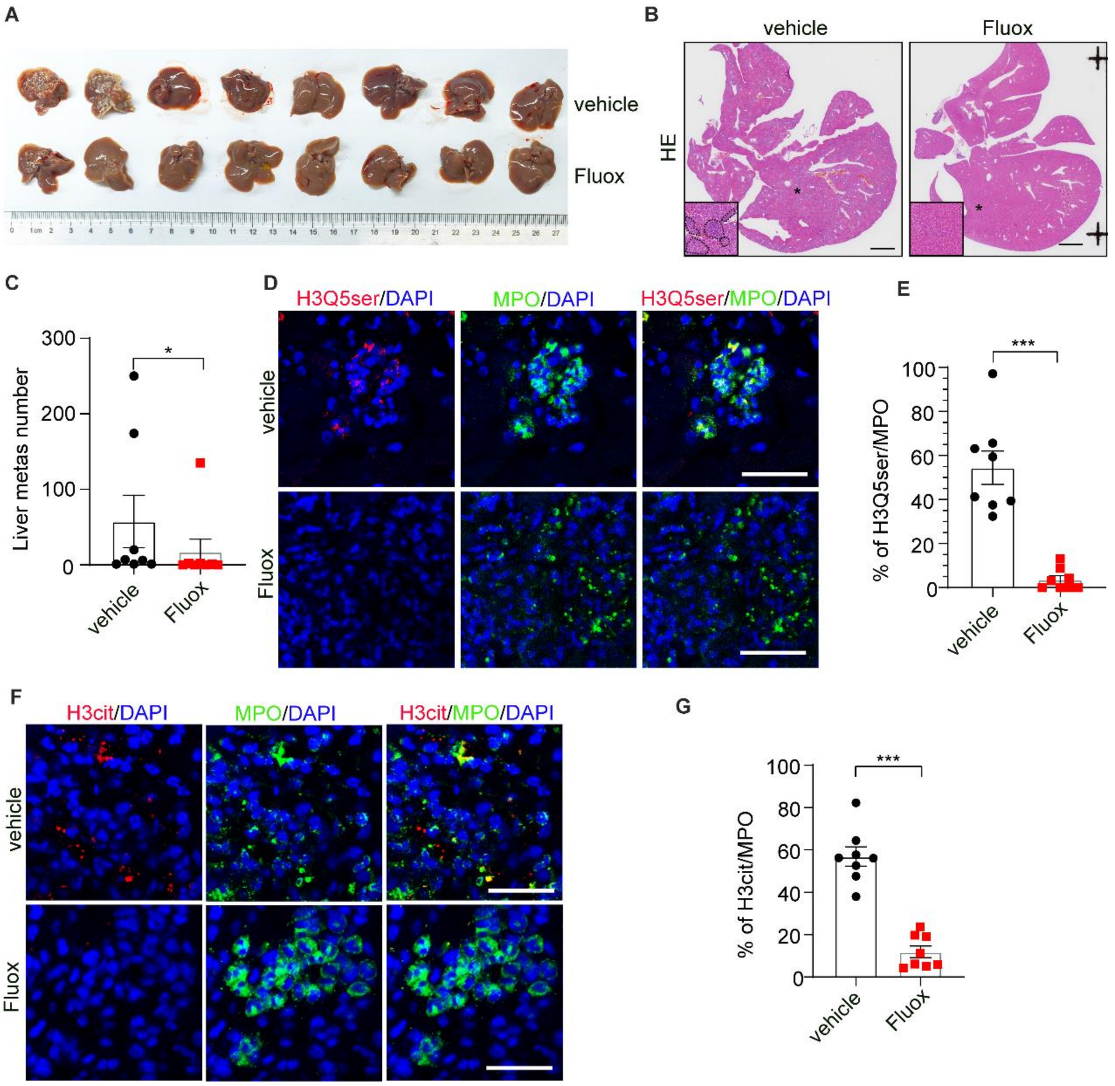

Supplemental Figure 9. SERT inhibitor fluoxetine represses NETs formation and liver metastasis in SCLC xenograft mice.

(A-C) In vivo experiments demonstrating a reduction of liver metastatic burden **(A)** in SERT inhibitor Fluoxetine (Fluox)-treated NCI-H82 SCLC-implanted mice as compared to vehicle via intravenous injection (n = 8 mice, each group). H&E staining **(B)** and quantification data **(C)** reveal significantly decreased liver metastatic foci numbers in Fluox-treated NCI-H82-inoculated mice. In H&E staining images, scale bars = 2 mm.

(D-E) IF images **(D)** and quantification results **(E)** on liver sections showing decreased

H3Q5ser deposition after fluoxetine treatment in NCI-H82 cells-inoculated nude mice (n = 8 mice). Scale bars = 50 μm.

(F-G) IF images **(F)** and quantification data **(G)** on liver sections showing a decreased NETs formation (H3cit^{+} signal) in neutrophils after fluoxetine treatment in NCI-H82 cells-inoculated nude mice (n = 8 mice).

Data in **(C)** were presented as mean ± SEM, and *P < 0.05 was assessed using a Mann-Whitney test. Data in **(E)** and **(G)** were shown as mean ± SEM, and ***P < 0.001 was assessed using the two-tailed student's *t*-test.

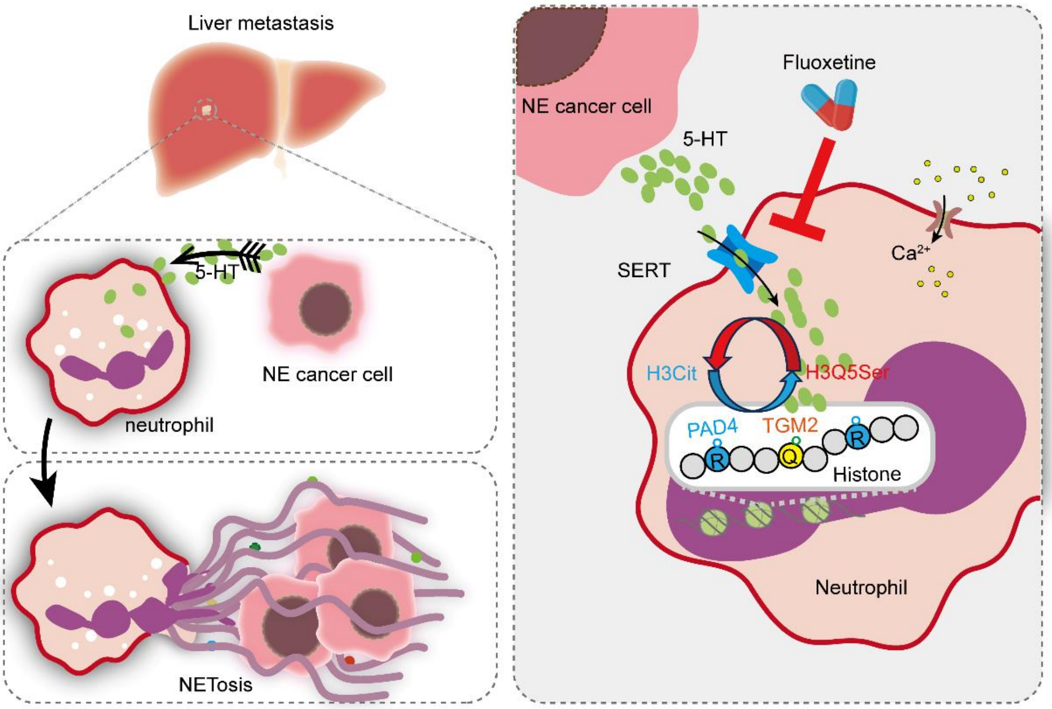

Graphical abstract of this study.

5-HT derived from NEPC induces TGM2-catalyzed histone serotonylation (H3Q5Ser) and promotes PAD4-mediated histone citrullination (H3cit) in neutrophils. TGM2 and PAD4, the epigenetic writers, associate with each other, inducing a mutually reinforcing effect between these two histone modifications. This results in enhanced NET formation, which facilitates the recruitment of disseminated cancer cells and promotes liver metastasis. Genetic ablation or pharmacological targeting of TGM2, or inhibition of the 5-HT transporter (SERT) by the FDA-approved antidepressant drug fluoxetine reduces H3Q5Ser and H3Cit modifications, suppresses NETs formation and effectively inhibits liver metastasis of NEPC and thyroid medullary cancer.

Supplemental Tables

Supplemental Table 1. The antibodies and reagents used in this study.

Name	Vendor	Catalog
**Antibodies**		
anti-H3R2,8,17cit antibody	Abcam	ab5103
anti-H3Q5ser antibody	Millipore	ABE1791
anti-PAD4 antibody	Abcam	ab50247
anti-TGM2 antibody	ThermoFisher	MA5-12739
anti-β-Actin antibody	Abclonal	AC026
anti-Histone 3 antibody	Cell Signaling Technology	4499
anti-HA-Tag antibody	Cell Signaling Technology	2367
anti-DDDDK-Tag antibody	Abclonal	AE005
anti-Myeloperoxidase (MPO) antibody	Abcam	ab300650
anti-CD66b antibody	Biolegend	392902
CD45.2-PerCP-Cy5.5 antibody	Biolegend	109828
CD11b-FITC antibody	Biolegend	101206
Ly-6G-PE-Cy7 antibody	Biolegend	127617
anti-H3R26cit antibody	Abcam	ab212082
anti-NCAM1 antibody	CST	99746S
**Chemicals, peptides, and recombinant proteins**		
LDN-27219	Targetmol	T11831
Fluoxetine	Targetmol	T0450L

Cl-amidine hydrochloride	Targetmol	T10831
AFM-30a hydrochloride	MCE	HY-125099A
5-HTP	MCE	HY-N0122
SB-224289 hydrochloride	MCE	HY-101105A
DNase I	Worthington	LS006322
MNase	Worthington	LS004797
Percoll	Yeasen	40501ES60
Histopaque®-1119	Sigma	11191
SytoxGreen	Beyotime	C1070M
Hoechst	Invitrogen	H3570
Mounting Medium with DAPI	Vectashield	H-1200
DMSO	Sigma	D2065
PEG400	Sangon	A611781
Tween-80	Sangon	A100442
TGM2 protein	SinoBiological	11095-H07B
PAD4 protein	SinoBiological	11072-H07B
H3 peptide	Genscript	Synthesized
H3cit-modified peptide	Genscript	Synthesized
H3Q5ser-modified peptide	Genscript	Synthesized
**Commercially available kits**		
Hieff NGS® G-Type In-Situ DNA Binding Profiling Library Prep Kit	Yeasen	12598ES12

Supplemental Table 2. The RT-qPCR primer sequences in this study.

**Primers**	**Species**	**Sequence (5' to 3')**
Tph1-F	Mouse	TGTTGACTGCGACATCAGCCGA
Tph1-R	Mouse	GGAAACCAAGGGACAGTCTCCA
Actb-F	Mouse	GTCCCAGACATCAGGGAGTAA
Actb-R	Mouse	TCGGATACTTCAGCGTCAGGA
ACTB-F	Human	CACCATTGGCAATGAGCGGTTC
ACTB-R	Human	AGGTCTTTGCGGATGTCCACGT
TGM2-F	Human	TGTGGCACCAAGTACCTGCTCA
TGM2-R	Human	GCACCTTGATGAGGTTGGACTC
PAD4-F	Human	GCACAACATGGACTTCTACGTGG
PAD4-R	Human	CACGCTGTCTTGGAACACCACA
TPH1-F	Human	TTCTGACCTGGACCATTGTGCC
TPH1-R	Human	ACGGTAGACATTGTCTTTGAAGCC

Supplemental Table 3. The shRNA sequences in this study.

shRNA	Sequence (5' to 3')
Scramble	CCTAAGGTTAAGTCGCCCTCG
sh*Tph1*-1	GCCATGAATGAGTTGCGGTAT
sh*Tph1*-2	GTGAACTCAAACATGCACTTT
sh*TPH1*-1	CTTGCAGAGAGTATCTCAA
sh*TPH1*-2	CCGAAAATCAAAAAGAAGA

sh*PAD4*-1	CCGCAGCTCTTCAAGCTCA
sh*PAD4*-2	AAGCTCAAAGAGTTCTCTA
sh*TGM2*-1	CGCGTCGTGACCAACTACA
sh*TGM2*-2	AAGTACCTGCTCAACCTCA

Supplemental Methods and Materials

Human NEPC liver metastasis tissues and blood samples.

Paraffin-embedded primary tumor blocks (n = 4 patients) or the core needle-punctured NEPC liver metastasis biopsies (n = 5, patients) were obtained from the Department of Urology, Ren Ji Hospital. For the human neutrophil extracellular traps (NETs) formation assay, the whole blood from healthy donors were obtained from the Physical Examination Center at Renji Hospital.

Primary neutrophil isolation.

Neutrophils were isolated from mice bone marrow following the protocol described by Xiao et al.(2). Neutrophils from human peripheral blood of healthy donors were performed according to a published paper by Brinkmann et al. (3).

Two-chamber tumor migration assay.

The two-chamber migration assay was conducted based on a previously described protocol (4). Briefly, NETs induced by PMA for 12-16 hours (h) at 37°C were added to the lower chamber, and *Rb1*$^{\Delta/\Delta}$*Trp53*$^{\Delta/\Delta}$ NEPC-like prostate cancer cells were seeded onto the upper chamber of 8 μm PoreSize. 4 h later, the migrated cancer cells from upper chamber to lower chamber were fixed with 4% PFA for 10 min at room temperature (RT) and stained with crystal violet for 15 min. The migrated cancer cells were then counted and quantified using ImageJ software.

Adherence assay.

The adherence assay was performed according to a published paper by Yang *et al.* (5). In detail, the adherence of $Rb1^{\Delta/\Delta}Trp53^{\Delta/\Delta}$ NEPC-like prostate cancer cells to NETs was evaluated using 24-well plates which were precoated with NETs induced by PMA for 12-16 h at 37°C. NEPC cancer cells from $Rb1^{\Delta/\Delta}Trp53^{\Delta/\Delta}$ mice were suspended in serum-free medium and were allowed to adhere to the bottom of the plate for 60 min at 37°C. Non-adherent cells were removed by gently washing with PBS for 3 times, adherent cells were fixed with 4% PFA for 10 min at RT and stained with crystal violet for 15 min. Cell adherence was counted and quantified using Image-J software.

HL-60 cell differentiation and calcium ionophore induction assay.

To induce human HL-60 cells differentiate into granulocytes, HL-60 cells were cultured in IMDM medium with 20% FBS and stimulated with N,N-Dimethylformamide (DMF) (0.51% in the medium) for 4 days at a starting density of 2.5×10^6 cells per milliliter (mL). 4 days later, the HL-60 cells were resuspended in Locke's solution (0.1% FBS, 10mM Hepes-HCl, 150 mM NaCl, 2 mM CaCl2, 5 mM KCl and 0.1% glucose, pH = 7.3). HL-60 cell density was adjusted to 3.5×10^7 cells/mL and stimulated with calcium ionophore (Cl) (4 μM) at 37°C for 4 h. For LDN-27219 treatment, HL-60 cells were treated with TGM2 inhibitor LDN-27219 (10 μM) for 3 days after HL-60 cells were induced and differentiated into granulocytes. The media were then replaced with Locke's solution with 0.1% FBS and further incubated for 12 h. Next, cells were incubated with 4 μM Cl and 300 μM 5-HT for 4 h at 37°C. For fluoxetine treatment,

HL-60 cell-derived granulocytes were treated with the SERT inhibitor fluoxetine (10 μM) in Locke's solution for 12 h. Next, cells were incubated with Cl (4 μM) and 5-HT (300 μM) for 4 h at 37°C before collection. For Cl treatment, HL-60 cell-derived granulocytes were treated with the PAD4 inhibitor Cl-amidine (100 μM) for 4 h using the Locke's solution.

MNase Digestion Assay.

The MNase digestion assay was performed according to a previous study by Wang et al.(6). Briefly, cells were permeabilized with PBST (PBS with 0.1% Triton X-100), and then collected, and centrifuged at 2,000 rpm at 4°C for 10 min. The cell pellet was resuspended in 250 μL of N2 buffer (pH 6.5, 10 mM Pipes, 0.5 mM sodium metabisulfate, 5 mM $MgCl_2$, and 0.5 mM benzamidine-HCl freshly supplemented with 0.5 mM DTT, 0.1 mM PMSF, and 5 mM $CaCl_2$). Samples were placed on ice and were treated with 0.5 unit RNase A. 0.2 μL MNase (Worthington, LS004797) was added to a 50 μL volume reaction system. Reactions were conducted at 37°C and terminated at indicated time points by the addition of EDTA at the concentration of 10 mM. Next, 75 μL H_2O, 30 μL 10% SDS, 25 μL 4 M NaCl and 200 μL phenol-chloroform were added sequentially to the digested samples and mixed by vortex. The samples were then centrifuged at 13,000 rpm for 10 min. Sample stratification was observed and the aqueous phase was extracted. 1/3 volume of 7.5 M NH_4Ac (pH 7.6) and 1 volume of isopropanol were sequentially added to the aqueous phase. These samples were kept at RT for 10 min to precipitate the DNA, and centrifuged at 13,000 rpm for 10 min. The

DNA was then washed with precooled 70% ethanol for 3 times, air dried, and resuspended with 10 μL H_2O with DNA loading buffer, and followed by DNA electrophoresis.

Immunofluorescence (IF) staining.

For IF staining, mouse prostate tumors were fixed in 4% PFA overnight and then dehydrated in 30% sucrose for 24 h. Tissue was embedded in OCT compound and frozen at −80 °C refrigerator for 30 min. Frozen sections were cut into 6 μm-thick sections. Sections were then subjected to heat-induced antigen retrieval with 0.01 M citrate antigen retrieval solution (PH 6.0). Sections were then blocked with 10% donkey serum for 1 h at RT. Primary antibodies were diluted with 1% donkey serum and 0.3% Triton X-100 in PBS and were incubated overnight at 4°C. The slides were then washed 3 times with PBS and incubated with secondary antibodies at RT for 1 h. Slides were then washed 3 times with PBS and were mounted using Vector Shield antifade Mounting Medium with DAPI. Image acquisition was performed on a Zeiss 710 confocal microscope.

Flow cytometry.

Liver with metastatic lesions were harvested and cut into small pieces, minced, and digested in RPMI-1640 culture medium (A4192301, Gibco) containing 2% FBS (10270-106, Gibco), 0.2 mg/mL collagenase I (17100017, Gibco), 0.2 mg/mL collagenase IV (17104019, Gibco), 0.01 mg/ml Dnase I (07900, STEMCELL

Technologies), and 0.02 mg/mL dispase (A002100-0050, Sangon). Tissue digestion was performed on a shaker at 37°C for 0.5 h. The red blood cells were lysed using ACK lysis buffer (155 mM ammonium chloride, 10 mM potassium bicarbonate and 0.1 mM EDTA). The filtered single cells were blocked with the anti-CD16/32 antibody for 30 min on ice and then incubated with the indicated antibodies for 30 min. Flow cytometry analysis was performed on a BD Fortessa instrument, and data analysis was performed using FlowJo software.

Immunoblotting assay.

Cells were lysed in RIPA lysis buffer (ThermoFisher, 89901) supplemented with the protease inhibitor cocktail (MCE, HY-K0011) for 0.5 h. Next, samples were heated to 100°C for 10 min in protein loading buffer. Protein samples were separated by SDS-PAGE and electrophoresis and then transferred to polyvinylidene difluoride (PVDF) membranes. The membranes were blocked with 10% skimmed milk and incubated with the indicated primary antibodies overnight at 4°C, followed by incubation with horseradish peroxidase (HRP)-conjugated secondary antibody. Protein bands were detected using the ECL chemiluminescence kit (Millipore, P90720).

Co-immunoprecipitation (Co-IP) assay.

For endogenous proteins, HL-60 cells-derived granulocyte pellets were washed with precold PBS and lysed with NETN buffer (150 mM NaCl, 5 mM EDTA, 50 mM Tris-HCl pH7.5, and 1% NP-40). For co-IP using exogenous transfected plasmids, HEK-

293T cells were transfected with the Flag-tagged PAD4 and HA-tagged TGM2 plasmids or truncated plasmids. Transfected cells were collected 72 h after transfection and lysed with NETN buffer. Anti-IgG, anti-HA, or anti-Flag antibodies were added to each sample based on experimental design. Then, the mixtures were rotated at 4°C overnight for antibody incubations. Next, the protein A/G magnetic beads (B23202, Selleck) were then incubated with the mixture for 2 h at 4°C. Finally, the beads were washed with HNTG buffer (50 mM HEPES, 150 mM NaCl, pH 7.4,10% glycerol, and 1% Triton X-100) for three times. The beads were mixed with protein loading buffer (P0015, Beyotime) and boiled for 10 min at 98°C followed by immunoblotting assays.

Pull down assay.

Human recombinant TGM2 protein (11095-H07B, SinoBiological, 4 μg/mL) was incubated with recombinant human PAD4 protein (11072-H07B, SinoBiological, 4 μg/mL) at the in Locke's solution. Indicated antibodies were added to the mixtures and rotated for incubation at 4°C for 6 h. The peptides and their binding proteins were immobilized with protein A/G magnetic beads at 4°C for 4 h. Next, the magnetic beads were washed with HNTG buffer for three times, and were then mixed with protein loading buffer (P0015, Beyotime) and boiled at 98°C for 10 min followed by immunoblotting assays.

Enzymatic assay.

To evaluate TGM2-mediated transamidation of 5-HT to histone peptides, 10 μg of

recombinant $H3_{1-20}$ or $H3cit_{1-20}$ peptides were incubated with 0.25 μg recombinant TGM2 (SinoBiological, 11095-H07B) and 5 mM 5-HT in a final volume of 25 μL enzyme buffer (25 mM Tris-Cl, pH = 8, 5 mM $CaCl_2$, and protease inhibitors). The mixtures were incubated at RT for 3 h in the dark room. For evaluation of PAD4-mediated citrullination reactions, 10 μg of recombinant $H3_{1-20}$ or $H3Q5ser_{1-20}$ peptides were incubated with 0.25 μg of recombinant PAD4 (SinoBiological, 11072-H07B) in a final volume of 25 μL enzyme buffer (50 mM HEPES, pH = 7.5, 2 mM DTT, 10 mM $CaCl_2$, and protease inhibitors). Reactions were incubated at RT for 3 h in the dark room. Next, samples were heated to 98°C for 8 min in protein loading buffer. Peptides were then spotted onto nitrocellulose (NC) membranes. Membranes were allowed to dry for 1 h and then blocked with 10% skimmed milk (dissolved in PBST solution), followed by incubation with appropriate primary and secondary antibodies. Ponceau S staining was used as a loading control for peptide quantity. Peptides were synthesized by GenScript Biotech Ltd.

CUT&Tag assay.

HL-60 cells were stimulated with 0.51% DMF for 4 days to differentiate into granulocytes. Then, the granulocytes were treated with Cl and 5-HT for 4 h before collection. The CUT&Tag assay was performed as previously described (7). According to the manufacturer's protocol (Yeasen Biotech, 12598ES12), the samples were incubated with the primary antibody of H3Q5ser (1:50; MERCK, ABE1791) and H3cit (1:100; Novus Biologicals, NB100-57135) respectively at 4°C overnight, with the

secondary IgG (H+L) antibody (Abcam, ab6702) at RT for 1 h, and with pA/G-Tn5 transposase at RT for 1 h. The labeling process takes 1 h. After incubation with Proteinase K at 55°C for 30 min, the DNA was extracted and was then subjected to DNA library amplification. The CUT & Tag data analysis was performed on an Illumina Hi-seq-PE150 sequencing instrument (Novogene). Upstream analysis was performed using nf-core/cutandrun 2.0.0. Heatmaps and summary plots were generated by deepTools (v.3.3.1). For correlation analysis, the complete genome was fragmented into small bins of 20-kb resolution using multiBigwigSummary function. The Spearman's correlation coefficients of each sample was calculated using plotCorrelation function.

Reference

1. Beltran H, Prandi D, Mosquera JM, Benelli M, Puca L, Cyrta J, et al. Divergent clonal evolution of castration-resistant neuroendocrine prostate cancer. *Nature medicine.* 2016;22(3):298-305.
2. Xiao Y, Cong M, Li J, He D, Wu Q, Tian P, et al. Cathepsin C promotes breast cancer lung metastasis by modulating neutrophil infiltration and neutrophil extracellular trap formation. *Cancer cell.* 2021;39(3):423-37.e7.
3. Brinkmann V, Laube B, Abu Abed U, Goosmann C, and Zychlinsky A. Neutrophil extracellular traps: how to generate and visualize them. *Journal of visualized experiments : JoVE.* 2010(36).
4. Zhuang X, Zhang H, Li X, Li X, Cong M, Peng F, et al. Differential effects on lung and bone metastasis of breast cancer by Wnt signalling inhibitor DKK1. *Nat Cell Biol.* 2017;19(10):1274-85.
5. Yang L, Liu Q, Zhang X, Liu X, Zhou B, Chen J, et al. DNA of neutrophil extracellular traps promotes cancer metastasis via CCDC25. *Nature.* 2020;583(7814):133-8.
6. Wang Y, Li M, Stadler S, Correll S, Li P, Wang D, et al. Histone hypercitrullination mediates chromatin decondensation and neutrophil extracellular trap formation. *Journal of Cell Biology.* 2009;184(2):205-13.
7. Kaya-Okur HS, Wu SJ, Codomo CA, Pledger ES, Bryson TD, Henikoff JG, et al. CUT&Tag for efficient epigenomic profiling of small samples and single cells. *Nat Commun.* 2019;10(1):1930.
