# Supplementary material for: 5-HT orchestrates histone serotonylation and citrullination to drive neutrophil extracellular traps and liver metastasis
Source: J Clin Invest. 2025 Feb 4;135(8):e183544. doi: 10.1172/JCI183544 (PMC11996869; doi:10.1172/JCI183544)

Full uncropped gel related to **Figure 2G**

Nucleic acid gel from MNase assay in Figure 2G

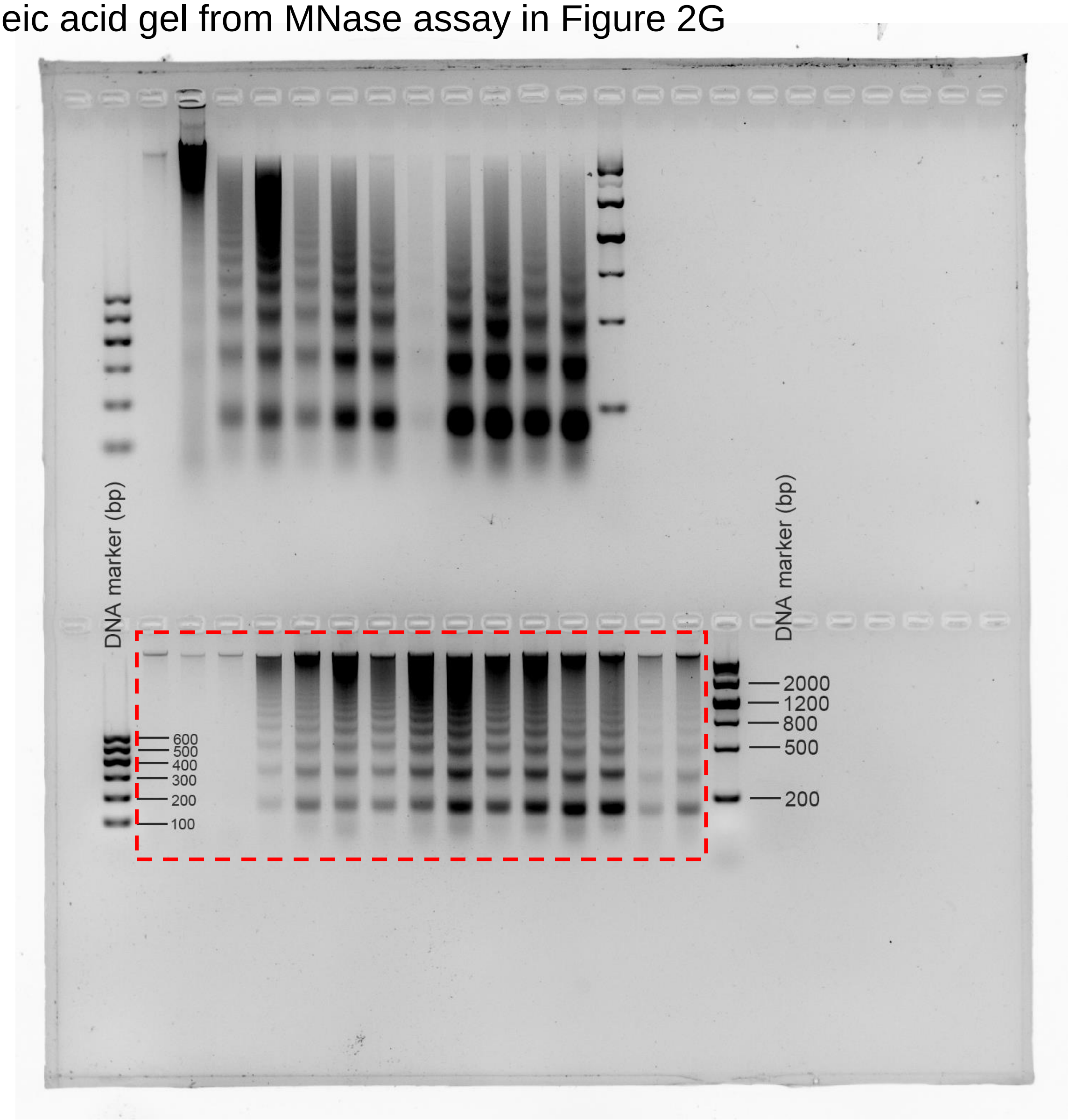

Full uncropped gel related to **Figure 3H**

Nucleic acid gel from MNase assay in Figure 3H

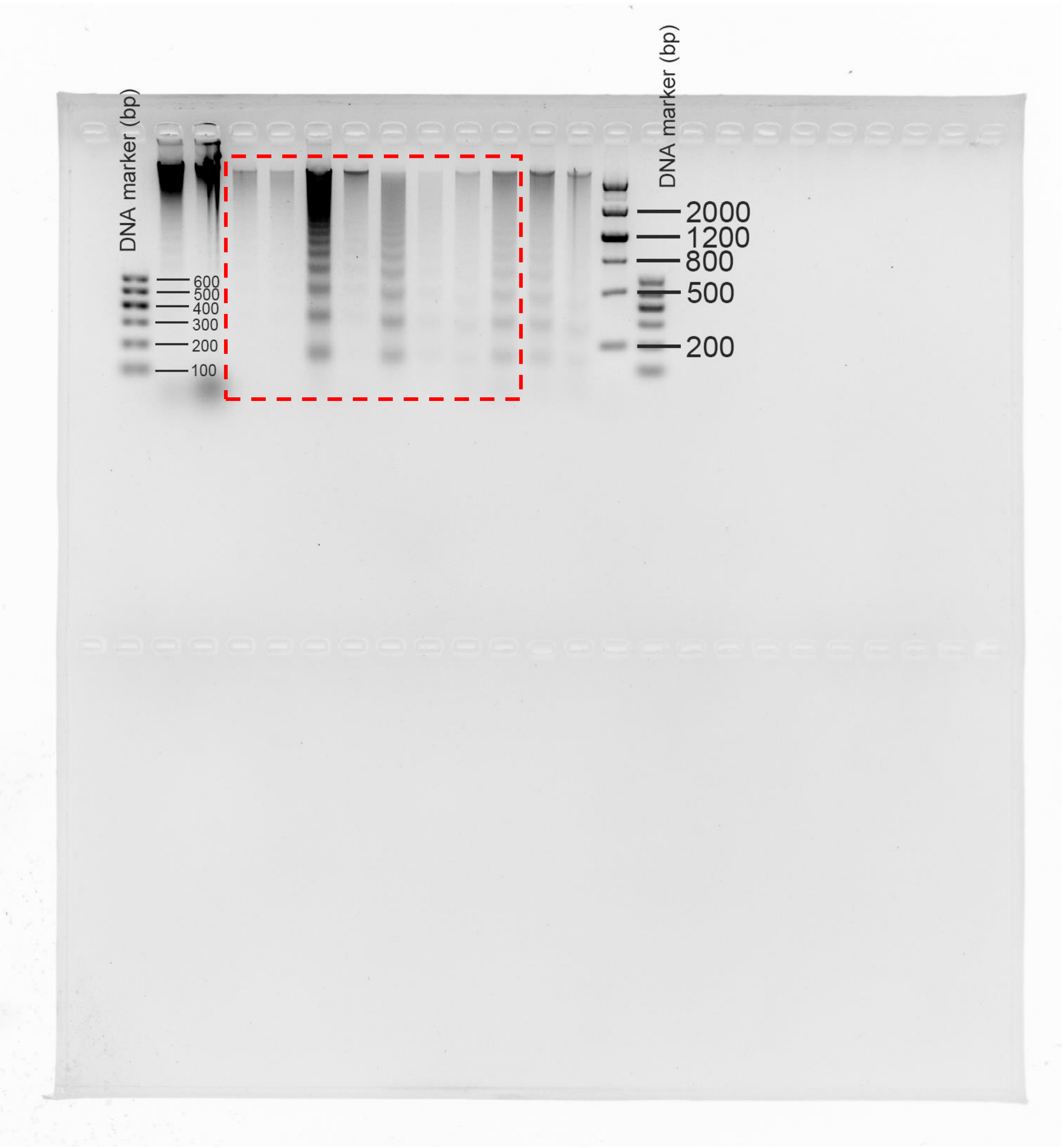

Full uncropped blots related to **Figure 4B**

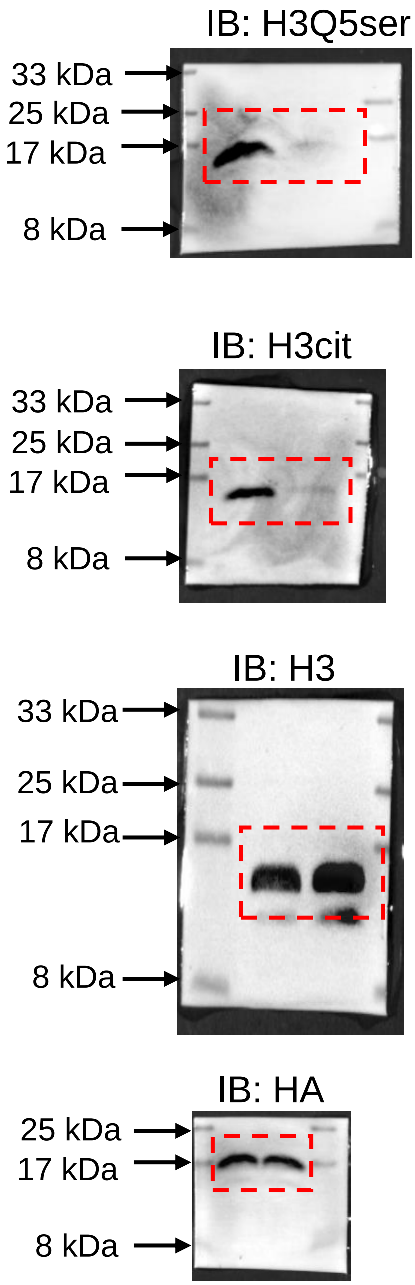

Full uncropped gel related to **Figure 4G**

Nucleic acid gel from MNase assay in Figure 4G

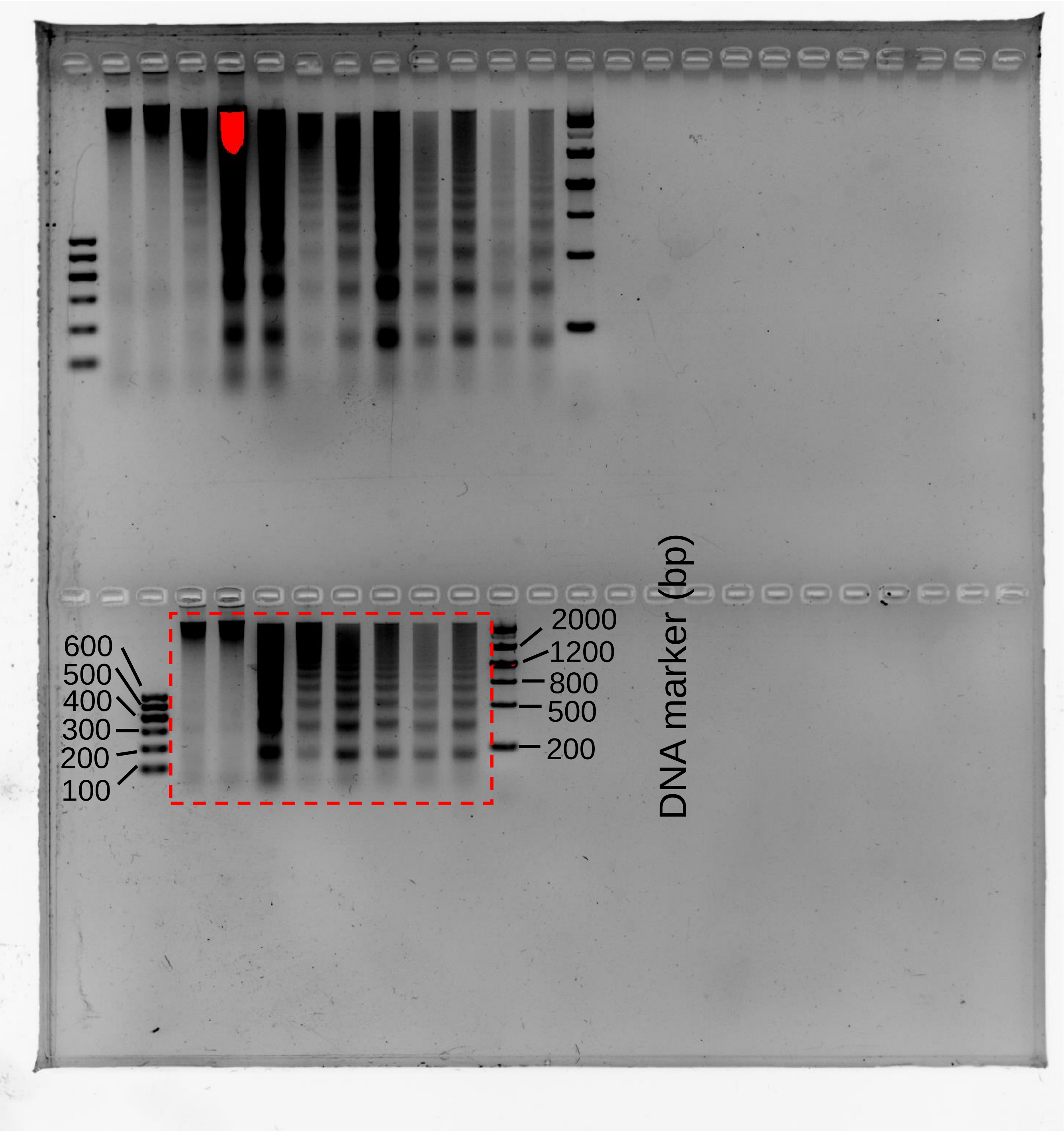

Full uncropped blots related to **Figure 6B**

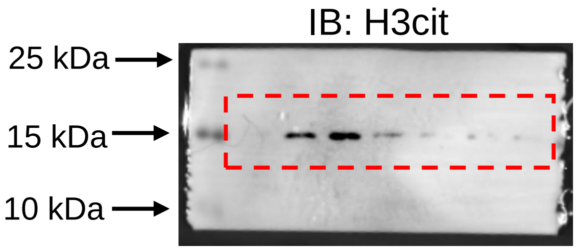

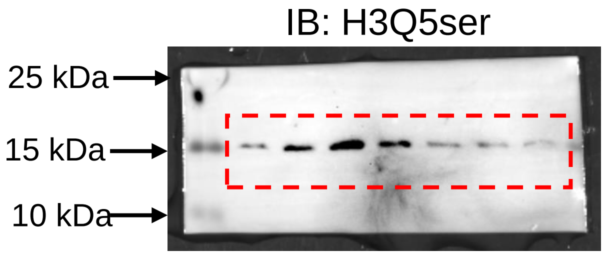

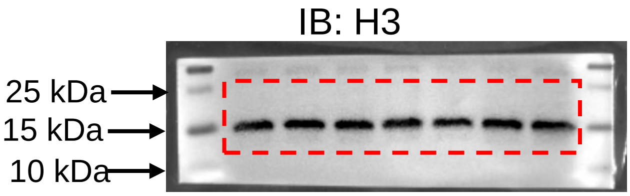

Full uncropped blots related to **Figure 6C**

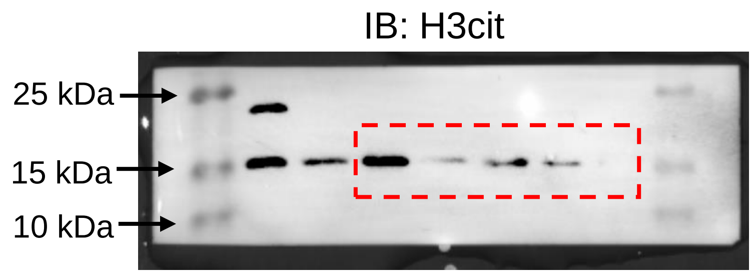

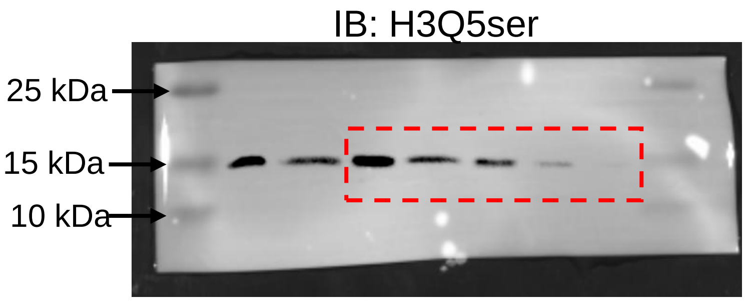

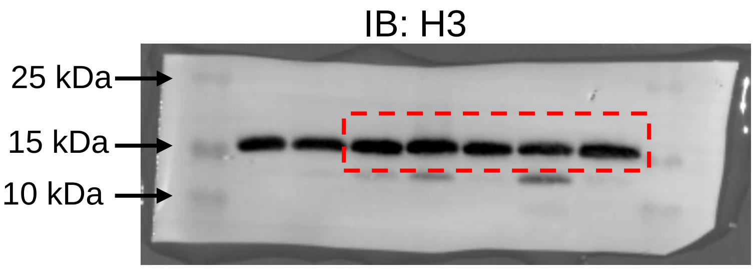

Full uncropped gel related to **Figure 6D**

Nucleic acid gel from MNase assay in Figure 6D

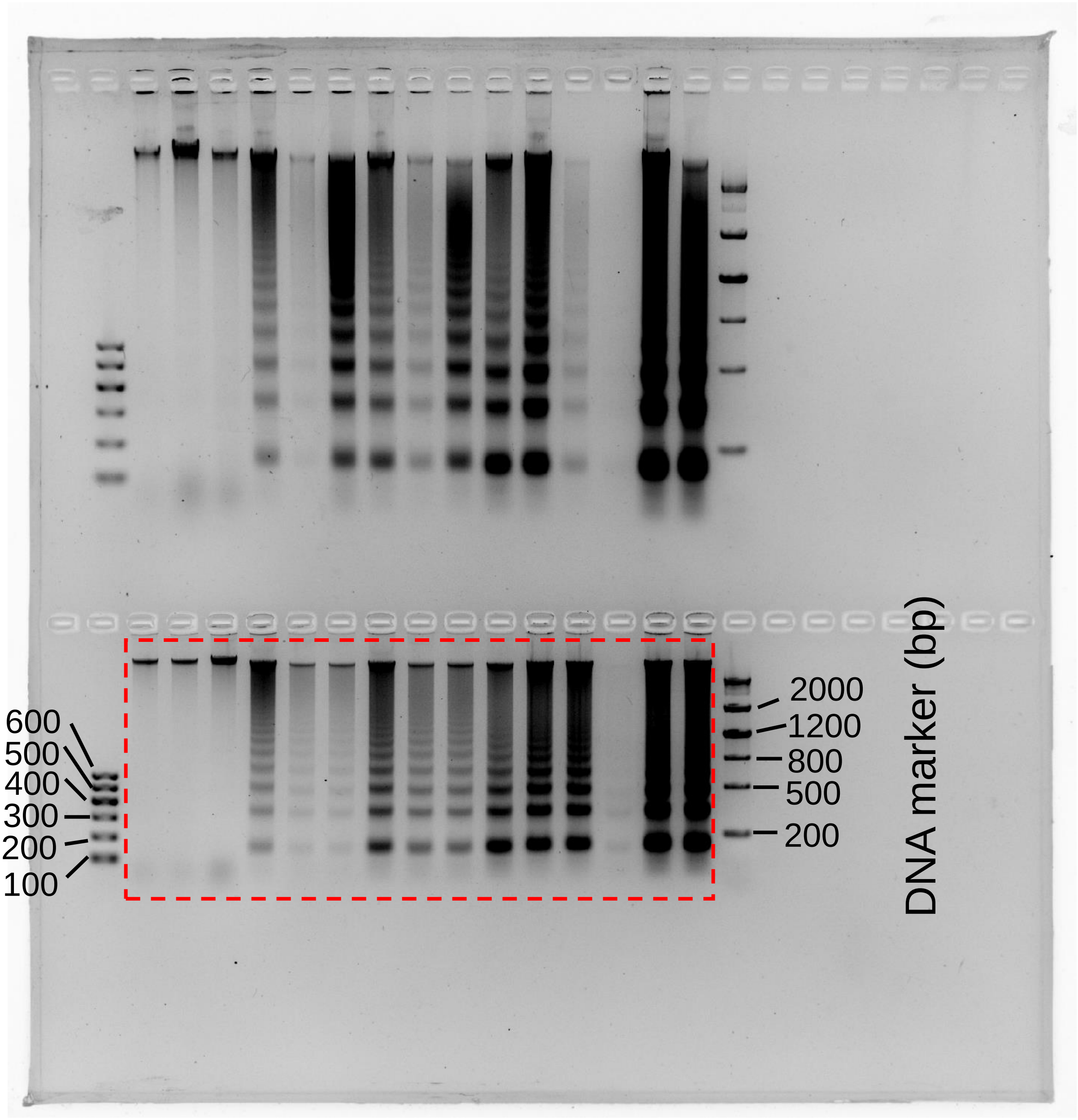

Full uncropped gel related to **Figure 6E**

Nucleic acid gel from MNase assay in Figure 6E

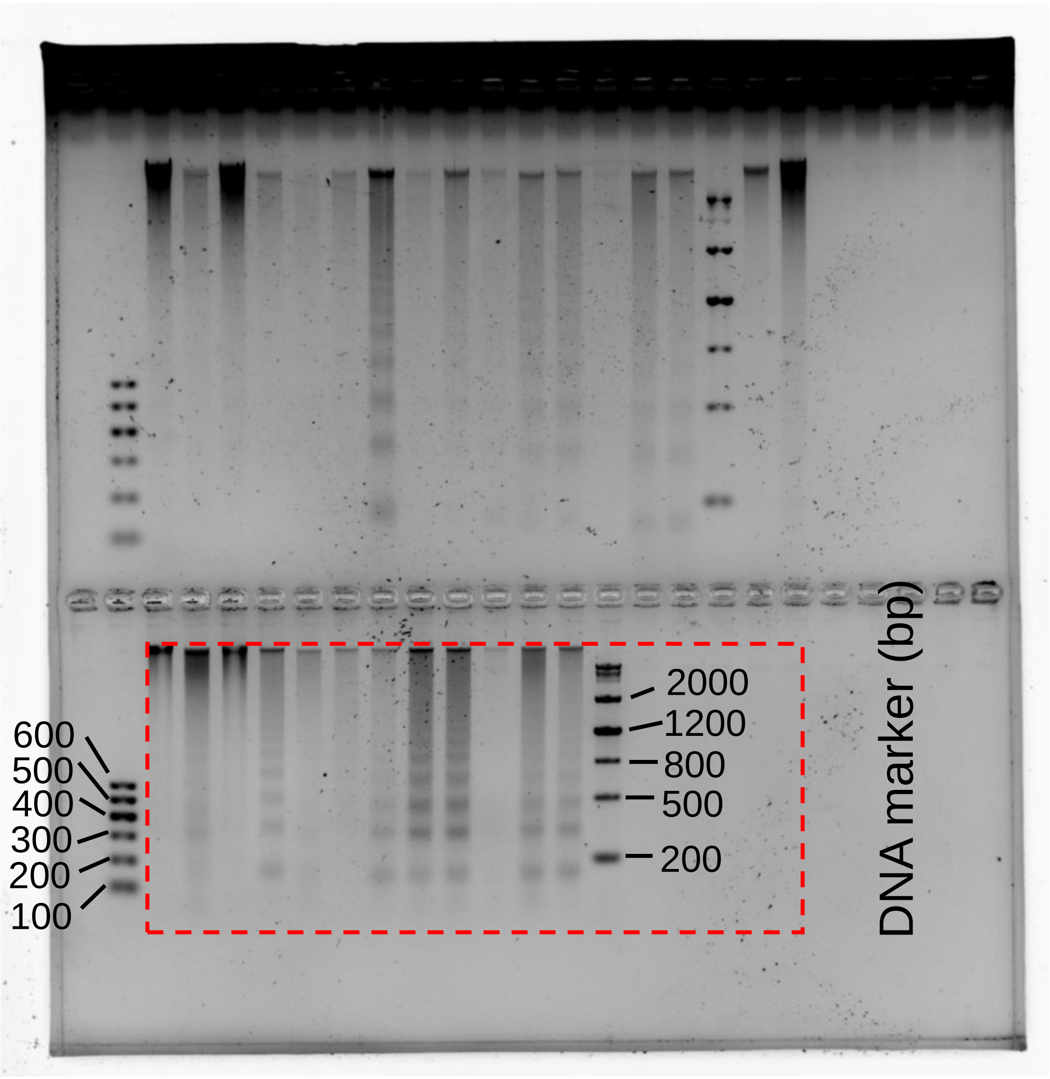

Full uncropped blots related to **Figure 6F**

IB: H3cit

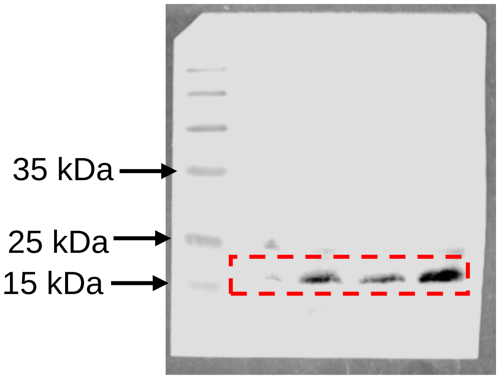

IB: PAD4-FLAG

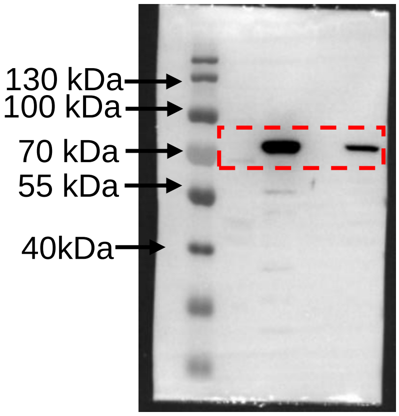

IB: H3Q5ser

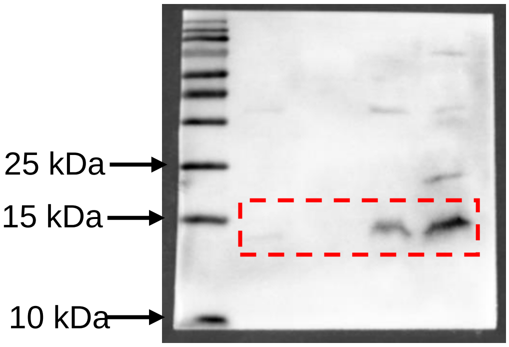

IB: TGM2-HA

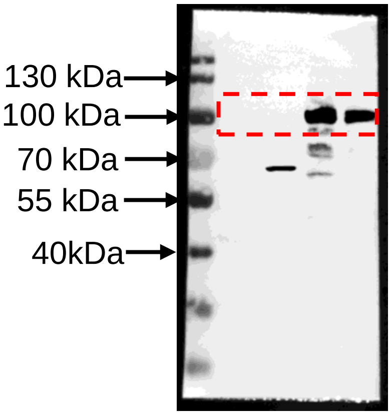

IB: H3

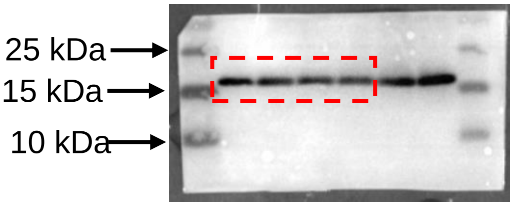

IB: ACTIN

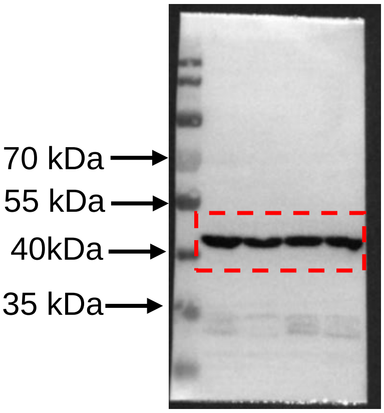

Full uncropped blots related to **Figure 6G**

IB: H3cit

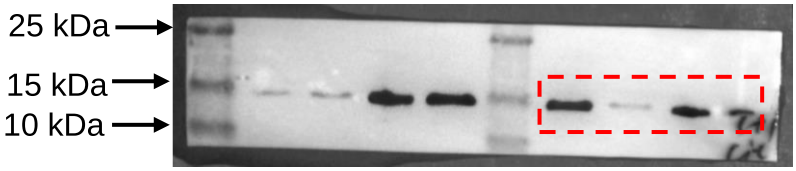

IB: H3Q5ser

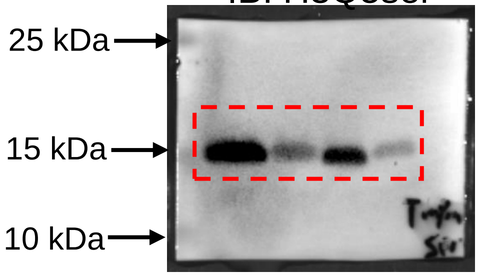

IB: H3

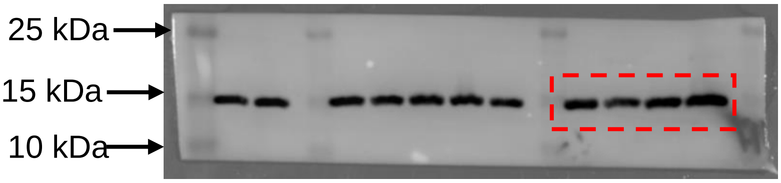

IB: PAD4-FLAG

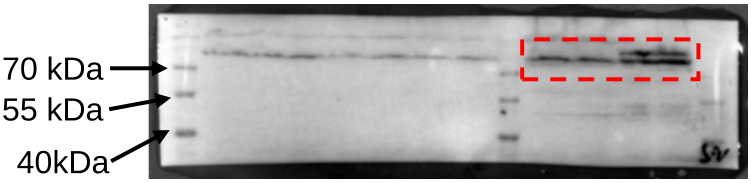

IB: TGM2-HA

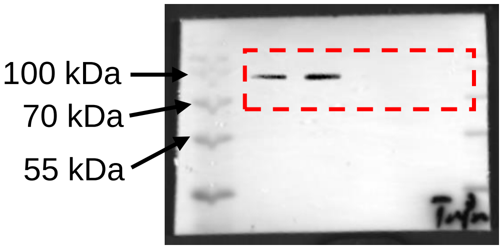

Full uncropped blots related to **Figure 6H**

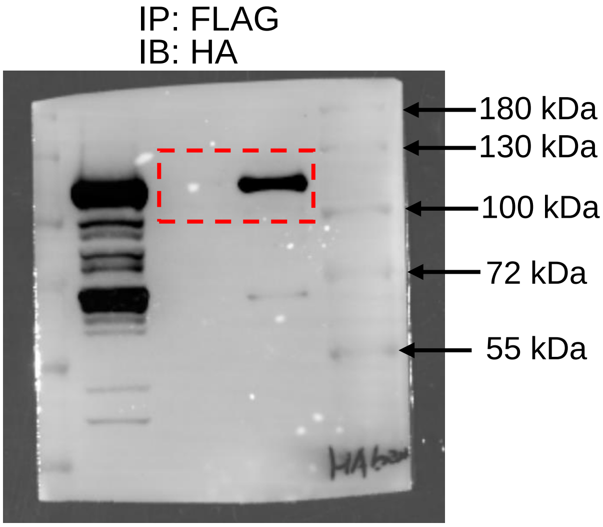

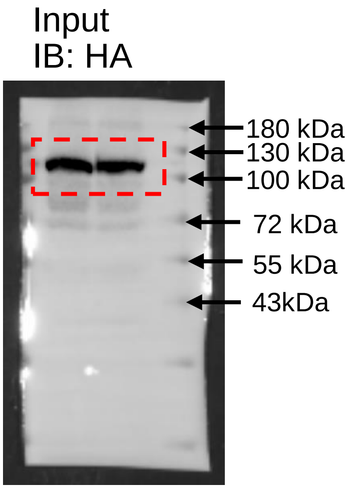

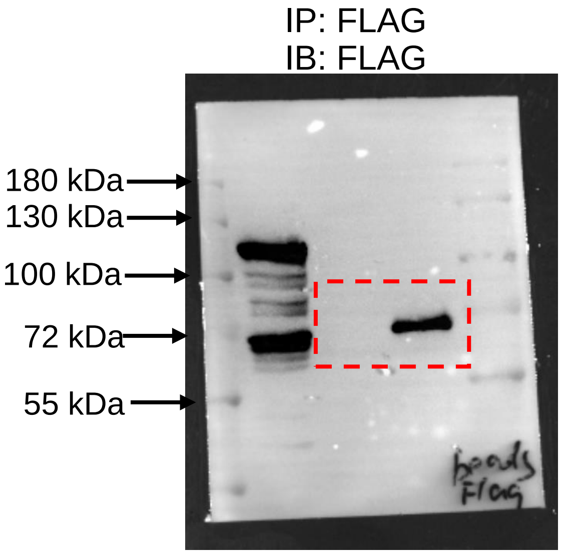

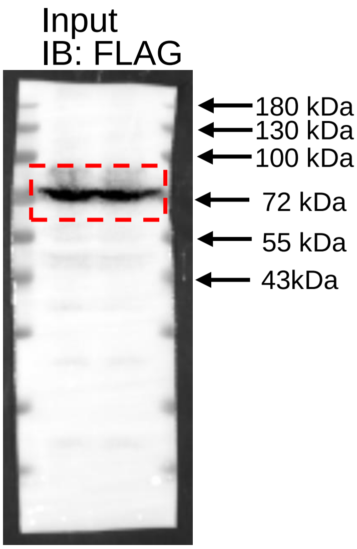

Full uncropped blots related to **Figure 6I**

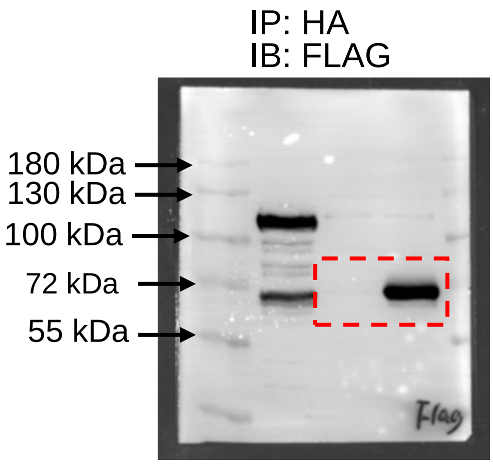

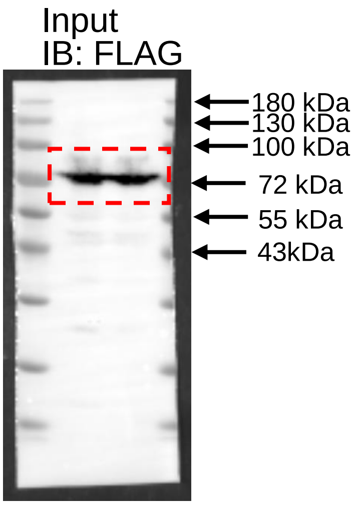

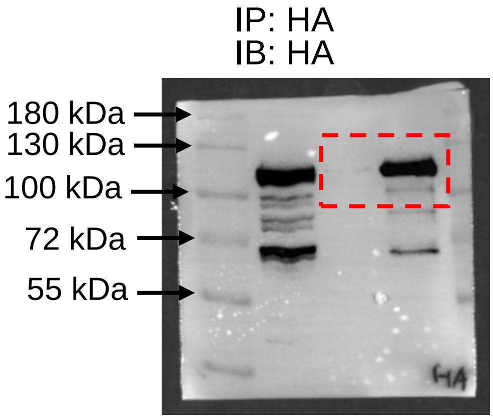

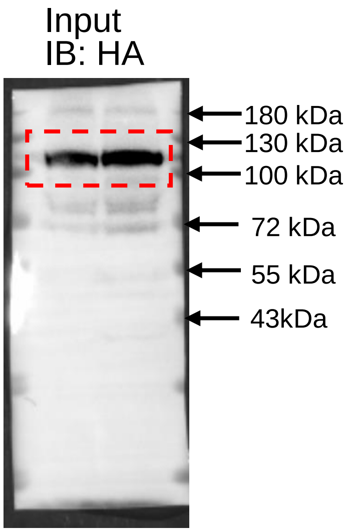

Full uncropped blots related to **Figure 6J**

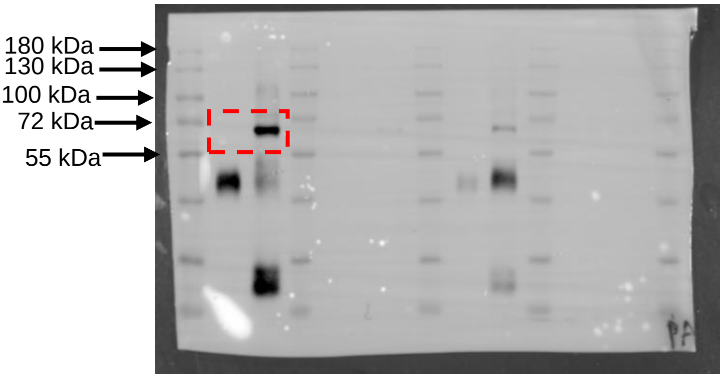

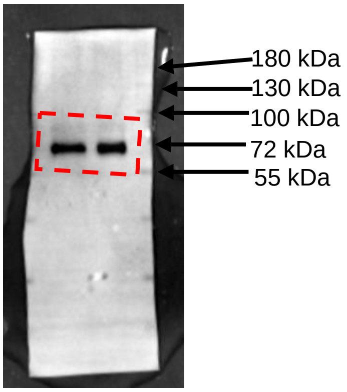

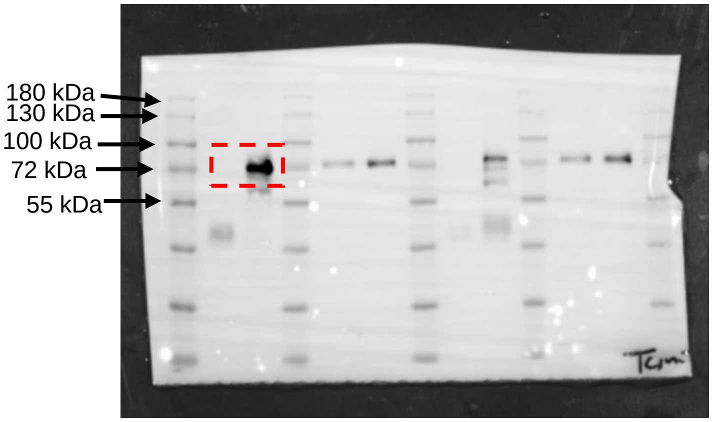

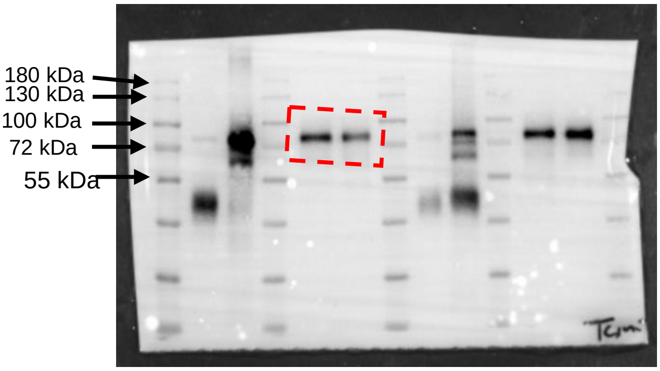

Full uncropped blots related to **Figure 6K**

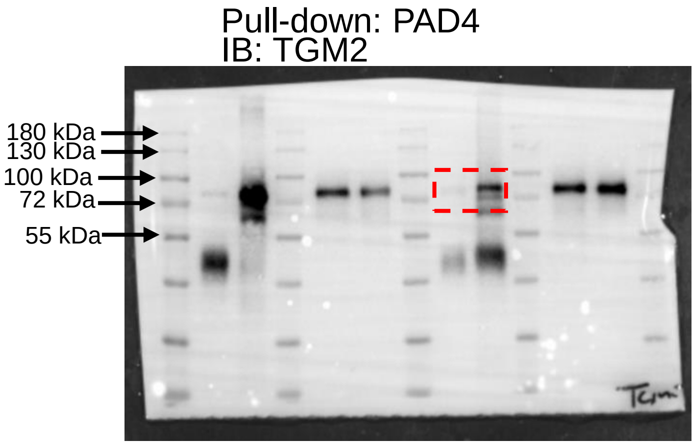

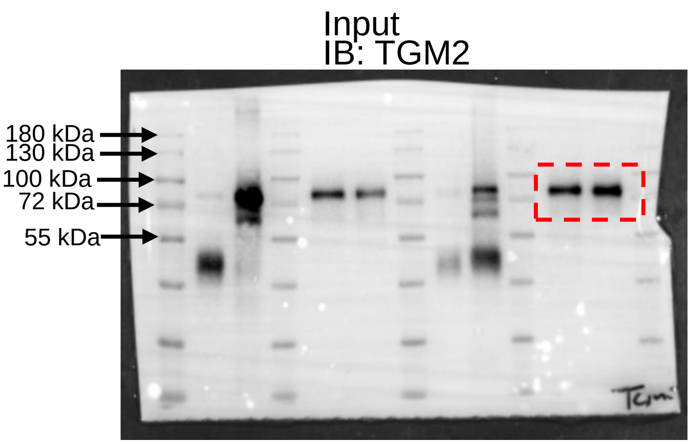

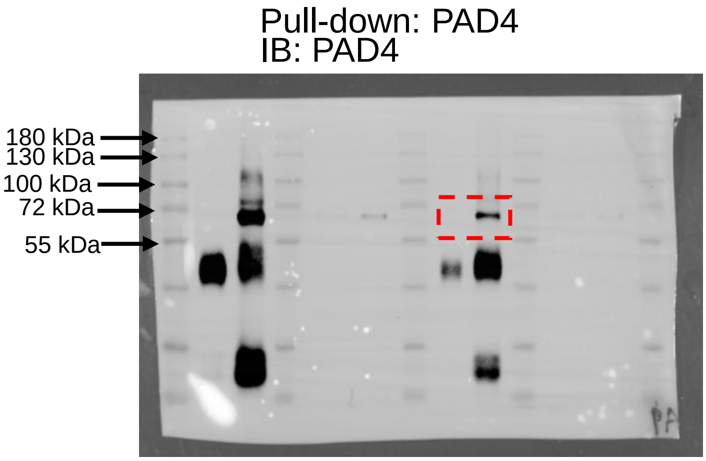

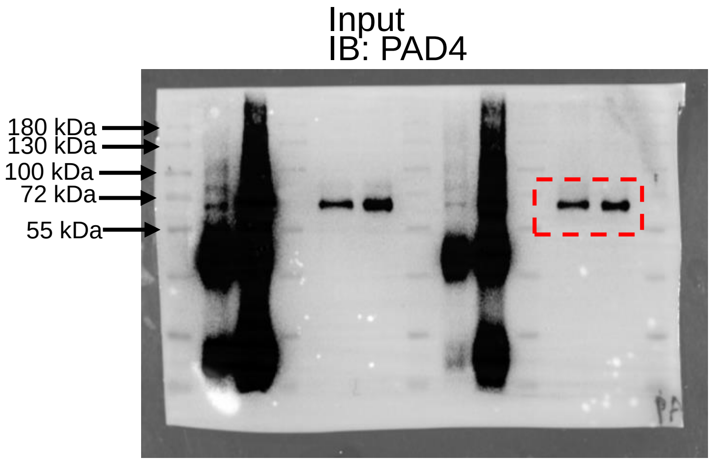

Full uncropped blots related to **Figure 7A**

IB: H3cit

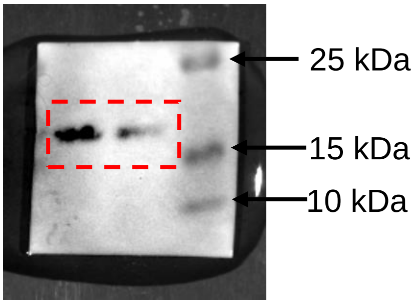

IB: TGM2-HA

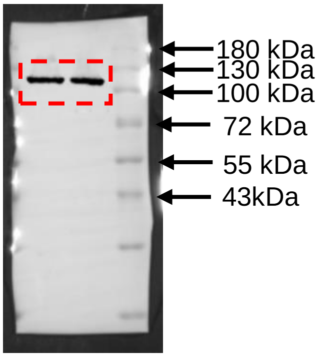

IB: H3Q5ser

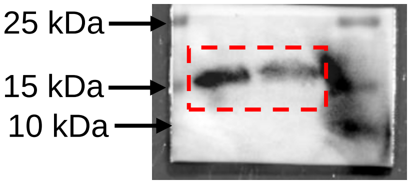

IB: PAD4-FLAG

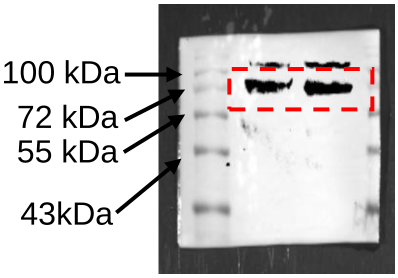

IB: H3

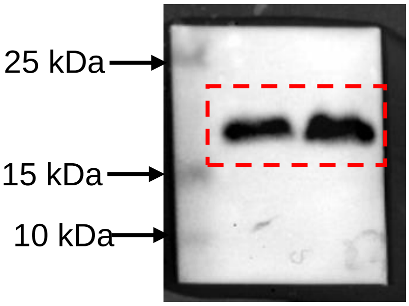

IB: H3.3-HA

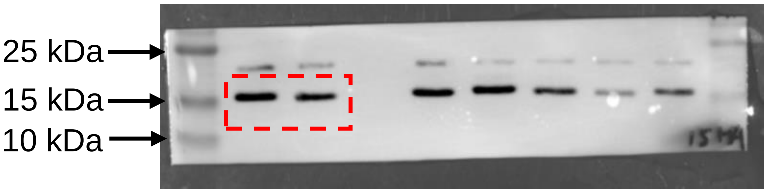

Full uncropped blots related to **Figure 7B**

IB: H3cit

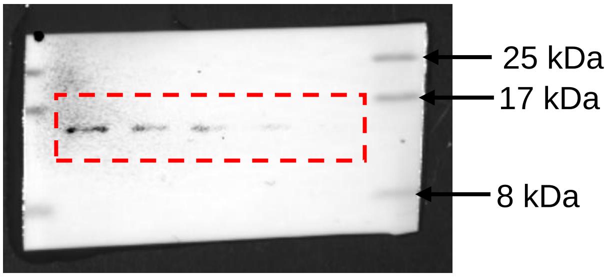

IB: TGM2-HA

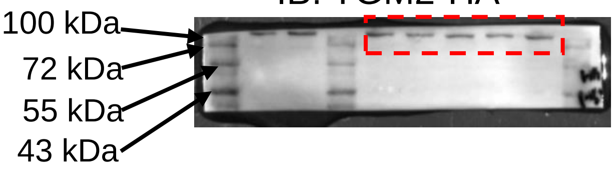

IB: PAD4-FLAG

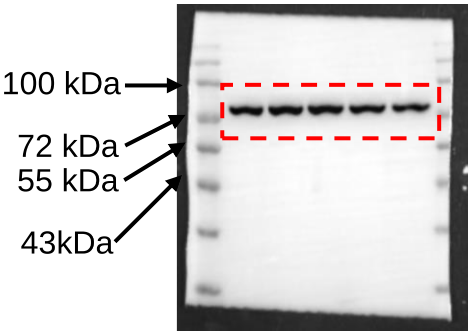

IB: H3Q5ser

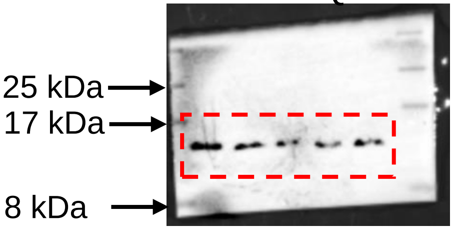

IB: H3.3-HA

IB: H3

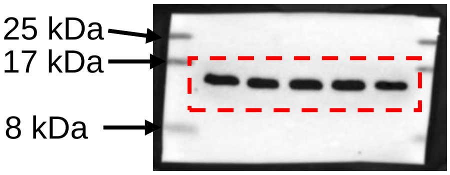

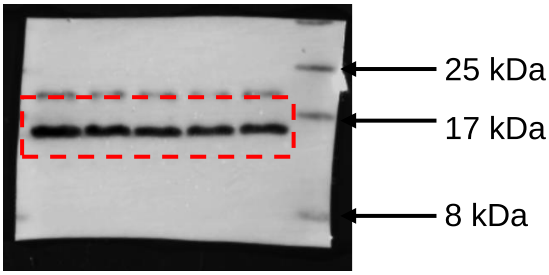

Full uncropped blots related to **Figure 7C**

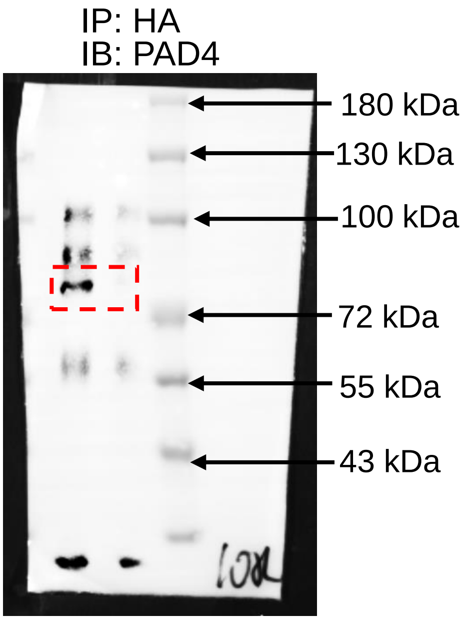

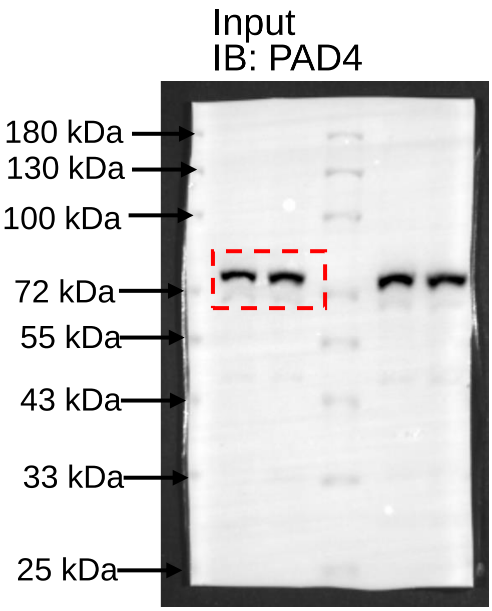

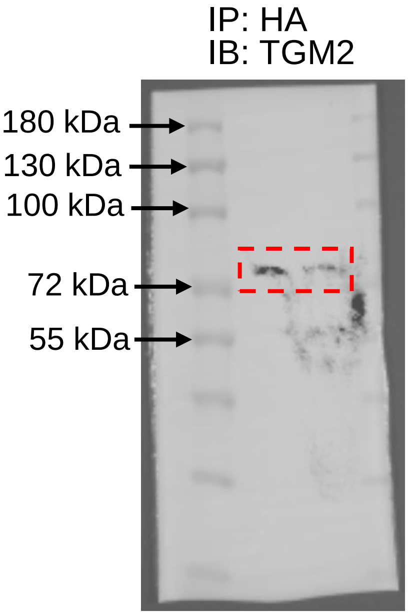

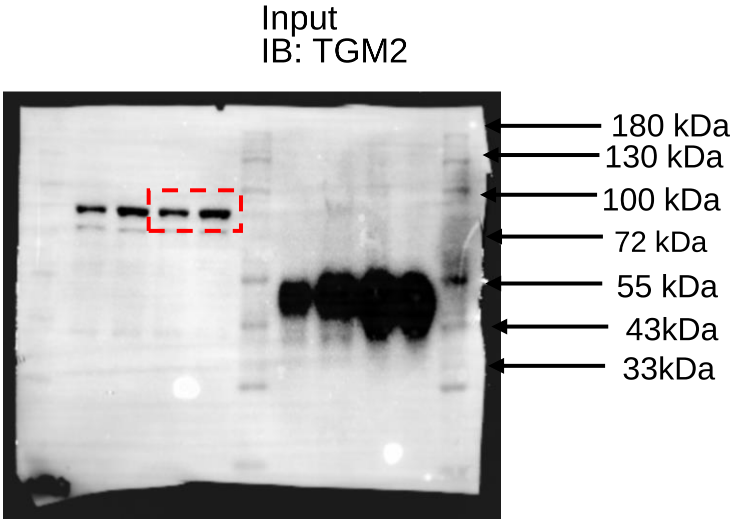

Full uncropped blots related to **Figure 7D**

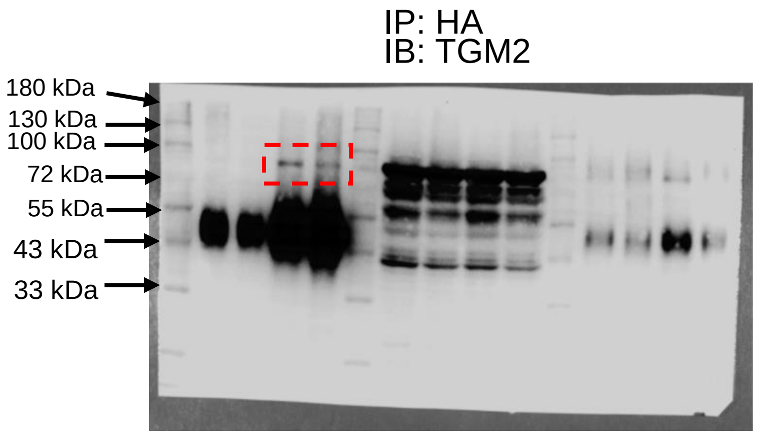

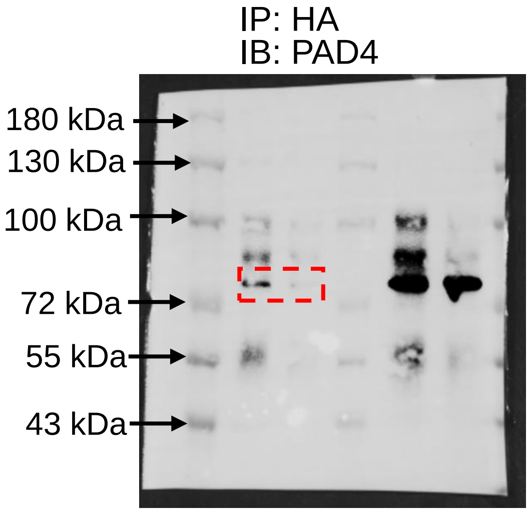

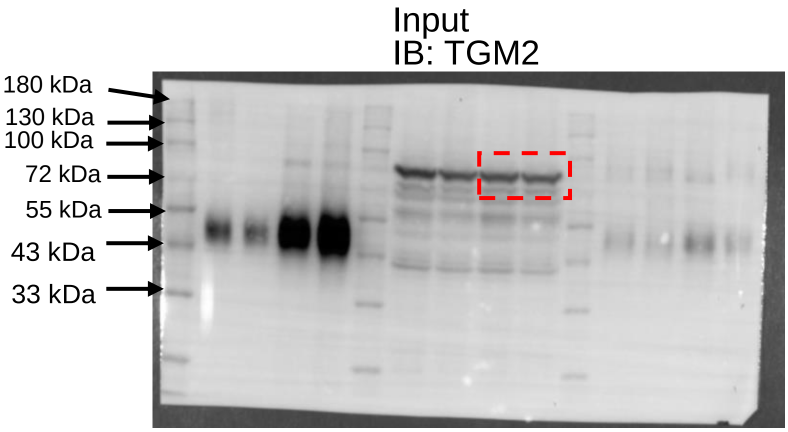

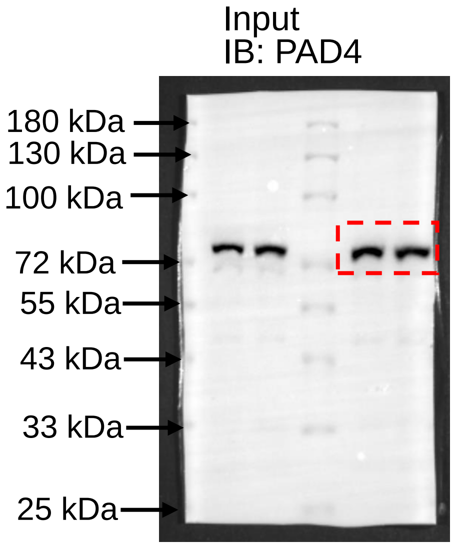

Full uncropped blots related to **Figure 7E**

IB: H3cit

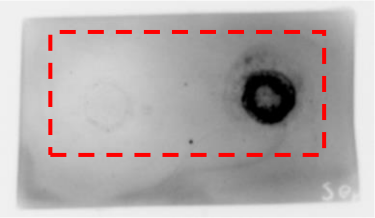

Ponceau S

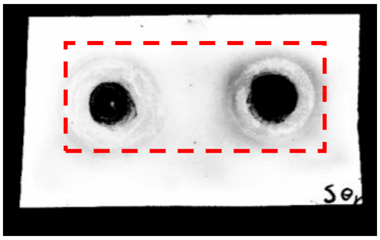

Full uncropped blots related to **Figure 7F**

IB: H3Q5ser

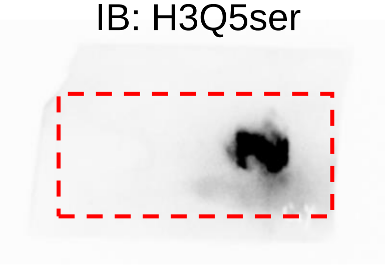

Ponceau S

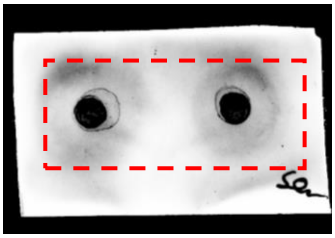

Full uncropped blots related to **Figure 7G**

IB: H3Q5ser

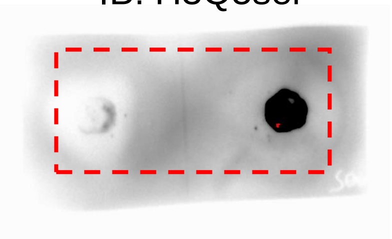

Ponceau S

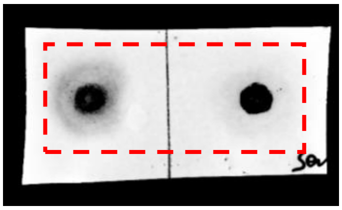

Full uncropped blots related to **Figure 7H**

IB: H3cit

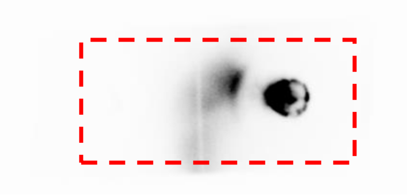

Ponceau S

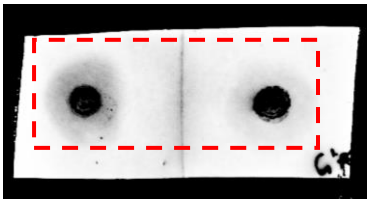

Full uncropped blots related to **Supplementary Figure 3B**

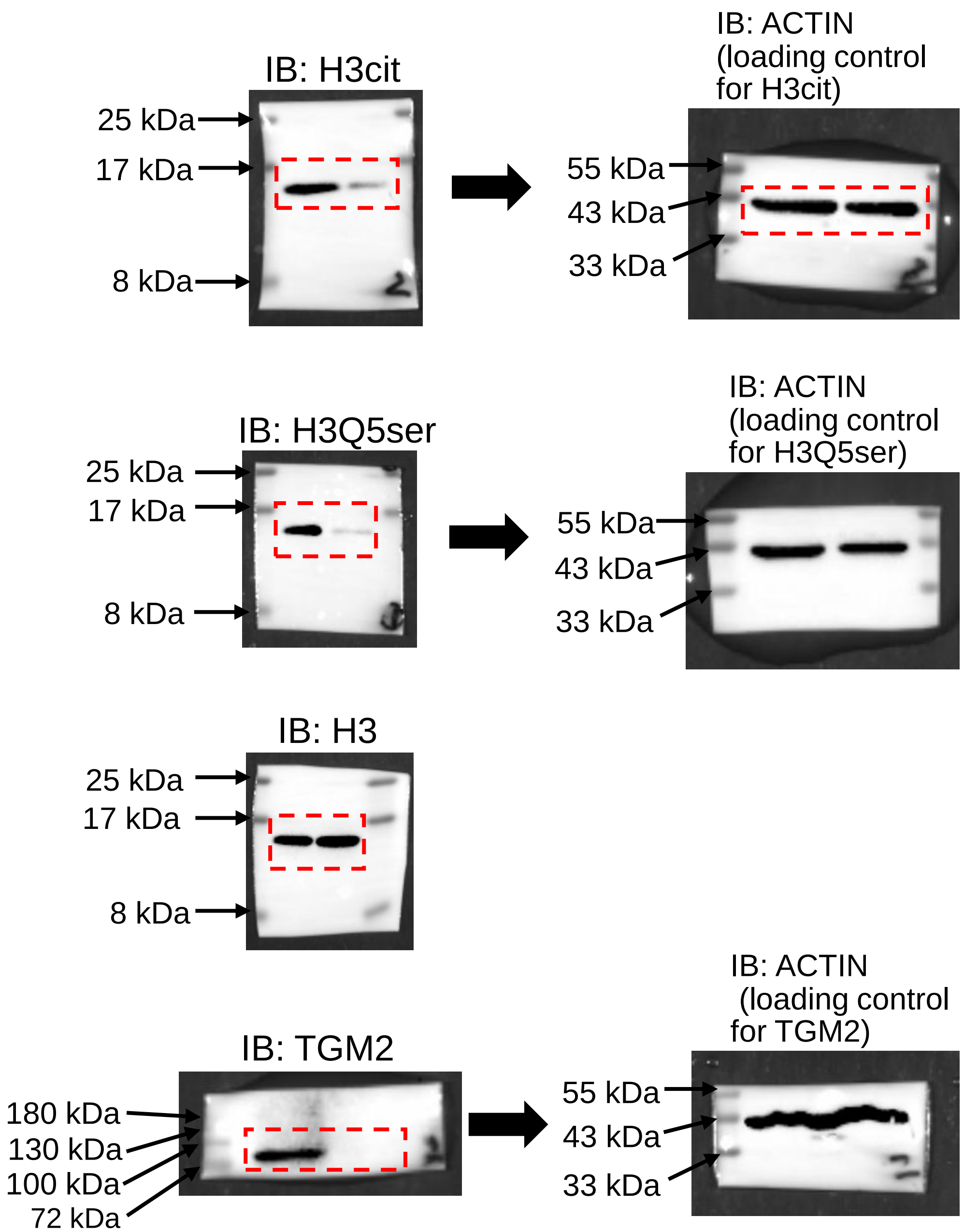

Full uncropped blots related to **Supplementary Figure 7A**

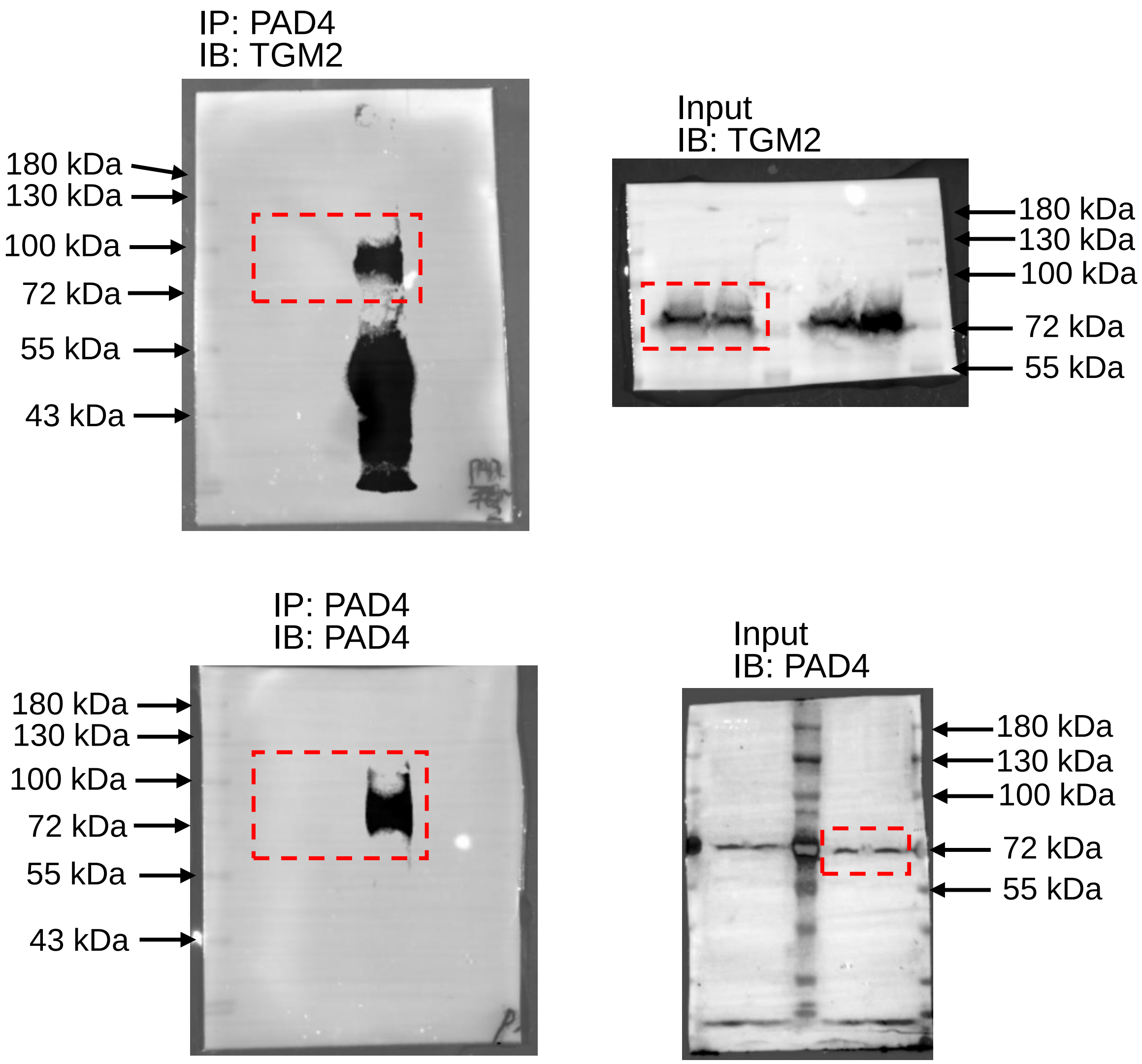

Full uncropped blots related to **Supplementary Figure 7B**

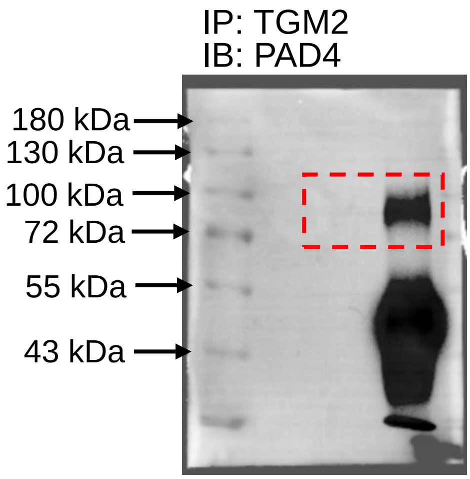

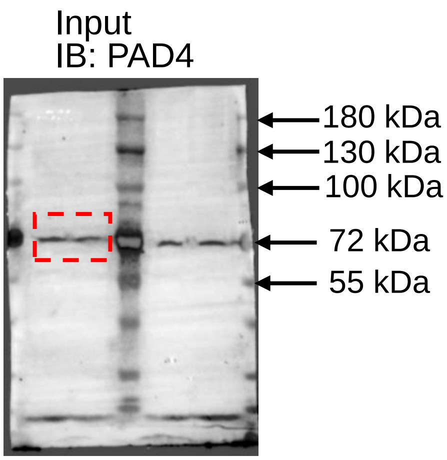

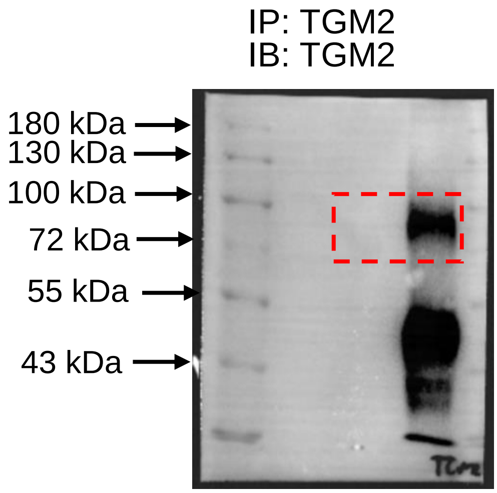

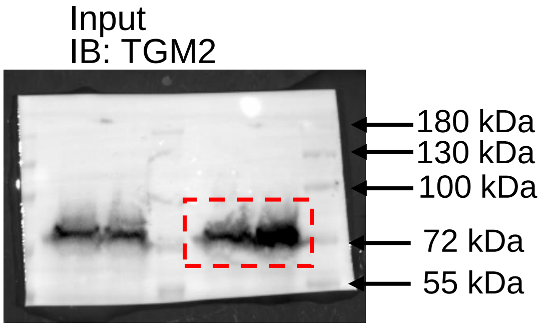

Full uncropped blots related to **Supplementary Figure 7E**

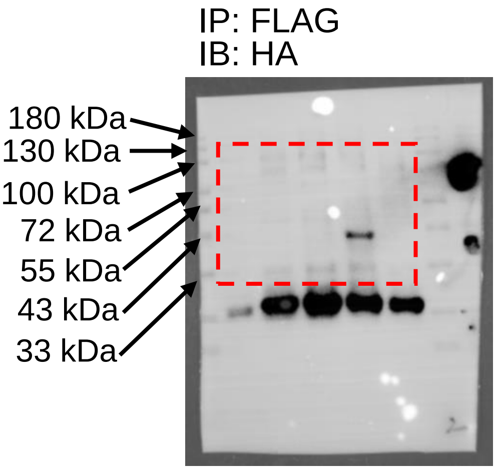

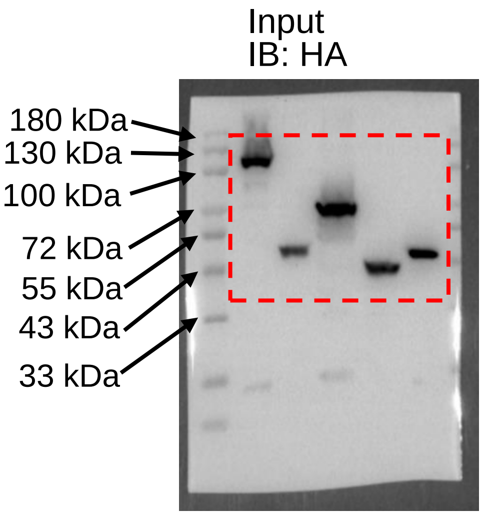

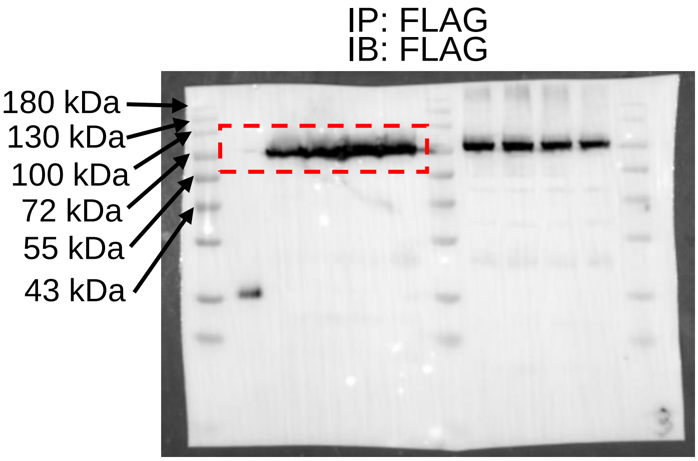

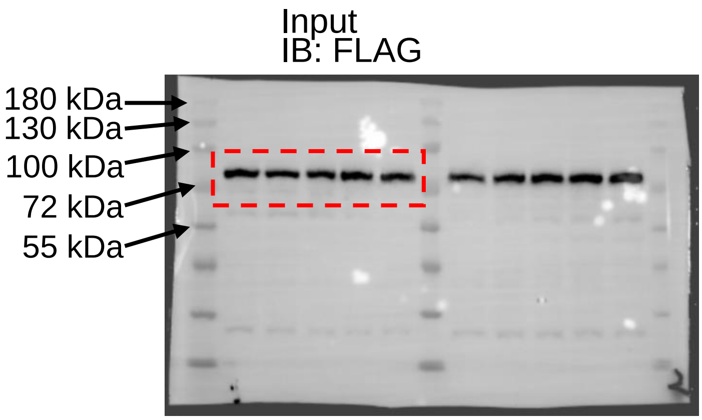

Full uncropped blots related to **Supplementary Figure 7F**

IP: HA
IB: FLAG

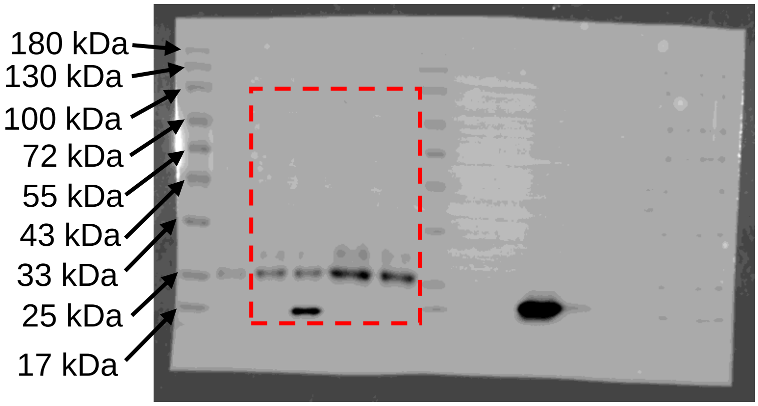

Input
IB: FLAG

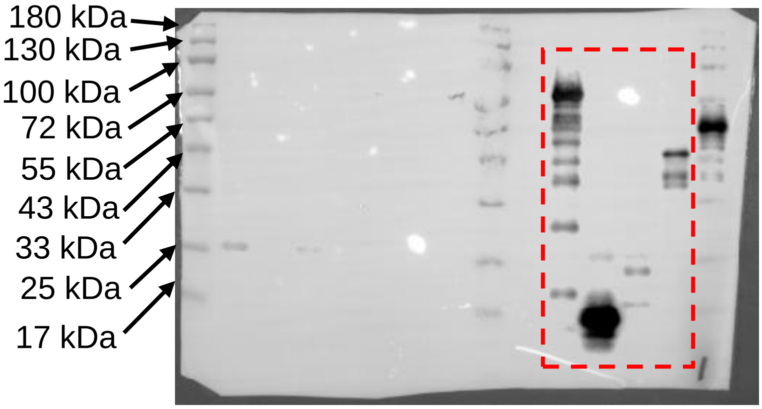

IP: HA
IB: HA

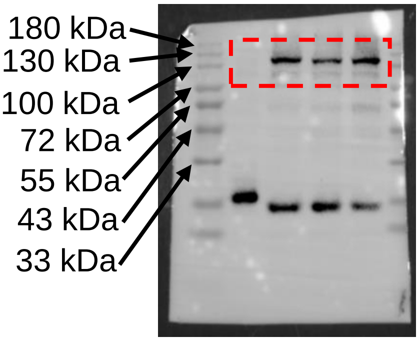

Input
IB: HA

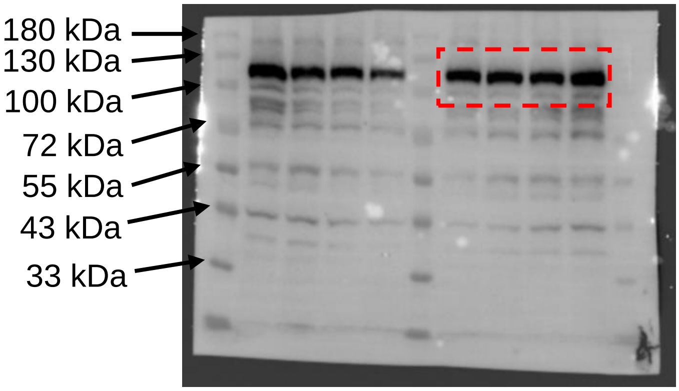

Full uncropped blots related to **Supplementary Figure 7G**

IP: PAD4-FLAG
IB: TGM2-HA

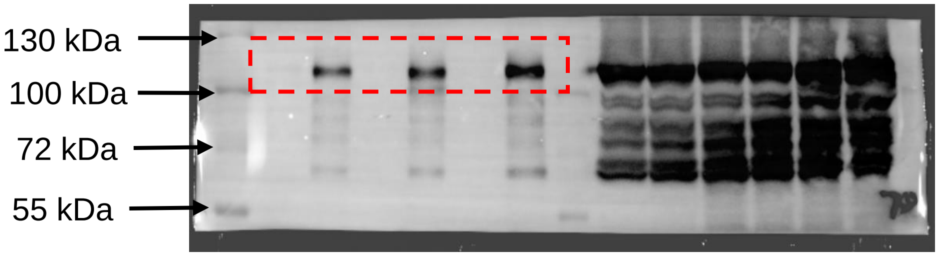

IP: PAD4-FLAG
IB: PAD4-FLAG

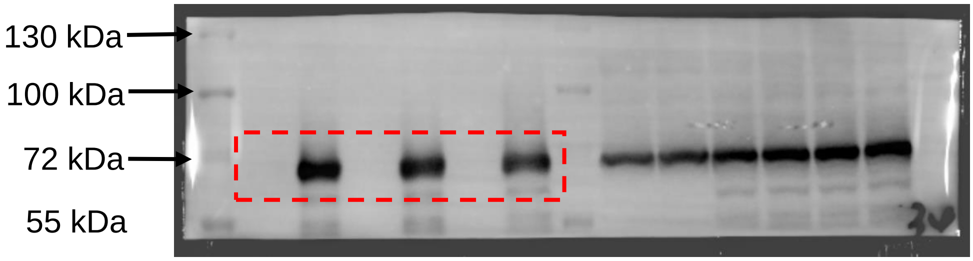

Input
IB: TGM2-HA

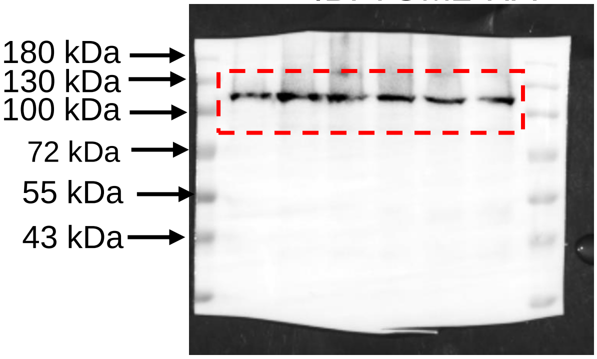

Input
IB: PAD4-FLAG

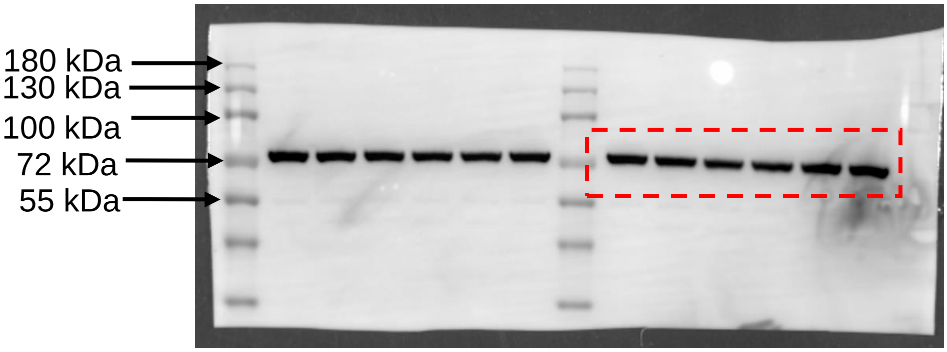

Full uncropped blots related to **Supplementary Figure 7H**

IP: TGM2-HA
IB:PAD4-FLAG

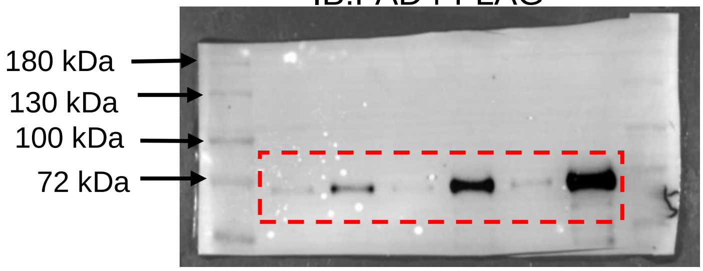

IP: TGM2-HA
IB: TGM2-HA

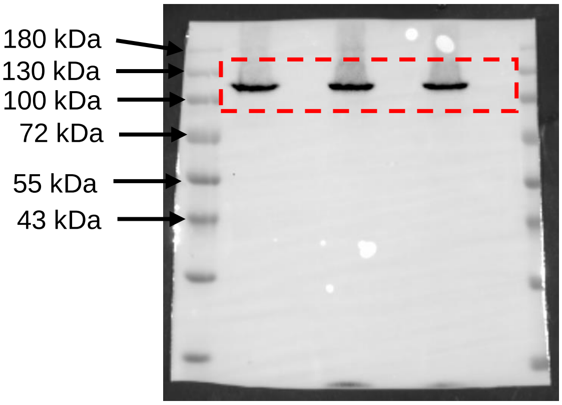

Input
IB: PAD4-FLAG

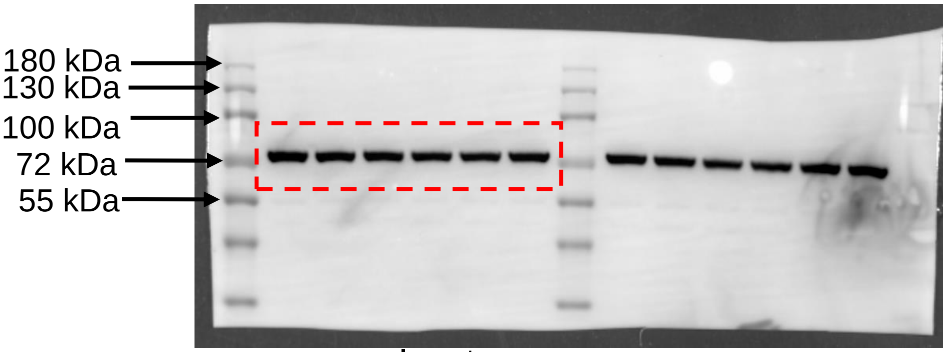

Input
IB: TGM2-HA

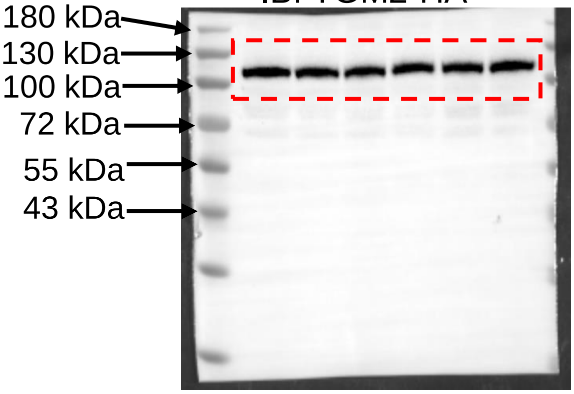

Full uncropped blots related to **Supplementary Figure 8C**

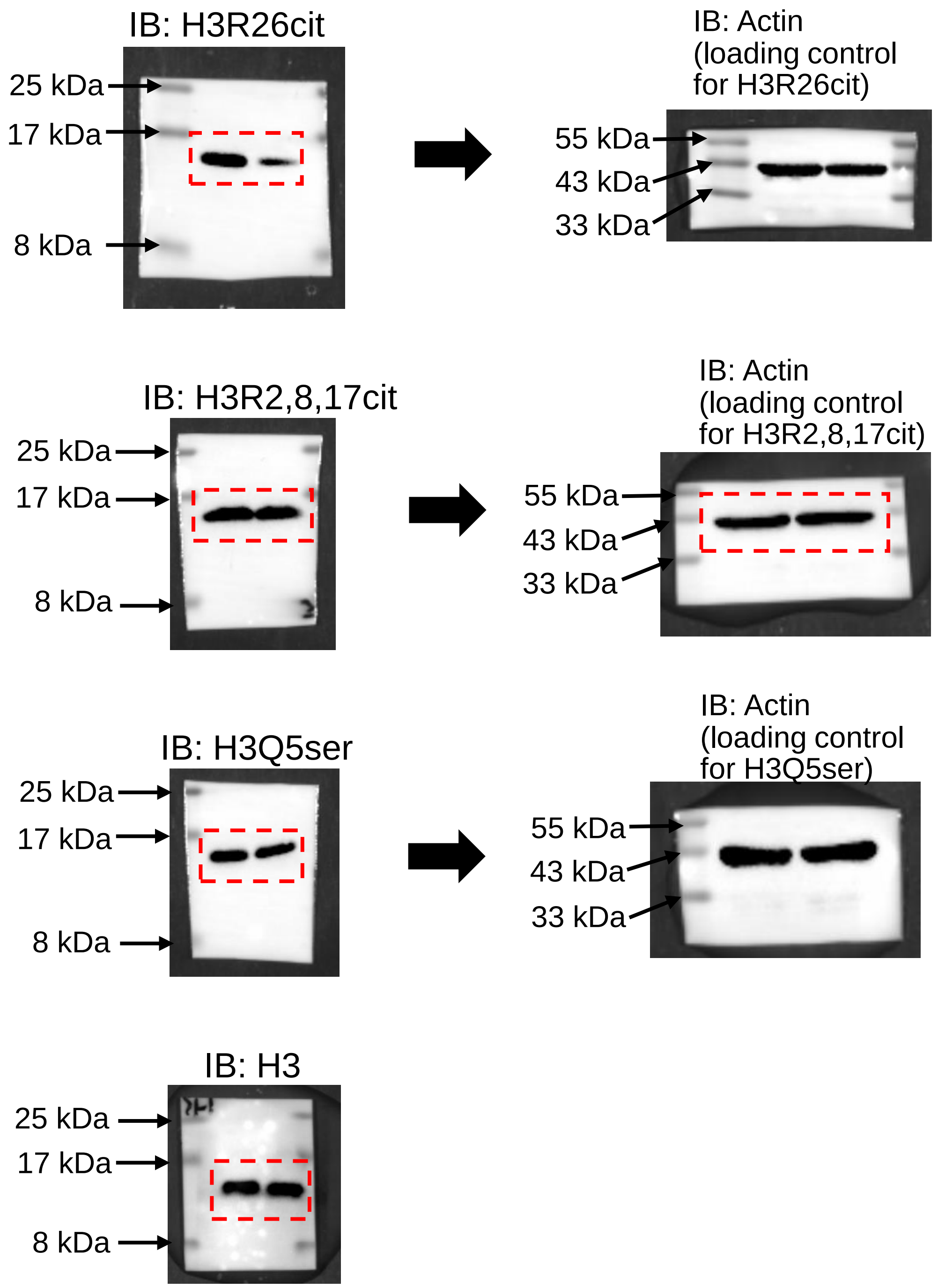

Full uncropped blots related to **Supplementary Figure 8C**

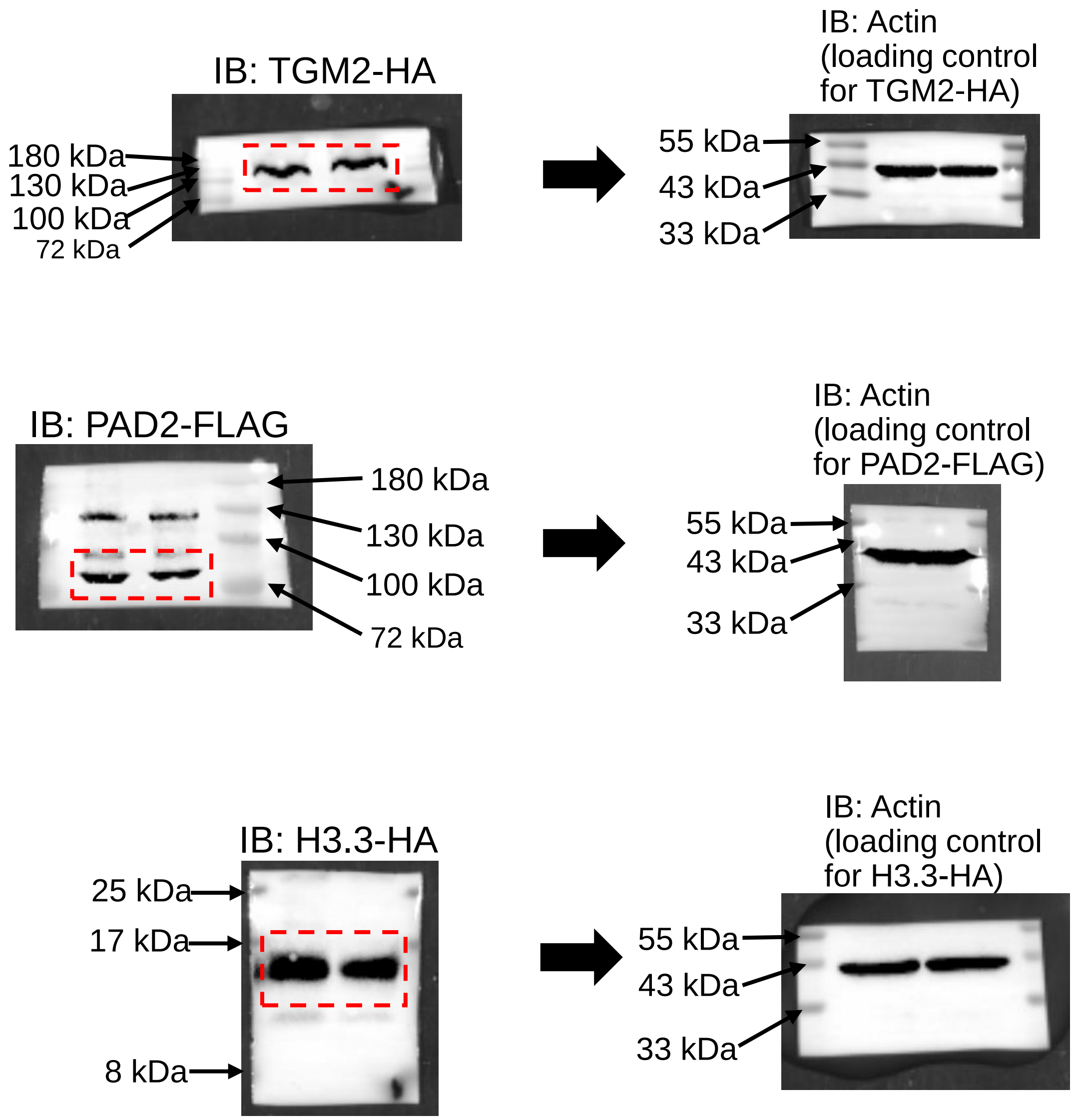

Full uncropped blots related to **Supplementary Figure 8D**

IP: PAD2-FLAG
IB: TGM2-HA

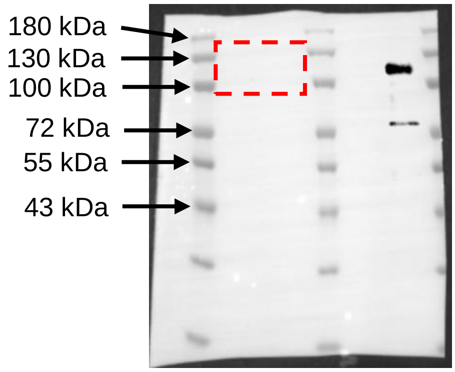

IP: PAD2-FLAG
IB: PAD2-FLAG

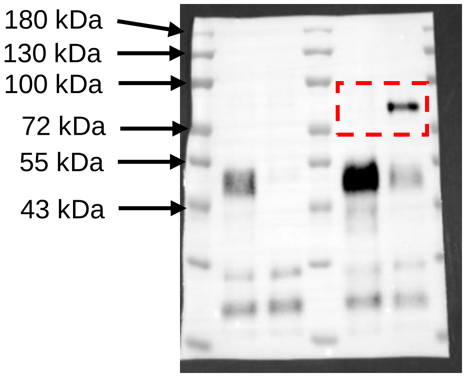

Input
IB: TGM2-HA

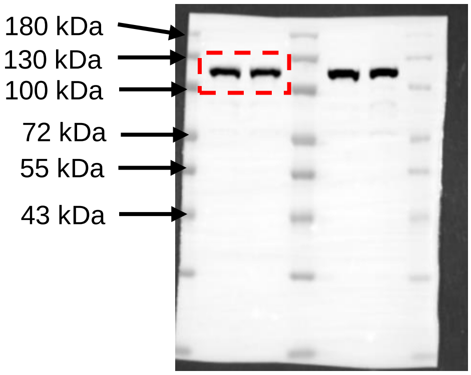

Input
IB: PAD2-FLAG

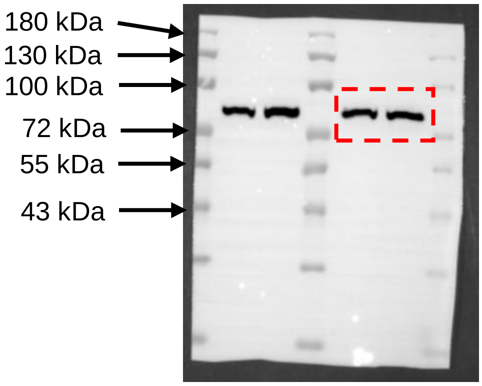

Full uncropped blots related to **Supplementary Figure 8E**

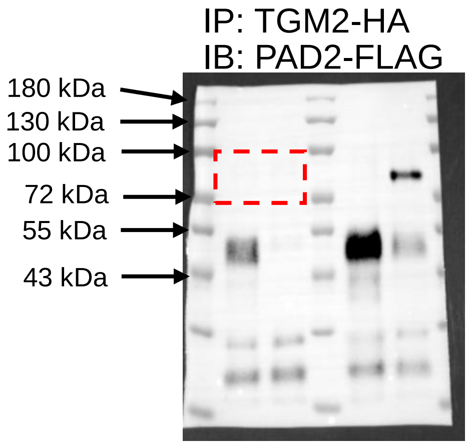

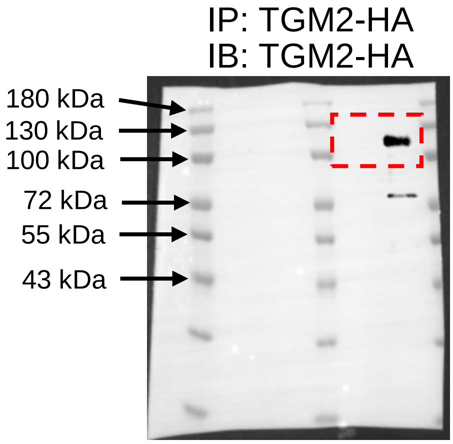

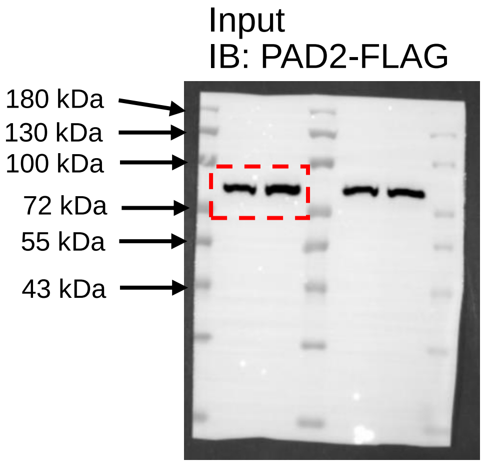

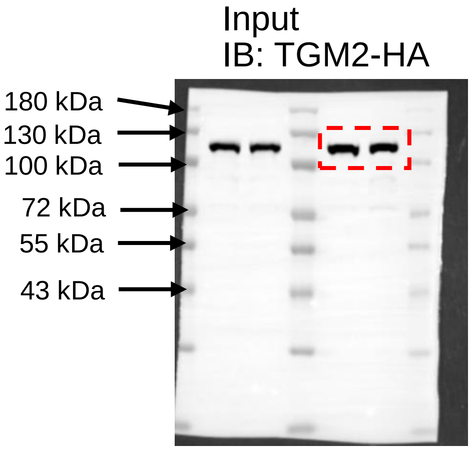

Supplement: Unedited blot and gel images [file jci-135-183544-s276.pdf]
